# Dihydroartemisinin-piperaquine for intermittent preventive treatment of malaria during pregnancy and risk of malaria in early childhood: A randomized controlled trial

**DOI:** 10.1371/journal.pmed.1002606

**Published:** 2018-07-17

**Authors:** Prasanna Jagannathan, Abel Kakuru, Jaffer Okiring, Mary K. Muhindo, Paul Natureeba, Miriam Nakalembe, Bishop Opira, Peter Olwoch, Felistas Nankya, Isaac Ssewanyana, Kevin Tetteh, Chris Drakeley, James Beeson, Linda Reiling, Tamara D. Clark, Isabel Rodriguez-Barraquer, Bryan Greenhouse, Erika Wallender, Francesca Aweeka, Mary Prahl, Edwin D. Charlebois, Margaret E. Feeney, Diane V. Havlir, Moses R. Kamya, Grant Dorsey

**Affiliations:** 1 Department of Medicine, Stanford University, Stanford, California, United States of America; 2 Infectious Diseases Research Collaboration, Kampala, Uganda; 3 Department of Obstetrics and Gynecology, Makerere University College of Health Sciences, Kampala, Uganda; 4 Department of Immunology and Infection, London School of Hygiene & Tropical Medicine, London, United Kingdom; 5 Burnet Institute, Melbourne, Australia; 6 Department of Medicine, University of California, San Francisco, San Francisco, California, United States of America; 7 Department of Clinical Pharmacy, University of California, San Francisco, San Francisco, California, United States of America; 8 Department of Pediatrics, University of California, San Francisco, San Francisco, California, United States of America; 9 Center for AIDS Prevention Studies, University of California, San Francisco, San Francisco, California, United States of America; 10 Department of Medicine, Makerere University College of Health Sciences, Kampala, Uganda; Mahidol-Oxford Tropical Medicine Research Unit, THAILAND

## Abstract

**Background:**

Intermittent preventive treatment of malaria in pregnancy (IPTp) with dihydroartemisinin-piperaquine (IPTp-DP) has been shown to reduce the burden of malaria during pregnancy compared to sulfadoxine-pyrimethamine (IPTp-SP). However, limited data exist on how IPTp regimens impact malaria risk during infancy. We conducted a double-blinded randomized controlled trial (RCT) to test the hypothesis that children born to mothers given IPTp-DP would have a lower incidence of malaria during infancy compared to children born to mothers who received IPTp-SP.

**Methods and findings:**

We compared malaria metrics among children in Tororo, Uganda, born to women randomized to IPTp-SP given every 8 weeks (SP8w, *n* = 100), IPTp-DP every 8 weeks (DP8w, *n* = 44), or IPTp-DP every 4 weeks (DP4w, *n* = 47). After birth, children were given chemoprevention with DP every 12 weeks from 8 weeks to 2 years of age. The primary outcome was incidence of malaria during the first 2 years of life. Secondary outcomes included time to malaria from birth and time to parasitemia following each dose of DP given during infancy. Results are reported after adjustment for clustering (twin gestation) and potential confounders (maternal age, gravidity, and maternal parasitemia status at enrolment).The study took place between June 2014 and May 2017. Compared to children whose mothers were randomized to IPTp-SP8w (0.24 episodes per person year [PPY]), the incidence of malaria was higher in children born to mothers who received IPTp-DP4w (0.42 episodes PPY, adjusted incidence rate ratio [aIRR] 1.92; 95% CI 1.00–3.65, *p* = 0.049) and nonsignificantly higher in children born to mothers who received IPT-DP8w (0.30 episodes PPY, aIRR 1.44; 95% CI 0.68–3.05, *p* = 0.34). However, these associations were modified by infant sex. Female children whose mothers were randomized to IPTp-DP4w had an apparently 4-fold higher incidence of malaria compared to female children whose mothers were randomized to IPTp-SP8w (0.65 versus 0.20 episodes PPY, aIRR 4.39, 95% CI 1.87–10.3, *p* = 0.001), but no significant association was observed in male children (0.20 versus 0.28 episodes PPY, aIRR 0.66, 95% CI 0.25–1.75, *p* = 0.42). Nonsignificant increases in malaria incidence were observed among female, but not male, children born to mothers who received DP8w versus SP8w. In exploratory analyses, levels of malaria-specific antibodies in cord blood were similar between IPTp groups and sex. However, female children whose mothers were randomized to IPTp-DP4w had lower mean piperaquine (PQ) levels during infancy compared to female children whose mothers received IPTp-SP8w (coef 0.81, 95% CI 0.65–1.00, *p* = 0.048) and male children whose mothers received IPTp-DP4w (coef 0.72, 95% CI 0.57–0.91, *p* = 0.006). There were no significant sex-specific differences in PQ levels among children whose mothers were randomized to IPTp-SP8w or IPTp-DP8w. The main limitations were small sample size and childhood provision of DP every 12 weeks in infancy.

**Conclusions:**

Contrary to our hypothesis, preventing malaria in pregnancy with IPTp-DP in the context of chemoprevention with DP during infancy does not lead to a reduced incidence of malaria in childhood; in this setting, it may be associated with an increased incidence of malaria in females. Future studies are needed to better understand the biological mechanisms of in utero drug exposure on drug metabolism and how this may affect the dosing of antimalarial drugs for treatment and prevention during infancy.

**Trial registration:**

ClinicalTrials.gov number NCT02163447.

## Abstract 2

In a randomized controlled trial, Prasanna Jagannathan and colleagues compare the impact of intermittent preventive treatment of malaria in pregnancy using dihydroartemisinin-piperaquine versus sulfadoxine-pyrimethamine on malaria risk during childhood.

## Author summary

**Why was this study done?:**

- Intermittent preventive treatment of malaria in pregnancy (IPTp) with dihydroartemisinin-piperaquine (DP) has been shown to reduce the burden of malaria during pregnancy compared to the current standard of care, sulfadoxine-pyrimethamine (SP).
- However, although there is some evidence that malaria in pregnancy may alter malaria susceptibility in infants, limited data exist on the impact of different IPTp regimens on malaria during early childhood.
- We hypothesized that children born to mothers who received IPTp with DP would have a lower incidence of malaria during the first 2 years of life compared to children born to mothers who received IPTp with SP.

**What did the researchers do and find?:**

- We conducted a double-blinded randomized controlled trial between June 2014 and May 2017 comparing malaria metrics among 191 infants born to mothers randomized to receive IPTp with SP or IPTp with DP; children born to these mothers were given chemoprevention with DP every 12 weeks starting at 8 weeks of age and followed to 2 years of age.
- We found that children born to mothers given IPTp with DP did not have a lower incidence of malaria in infancy; in fact, children born to mothers who received IPTp with DP every 4 weeks in pregnancy had a significantly higher incidence of malaria and *Plasmodium falciparum* infection in infancy.
- We found that this increased incidence of malaria was only observed in female infants; furthermore, female children born to mothers who received IPTp with DP every 4 weeks in pregnancy had significantly lower piperaquine drug levels during infancy compared to male children.

**What do these findings mean?:**

- Although IPTp with DP is more effective in preventing malaria in pregnancy compared to IPTp with SP, in this setting it was not associated with a lower risk of malaria in infancy.
- Although the results show an increased risk of malaria in female infants, these results should be interpreted with caution given the small sample size, especially when considering sex-stratified results.
- Future studies are needed both to confirm the impact of different IPTp regimens on malaria risk in infants, including those in settings where children are not given intermittent chemoprevention in infancy, as well as to understand the biologic mechanisms by which in utero drug exposure may lead to sex-specific changes in drug metabolism.

## Introduction

Malaria during pregnancy remains a significant cause of morbidity and mortality in sub-Saharan Africa [1]. Although the majority of women living in endemic areas remain asymptomatic when infected with malaria parasites, these infections are associated with maternal anemia and adverse birth outcomes, including miscarriage, stillbirth, preterm birth, low birth weight (LBW), and infant mortality [2]. For pregnant women living in malaria-endemic areas, WHO recommends the use of long-lasting insecticidal nets (LLINs) and intermittent preventive treatment of malaria during pregnancy (IPTp) with sulfadoxine-pyrimethamine (SP) to prevent infection with malaria parasites and reduce the risk of adverse birth outcomes. However, there is concern for diminishing efficacy of these interventions because of the spread of vector resistance to the pyrethroid insecticides used in LLINs and widespread parasite resistance to SP [3,4]. Dihydroartemisinin-piperaquine (DP) has emerged as an attractive alternative to SP for IPTp, as it is highly efficacious and provides at least 4 weeks of posttreatment prophylaxis [5,6]. Recent randomized controlled trials (RCTs) from our group and others showed that, compared to IPTp-SP, IPTp-DP dramatically reduced risks of maternal parasitemia, symptomatic malaria, anemia, and placental malaria, and IPTp-DP was as safe and well tolerated as IPTp-SP [7,8]. However, there were minimal differences between the SP and DP groups in risks of adverse birth outcomes, including LBW and preterm birth. Furthermore, it remains unclear what impact these different IPTp regimens have on the risk of malaria during early childhood.

Several studies have reported associations between malaria in pregnancy and a child’s subsequent risk of malaria, with mixed findings. Many have reported that infants born to mothers with placental malaria [9–14] or peripheral parasitemia during pregnancy [15] have a significantly higher risk of malaria during infancy. However, one study found a lower risk of malaria in infancy among infants born to primigravid mothers with placental malaria than infants born to primigravid mothers without placental malaria [14], and others have reported no significant association between malaria in pregnancy and the risk of malaria in infancy [16–18]. Notably, all of these studies were observational and any associations observed were subject to confounding by shared levels of exposure to malaria-infected mosquitoes between mothers and their infants [16,19].

If malaria in pregnancy alters a child’s subsequent risk of malaria, one would hypothesize that maternal receipt of effective IPTp might alter this risk, although few studies have examined this question with rigorous randomized controlled studies. In an older, placebo-controlled trial of IPTp with mefloquine in Thailand, infants followed after birth whose mothers had either received mefloquine or placebo had similar risks of *Plasmodium falciparum* infection and symptomatic disease [20]. Three more recent IPTp trials in Africa comparing IPTp-SP + LLIN with LLIN alone [12], IPTp-SP with mefloquine [21], or IPTp-SP with intermittent screening and treatment (IST) with artemether-lumefantrine (AL) [18] also found that the incidence of malaria from birth to 12 months of age was not significantly different between groups of children. However, in each of these studies, the experimental IPTp group was not shown to significantly reduce the burden of placental malaria compared to controls [22–24].

We conducted a double-blinded RCT to evaluate the impact of different IPTp regimens on the risk of malaria in infancy. Pregnant women were randomized to receive IPTp with SP every 8 weeks (IPTp-SP8w), IPTp with DP every 8 weeks (IPTp-DP8wk), or IPTp with DP every 4 weeks (IPTp-DP4w). Children born to these mothers were given chemoprevention with DP every 12 weeks starting at 8 weeks of age and followed to 2 years of age. We tested the hypothesis that children born to mothers who received IPTp with DP would have a lower incidence of malaria during the first 2 years of life compared to children born to mothers who received IPTp with SP.

## Methods

### Ethics statement

The study was funded by the National Institutes of Health and approved by the Institutional Review Boards of the Makerere University School of Biomedical Sciences, the Uganda National Council for Science and Technology, and the University of California, San Francisco. Written informed consent was obtained from all study participants.

### Study setting and participants

The study was conducted in Tororo district, Uganda, from June 2014 through May 2017. Tororo district is an area of historically high malaria transmission intensity with perennial transmission and an estimated entomologic inoculation rate of 310 infectious bites per person-year in 2013 [25]. Following a universal LLIN campaign in November 2013, near universal LLIN coverage was reported in Tororo district, with minimal change in malaria metrics after LLIN distribution [26]. From December 2014 to February 2015, indoor residual spraying (IRS) using the carbamate bendiocarb was initiated in Tororo district for the first time and was associated with significant reductions in malaria transmission [26,27]; a second round of bendiocarb was conducted in June–July 2015, and a third round in November–December 2015. A fourth round of IRS was conducted in June–July 2016 with pyrimiphos-methyl (Actellic), a long-lasting organophosphate.

This study was divided into 2 phases, the first phase randomizing pregnant women to different IPTp regimens and the second phase—the focus of this analysis—following children born from these mothers to 2 years of age. In the first phase of this study, pregnant women were screened and enrolled between June 2014 and October 2014. Eligible mothers were not infected with HIV and were of all gravidities, with an estimated gestational age between 12 and 20 weeks, confirmed by ultrasound, and provided written informed consent. Complete entry criteria are provided (see S1 Study Protocol) and have been previously described [28]. In the second phase of this study, children were born between October 2014 and May 2015 and followed through 2 years of age, with the last participant followed through May 2017.

### Study design and randomization

This was a double-blinded RCT of pregnant women not infected with HIV and the children born to them. Women and their unborn child(ren) were randomized to one of five treatment arms, including both the intervention for the woman during pregnancy and her unborn child(ren) during infancy, in a 2:1:1:1:1 randomization scheme, as follows: (1) women IPTp-SP8w, children DP every 12 weeks; (2) women IPTp-DP8w, children DP every 12 weeks; (3) women IPTp-DP8w, children DP every 4 weeks; (4) women IPTp-DP4w, children DP every 12 weeks; and (5) women IPTp-DP4w, children DP every 4 weeks. To compare the malaria risk among infants whose mothers were randomized to different IPTp regimens, the prespecified protocol-defined study population included only mother/infant pairs in one of the three study arms randomized to receive DP every 12 weeks during infancy, because we hypothesized that children randomized to receive DP every 4 weeks in infancy would be nearly completely protected against malaria in infancy [29].

A randomization list using permuted blocks of 6 or 12 was computer generated by a member of the study not directly involved in patient care. Study participants were randomized to their assigned treatment group at enrolment using premade, consecutively numbered, sealed envelopes. Non-singleton births from the same mother were assigned to the same intervention. Study pharmacists not otherwise involved in the study were responsible for treatment allocation and the preparation of study drugs.

### Study drugs

In pregnancy, each treatment with SP (Kamsidar, Kampala Pharmaceutical Industries, 500/25 mg tablets) consisted of 3 tablets given as a single dose. Each treatment with DP in pregnancy (Duo-Cotexin, Holley-Cotec, Beijing, China, 40 mg/320 mg tablets) consisted of 3 tablets given once a day for 3 consecutive days. Participants allocated IPTp-SP8w or IPTp-DP8w received active study drugs at 20, 28, and 36 weeks gestational age. Participants allocated IPTp-DP4w received active study drugs starting at 16 or 20 weeks gestational age. Placebos of SP and DP were used such that every 4 weeks participants received the same number of pills, with the same appearance.

Each treatment with DP in childhood consisted of half-strength tablets given once a day for 3 consecutive days (Duo-Cotexin, Holley-Cotec, Beijing, China, 20 mg/160 mg tablets), according to weight-based guidelines (see S1 Study Protocol). Infants randomized to receive DP every 12 weeks received placebo mimicking the dosing of DP every 4 weeks when they were not receiving study drug. The first day of each dose was directly observed in the clinic by study nurses, who had the flexibility to administer either intact or crushed tablets to the infants. Compliance with day 2 and day 3 dosing administered at home was assessed at the following monthly routine visit and was reported to be >99%.

### Study procedures

At enrolment, women received an LLIN and underwent a standardized examination. Pregnant women and their children received all of their medical care at a study clinic open every day. Study procedures for pregnant women have previously been described, with details available in S1 Study Protocol [8]. Briefly, during pregnancy, routine visits were conducted every 4 weeks, including collection of dried blood spots (DBS) for molecular testing, and women were encouraged to deliver at the hospital adjacent to the study clinic. At delivery, a standardized assessment was completed, including evaluation of birth weight and collection of biological specimens, including maternal blood, placental tissue, placental blood, and cord blood.

Following delivery, children were followed through 24 months of age and encouraged to come to the study clinic any time they were ill. Those who presented with a documented fever (tympanic temperature ≥38.0 ˚C) or history of fever in the previous 24 hours had blood collected for a thick blood smear. If the smear was positive, the patient was diagnosed with malaria and treated with AL. If the thick blood smear was negative, the patient was managed for a non-malarial febrile illness by study physicians. Episodes of uncomplicated malaria in children <4 months of age or weighing <5 kg, as well as episodes of complicated malaria and treatment failures within 14 days, were treated with either quinine or artesunate according to national malaria treatment guidelines. Routine visits were conducted every 4 weeks in children, including thick blood smears to assess for parasitemia by microscopy, collection of DBS for molecular testing, and collection of plasma by fingerprick for assessment of piperaquine (PQ) levels. Phlebotomy for routine laboratory tests, including complete blood count (CBC), was performed every 16 weeks. Adverse events were assessed and graded according to standardized criteria at every visit to the study clinic [30].

### Clinical laboratory procedures

Blood smears were stained with 2% Giemsa and read by experienced laboratory technologists. A blood smear was considered negative when the examination of 100 high-power fields did not reveal asexual parasites. For quality control, all slides were read by a second microscopist, and a third reviewer would settle any discrepant readings. DBS were tested for the presence of malaria parasites using a loop-mediated isothermal amplification (LAMP) kit (Eiken Chemical, Japan).

### Antibody measurements

IgG responses to 19 parasite surface antigens were measured in maternal and cord blood collected at delivery, including circumsporozoite protein (CSP), erythrocyte binding antigen (EBA) 140 region III-V (RIII-V), EBA175 RIII-V [31,32], EBA181 RIII-V, Early transcribed membrane protein (Etramp) 4, Etramp 5, gametocyte exported protein (GEXP18), H103/merozoite surface protein (MSP) 11, Heat shock protein 40 (HSP40), Plasmodium exported protein (Hyp2) [33]), MSP 2 (Ch150/9 and Dd2 alleles) [34], Apical membrane antigen 1 (AMA1) [31,32,35], glutamate rich protein (GLURP-R2)[36], MSP1-19 [31,32,37], Schizont egress antigen (SEA)-1 [38], Reticulocyte-binding protein homologue (Rh)2_2030, Rh4.2, and skeleton-binding protein 1 (SBP1). Glutathione S-transferase (GST) and Tetanus toxoid were used as controls. Luminex magnetic microsphere conjugation was performed by standard methods [39]. Fifty microliters thawed plasma (1/1,000 dilution) were coincubated with microsphere mixtures on a 96-well plate for 90 minutes, washed, then stained with 50 uL of 1/200 R-Phycoerythrin-conjugated AffiniPure F(ab’)2 Goat anti-human IgG (Jackson Immuno Research Laboratories) secondary antibody. Samples were then suspended in 100 uL PBS and read by the Luminex MAGPIX system. Positive control samples from individuals (*n* = 20) with known antibodies to these antigens were included on each plate. Standard curves were generated through serial dilutions of the positive control pool. Antibody levels, expressed in arbitrary units (AUs), were obtained by regressing raw MFI onto the standard curve for each antigen present on every plate and results log transformed [40].

### PQ pharmacokinetic sample collection and quantitation

Children provided capillary blood samples at 3 consecutive routine visits performed every 4 weeks after they received the 8, 32, 56, and 92 week doses of DP in infancy.

Pharmacokinetic samples (*n* = 1,505) were centrifuged within 60 minutes at 2,000*g* for 10 minutes, and plasma was stored at −80°C prior to being processed for PQ quantitation. PQ concentrations were determined using high performance liquid chromatography tandem mass spectrometry, as described [41], with modifications to lower the calibration range to 0.5–50 ng/mL and a new calibration range of 10–1,000 ng/mL. The lower limit of quantification (LLOQ) was 0.5 ng/mL and the coefficient of variance was <10% for all quality control concentrations.

### Study end points

The primary outcome was the incidence of malaria from birth to 24 months of age. Treatments for malaria within 14 days of a prior episode were not considered incident events. Secondary outcomes included time to malaria from birth and time to parasitemia following receipt of each dose of DP; the incidence of complicated malaria; the incidence of hospitalizations/deaths; the incidence of non-malarial febrile illness (presentation within 14 days of a prior episode were not considered incident events); and the prevalence of anemia (Hb < 11 g/dL) during infancy. Measures of safety included the incidence of adverse events from birth through 2 years of age. Post hoc, exploratory outcomes included the relative intensity of malarial antibodies measured at delivery (maternal and cord); and PQ levels measured 4, 8, and 12 weeks following receipt of DP. The primary exposure variable was maternal IPTp assignment.

### Statistical analysis

To test the hypothesis that either IPTp-DP4w or IPTP-DP8w would be associated with a lower risk of malaria in infancy compared to SP, we assumed an incidence of malaria of 3–5 episodes per person year (PPY) among children whose mothers were randomized to IPTp-SP8w based on prior data before the implementation of IRS. Assuming 5% lost to follow-up, we had 80% power to show a 22%–28% reduction in the incidence of malaria among infants whose mothers were randomized to either IPTp-DP4w or IPTp-DP8w (2-sided significance level = 0.05).

Data were double-entered and verified in Microsoft Access and statistical analysis performed using Stata, version 14. All analyses were done using a modified intention-to-treat approach, including all children born (excluding stillbirths) and randomized to DP every 12 weeks with evaluable person-time of follow-up. Any premature withdrawal from the study prior to 2 years of age was assumed to be random. Comparisons of simple proportions were made using the chi-squared or Fisher’s exact test. Comparisons of incidence measures were made using a negative binomial regression model. We assessed for significant interaction (*p* < 0.10) with the primary outcome and the following potential effect modifiers: sex of the infant, gestational age of the infant at birth, and maternal gravidity. Where significant effect modification was noted, results were reported from stratified analysis. The cumulative risk of developing malaria from birth was estimated using the Kaplan–Meier product limit formula, and associations with exposure variables assessed using a cox proportional hazards model. The cumulative risk of developing malaria parasitemia following receipt of each dose of DP in infancy was estimated using a multilevel mixed-effects parametric survival model, accounting for clustering within individuals and mothers (twin gestation). Comparisons of proportions with repeated measures were made using mixed effects logistic regression models. In all analyses, estimates accounted for maternal clustering (twin gestation). Estimates were adjusted for potential confounders (maternal age, gravidity, and maternal parasitemia status at enrolment); both unadjusted and adjusted results were reported in tables; adjusted estimates are presented in the text.

In post hoc analyses, comparisons of log-transformed antibody levels between groups were performed using the student *t* test. For PQ measurements, relationships between mean population PQ concentrations, days since dosing, maternal randomization, and infant sex were assessed using generalized estimating equations with log link and robust standard errors accounting for repeated observations within individuals. Marginal estimates were produced using final models and shown graphically. In all analyses, *p* < 0.05 was considered statistically significant, without adjustment for multiple comparisons.

## Results

### Study participants and follow-up

**Fig 1:**
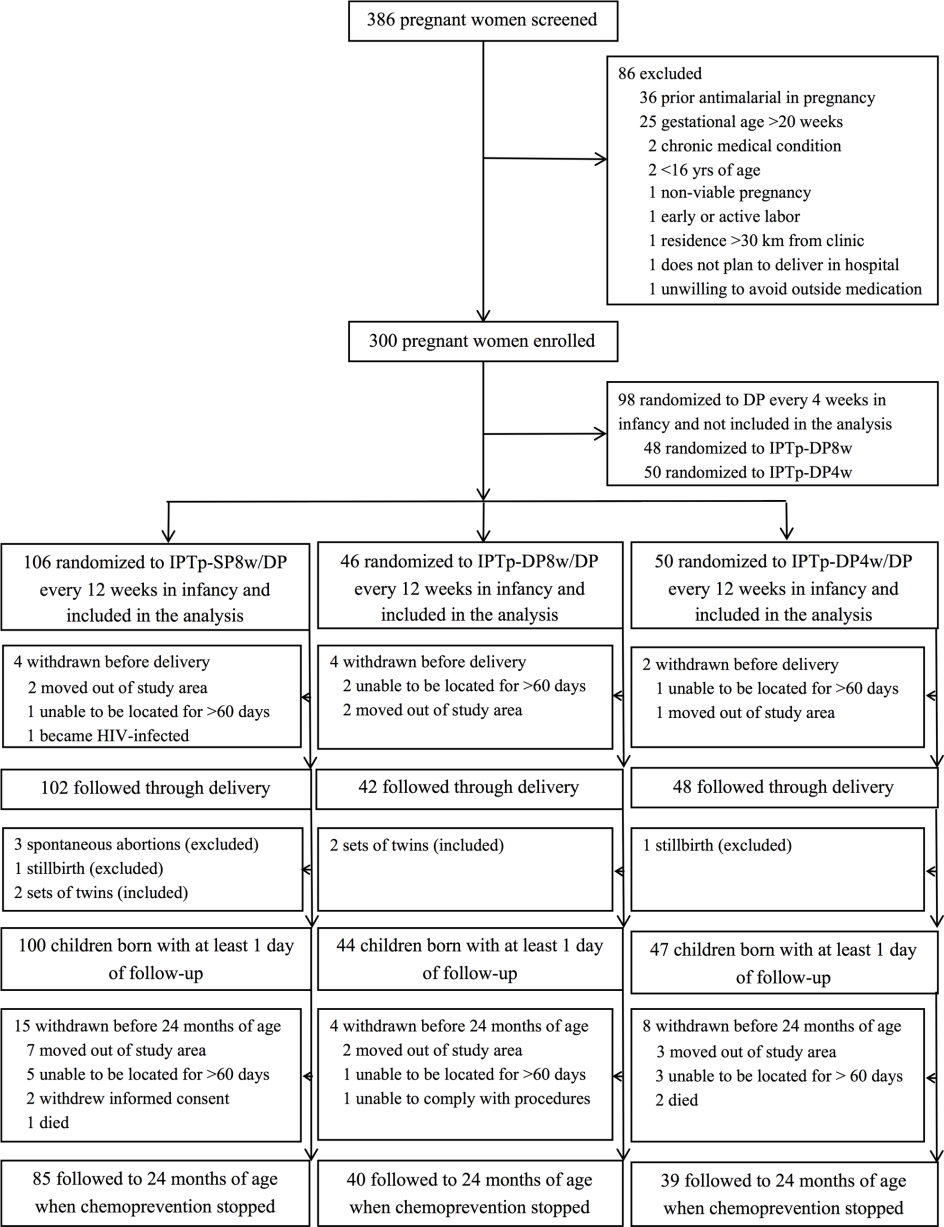
Trial profile. DP, dihydroartemisinin-piperaquine; IPTp-DP4w, IPTp-DP every 4 weeks; IPTp-DP8w, IPTp-DP given every 8 weeks; IPTp-SP8w, IPTp-SP given every 8 weeks.

Between June 2014 and October 2014, 386 pregnant women not infected with HIV and their unborn children were screened, and 300 were enrolled and randomized to one of five treatment arms defined by both maternal IPTp (SP8w, DP8w, or DP4w) and infant intermittent preventive treatment (IPT) (DP every 4 or 12 weeks beginning at 8 weeks of age, Fig 1). Among pregnant women/child pairs enrolled, 202 were randomized to receive DP every 12 weeks in infancy and included in this analysis. Of these, 106 women were randomized to IPTp-SP8w; 46 to IPTp-DP8w, and 50 to IPTp-DP4w. A total of 192 women (95.0%) were followed through delivery, and 4 women gave birth to twins. Of 194 live births, 191 children were followed for at least one day and included in the analyses. All children were born between October 2014 and May 2015.

Mean maternal age at enrolment was 22 years, 69% of women were enrolled at ≤16 weeks gestational age, 37% were primigravida, and 57% had malaria parasites detected by LAMP. Baseline maternal characteristics were not significantly different between the 3 IPTp arms, except for age, with women randomized to IPTp-SP8w slightly younger than women randomized to the other 2 treatment groups (Table 1). After initiation of IPTp, the incidence of malaria was significantly higher during pregnancy in mothers who received IPTp-SP8w (0.95 episodes PPY) compared to mothers who received IPTp-DP8w (0.23 episodes PPY, *p* = 0.004) or IPTp-DP4w (0 episodes PPY, *p* < 0.001). Similarly, parasite prevalence during pregnancy was significantly higher in mothers who received IPTp-SP8w compared to mothers who received IPTp-DP8w or IPTp-DP4w. The prevalence of placental malaria determined by LAMP or histopathology was significantly higher among women who received IPTp-SP8w compared to women who received IPTp with either IPTp-DP8w or IPTp-DP4w (Table 1).

**Table 1:** Characteristics of mother-child pairs stratified by mother’s IPTp treatment arm.

Characteristic	Mother’s IPTp treatment arm				
	SP8w (*n* = 100)*	DP8w (*n* = 44)		DP4w (*n* = 47)	
**Characteristics of mothers at enrolment**					
Age in years, mean (SD)	21.4 (3.6)	23.0 (4.1)		23.0 (3.8)	
Weeks of gestation, *n* (%)					
12–16 weeks	71 (71.0%)	29 (65.9%)		31 (66.0%)	
>16–20 weeks	29 (29.0%)	15 (34.1%)		16 (34.0%)	
Mother’s gravidity, n (%)					
1	35 (35.0%)	10 (22.7%)		10 (21.3%)	
2	33 (33.0%)	16 (36.4%)		16 (34.0%)	
≥3	32 (32.0%)	18 (40.9%)		21 (44.7%)	
Household wealth index, *n* (%)					
Lowest third	37 (37.0%)	11 (25.0%)		15 (31.9%)	
Middle third	29 (29.0%)	19 (43.2%)		15 (31.9%)	
Highest third	34 (34.0%)	14 (31.8%)		17 (36.2%)	
Detection of malaria parasites by LAMP^a^, *n* (%)	56 (56.0%)	19 (43.2%)		27 (57.5%)	
**Characteristics of mothers during pregnancy after initiation of study drugs**					
			*p*-value		*p*-value
Incidence of malaria^b^	0.95	0.23	0.004	0	<0.001
Parasite prevalence^c^	201/499 (40.3%)	24/203 (11.8%)	<0.001	8/239 (3.4%)	<0.001
Placental malaria by microscopy, n/N (%)	4/96 (4.2%)	1/44 (2.3%)	0.58	0/45 (0%)	0.17
Placental malaria by LAMP^a^, n/N (%)	19/96 (19.8%)	1/44 (2.3%)	0.006	0/45 (0%)	0.001
Placental malaria by histology, n/N (%)	49/97 (50.5%)	13/44 (29.6%)	0.02	10/45 (22.2%)	0.001
**Characteristics of children at birth**					
			*p*-value		*p*-value
Female sex, *n* (%)	51 (51.0%)	24 (54.6%)	0.70	23 (48.9%)	0.82
Gestational age in weeks at birth, mean (SD)	39.3 (1.8)	39.1 (2.6)	0.54	40.0 (1.2)	0.03
Preterm births (<37 weeks of gestation), *n* (%)	8 (8.0%)	5 (11.4%)	0.52	1 (2.1%)	0.17
Birth weight in grams, mean (SD)	2,967 (447)	2,928 (532)	0.65	3,024 (339)	0.44
Low birth weight (<2,500 gm), *n* (%)	13 (13.0%)	6 (13.6%)	0.92	2 (4.3%)	0.10

*Reference group.

^a^Loop amplified isothermal amplification.

^b^Episodes of malaria per person year at risk.

^c^Proportion of routine blood samples positive for malaria parasites by LAMP.

Abbreviations: DP4w, IPTp-DP every 4 weeks; DP8w, IPTp-DP given every 8 weeks; IPTp, intermittent preventive treatment of malaria in pregnancy; LAMP, loop-mediated isothermal amplification; SP8w, IPTp-SP given every 8 weeks.

Of 191 children born and followed, 98 (51.3%) were female, 14 (7.3%) were born preterm, and 21 (11%) were born with LBW (<2,500 g), and these characteristics were not significantly different based on maternal IPTp. Median gestation age at birth was slightly older for babies born to mothers who received IPTp-DP4w (median gestation age 40 weeks [35.7–41.6 weeks]) compared to those born to mothers who children whose mothers received IPTp-SP8w (median gestation age 39.3 weeks [31.7–42.0 weeks], *p* = 0.03, Table 1). Of 191 children followed, 164 (86%) completed follow-up through 2 years of age (Fig 1). When considering person-time of follow-up, 355.1 of 382 (93%) possible person years were observed.

### Impact of IPTp with DP on burden of malaria in infancy

During 355.1 person years of follow-up, there were 105 incident episodes of malaria in infants, giving an overall incidence of 0.30 episodes PPY. Compared to children born to mothers who received IPTp-SP8w, the incidence of malaria during 24 months of follow-up was higher in children born to mothers who received IPTp-DP4w (adjusted incidence rate ratio [aIRR] 1.92; 95% CI 1.00–3.65, *p* = 0.049) and nonsignificantly higher in children born to mothers who received IPT-DP8w (aIRR 1.44; 95% CI, 0.68–3.05; *p* = 0.34, Table 2). However, we observed significant effect modification by infant sex. Female children born to mothers who received IPTp-DP4w had a greater than 4-fold apparently higher incidence of malaria (aIRR 4.39; 95% CI, 1.87–10.3; *p* = 0.001) compared to female children born to mothers who received IPTp-SP8w. Similarly, female children born to mothers who received IPTp-DP8w had a higher, but nonsignificant, risk of malaria compared to female children born to mothers who received IPTp-SP8w (aIRR 2.43; 95% CI, 0.85–6.97; *p* = 0.10). In contrast, there were no significant differences in the incidence of malaria among male children across the 3 IPTp treatment arms (Table 2). No significant effect modification was observed with maternal gravidity or gestational age at birth.

**Table 2:** Incidence of malaria though 24 months of age by mother’s IPTp treatment arm overall and stratified by infant sex.

Sex	Mother’s IPTp treatment arm	Number of children	Episodes of malaria	PY of observation	Incidence of malaria PPY	IRR (95% CI)	*p*-value	aIRR^a^ (95% CI)	*p*-value
All^b^	SP8w	100	44	185.2	0.24	reference		reference	
	DP8w	44	25	84.2	0.30	1.29 (0.63–2.64)	0.49	1.44 (0.69–3.02)	0.33
	DP4w	47	36	85.5	0.42	1.81 (0.93–3.53)	0.08	1.92 (1.00–3.65)	0.049
Female	SP8w	51	18	91.3	0.20	reference		reference	
	DP8w	24	16	44.3	0.36	1.91 (0.65–5.64)	0.24	2.43 (0.85–6.97)	0.10
	DP4w	23	27	41.3	0.65	3.44 (1.41–8.40)	0.007	4.39 (1.87–10.3)	0.001
Male	SP8w	49	26	94.0	0.28	reference		reference	
	DP8w	20	9	40.0	0.23	0.82 (0.38–1.77)	0.62	0.84 (0.37–1.90)	0.67
	DP4w	24	9	44.2	0.20	0.74 (0.25–2.17)	0.58	0.66 (0.25–1.75)	0.41

^a^Adjusted for maternal age, gravidity, LAMP status at enrolment, and maternal clustering for twin gestation.

^b^Interaction *p*-value between female sex and IPTp arm: 0.22 for ITPp-DP8w; 0.03 for IPTp-DP4w.

Abbreviations: aIRR, adjusted incidence rate ratio; DP4w, IPTp-DP every 4 weeks; DP8w, IPTp-DP given every 8 weeks; IPTp, intermittent preventive treatment of malaria in pregnancy; IRR, incidence rate ratio; LAMP, loop-mediated isothermal amplification; PY, person year; PPY, person per year; SP8w, IPTp-SP given every 8 weeks.

Children born to mothers who received IPTp-DP4w had a nonsignificantly increased hazard of developing a first episode of malaria during the first 2 years of life in comparison to children born to mothers who received IPTp-SP8w (adjusted hazard ratio [aHR] 1.76, 95% CI 0.93–3.35, *p* = 0.08), but we again observed significant interaction with infant sex. Female infants whose mothers received IPTp-DP4w had a 3-fold apparently increased hazard of developing malaria compared to infants born to mothers who received IPTp-SP8w (aHR 3.40; 95% CI,1.55–7.96; *p* = 0.002, Table 3). Female children born to mothers who received IPTp-DP8w also had a higher, but nonsignificant, increased risk of malaria compared to those whose mothers received IPTp-SP8w; no significant associations were observed among male children across the 3 IPTp treatment arms (Table 3). These data do not support our original hypothesis that preventing malaria exposure in utero with DP leads to a reduced incidence of malaria in childhood; in contrast, in this setting it may be associated with an increased incidence of malaria in females.

**Table 3:** Time to first episode of malaria by mother’s IPTp treatment arm overall and stratified by infant sex.

Sex	Mother’s IPTp treatment arm	Number of children	Number with any malaria	Cumulative risk of any malaria (95% CI)	HR (95% CI)	*p*-value	aHR^a^ (95% CI)	*p*-value
All^b^	SP8w	100	26	28.4% (20.2%–38.3%)	reference		reference	
	DP8w	44	13	30.8% (19.2%–47.1%)	1.15 (0.59–2.26)	0.68	1.36 (0.67–2.76)	0.40
	DP4w	47	16	37.0% (24.6%–53.2%)	1.54 (0.82–2.91)	0.18	1.76 (0.93–3.35)	0.08
Female	SP8w	51	11	24.3% (14.3%–39.7%)	reference		reference	
	DP8w	24	5	22.4% (10.0%–45.7%)	0.97 (0.33–2.83)	0.96	1.21 (0.39–3.69)	0.74
	DP4w	23	11	52.4% (33.3%–74.3%)	2.97 (1.29–6.84)	0.01	3.40 (1.55–7.96)	0.002
Male	SP8w	49	15	32.3% (20.9%–47.8%)	reference		reference	
	DP8w	20	8	40.0% (22.4%–64.3%)	1.35 (0.58–3.18)	0.49	1.51 (0.59–3.88)	0.39
	DP4w	24	5	22.7% (10.1%–46.3%)	0.71 (0.25–1.98)	0.51	0.82 (0.29–2.32)	0.71

^a^Adjusted for maternal age, gravidity, LAMP status at enrolment, and maternal clustering for twin gestation.

^b^Interaction *p*-value between female sex and IPTp arm: 0.63 for ITPp-DP8w; 0.03 for IPTp-DP4w.

Abbreviations: aHR, adjusted hazard ratio; DP4w, IPTp-DP every 4 weeks; DP8w, IPTp-DP given every 8 weeks; HR, hazard ratio; IPTp, intermittent preventive treatment of malaria in pregnancy; LAMP, loop-mediated isothermal amplification; SP8w, IPTp-SP given every 8 weeks.

Given these differences, we assessed whether the incidence of malaria was different between male and female children. Overall, female children had a nonsignificantly increased incidence of malaria compared to males (aIRR 1.43, 95% CI 0.82–2.50, *p* = 0.21), but this effect was modified by maternal IPTp. Female children whose mothers were randomized to IPTp-DP4w had a >3-fold apparently increased risk of malaria compared to males (aIRR 3.34, 95% CI 1.08–10.26, *p* = 0.04). However, there were no significant sex-specific differences among children whose mothers were randomized to IPTp-SP8w (aIRR 0.72, 95% CI 0.31–1.67 comparing females to males) or IPTp-DP8w (aIRR 1.66, 95% CI 0.59–4.67 comparing females to males). These data suggest that, in this setting, female infants appear to have an increased risk of malaria in infancy compared to males, but only among those whose mothers received IPTp-DP4w.

**Fig 2:**
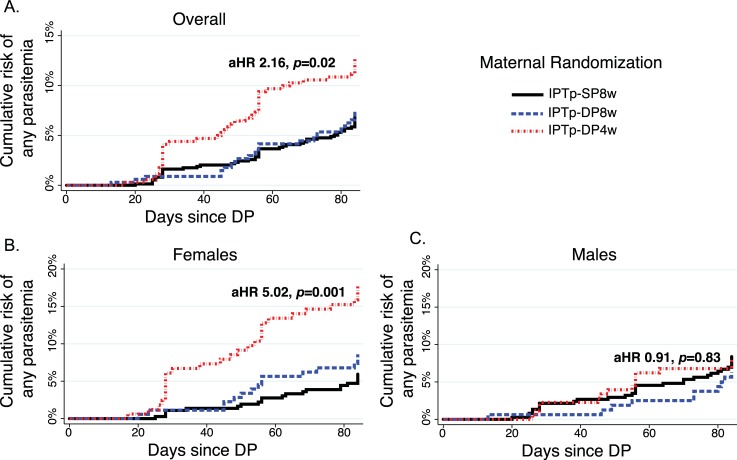
Time to parasitemia following DP administration in infancy. Repeated measures analysis performed, using multilevel mixed-effects survival model, account for clustering within individuals and mothers, both overall (A) and stratified by infant sex (B–C) and adjusted for maternal age, gravidity, and LAMP status at enrolment. *aHR and *p*-values comparing IPTp-DP4w versus IPTp-SP8w. aHR, adjusted hazard ratio; DP, dihydroartemisinin-piperaquine; IPTp, intermittent preventive treatment of malaria in pregnancy; IPTp-DP4w, IPTp-DP every 4 weeks; IPTp-DP8w, IPTp-DP every 8 weeks; IPTp-SP8w, IPTp-SP given every 8 weeks; LAMP, loop-mediated isothermal amplification.

After observing an apparently higher risk of clinical malaria among female infants whose mothers received IPTp-DP4w compared to those whose mothers received IPTp-SP8w, we next sought to determine whether these infants were at an increased risk of *P*. *falciparum* infection in infancy versus an increased risk of symptoms if infected. As children followed in this study received curative IPT with DP every 12 weeks beginning at 8 weeks of age, and had both passive and active surveillance for *P*. *falciparum* infection, we assessed time to parasitemia following each dose of DP in infancy. Overall, children born to mothers who received IPTp-DP4w had an increased hazard of parasitemia following receipt of DP in infancy compared to children born to mothers who received IPTp-SP8w (aHR 2.02, 95% CI 1.08–3.79, *p* = 0.03, Fig 2A, Table 4), but we again observed significant interaction with infant sex. Female infants whose mothers had received IPTp-DP4w had a more than 5-fold apparently increased hazard of developing parasitemia following receipt of DP in infancy than children born to mothers who received IPTp-SP8w (aHR, 5.02; 95% CI, 1.90–13.2, *p* = 0.001, Fig 2B, Table 4). There was no significant difference in the hazard of developing parasitemia among male children born to mothers who received IPTp-DP4w or IPTp-DP8w compared to male children born to mothers who received IPTp-SP8w (Table 4, Fig 2C). Prior to first receipt of DP at 8 weeks of age, parasitemia was rare: 1/182 (0.55%) were LAMP positive at week 4, and 3/183 (1.64%) were microscopy or LAMP positive at 8 weeks of age. All 4 children with parasitemia at or before 8 weeks of age were born to mothers who received IPTp-SP8w, and none of these episodes were symptomatic. These data suggest that the increased incidence of malaria among female infants whose mothers received IPTp-DP4w is directly related to an increased risk of blood stage *P*. *falciparum* infection following receipt of DP in infancy.

**Table 4:** Time to parasitemia following each dose of DP by mother’s IPTp treatment arm overall and stratified by infant sex.

Sex	Mother’s IPTp treatment arm	Number of doses of DP	Number with any parasitemia	Cumulative risk of any parasitemia (95% CI)	HR (95% CI)	*p*-value	aHR^a^ (95% CI)	*p*-value
All^b^	SP8w	742	52	7.1% (5.5%–9.3%)	reference		reference	
	DP8w	313	25	7.5% (5.1%–10.9%)	1.12 (0.56–2.24)	0.75	1.28 (0.64–2.60)	0.49
	DP4w	300	43	12.8% (9.6%–16.8%)	2.02 (1.08–3.79)	0.03	2.16 (1.14–4.11)	0.02
Female	SP8w	365	21	5.9% (3.9%–9.0%)	reference		reference	
	DP8w	178	15	8.6% (5.3%–13.9%)	1.61 (0.58–4.47)	0.36	2.28 (0.78–6.63)	0.13
	DP4w	165	29	17.9% (12.8%–24.7%)	4.18 (1.64–10.7)	0.003	5.02 (1.90–13.2)	0.001
Male	SP8w	377	31	8.4% (6.0%–11.7%)	reference		reference	
	DP8w	160	10	6.3% (3.5%–11.4%)	0.77 (0.31–1.92)	0.58	0.72 (0.28–1.80)	0.48
	DP4w	178	14	8.0% (4.8%–11.2%)	0.95 (0.41–2.20)	0.91	0.91 (0.39–2.10)	0.83

^a^Adjusted for maternal age, gravidity, LAMP status at enrolment, and maternal clustering for twin gestation.

^b^Interaction *p*-value between female sex and IPTp arm: 0.31 for ITPp-DP8w; 0.02 for IPTp-DP4w.

Abbreviations: aHR, adjusted hazard ratio; DP, dihydroartemisinin-piperaquine; DP4w, IPTp-DP every 4 weeks; DP8w, IPTp-DP given every 8 weeks; HR, hazard ratio; IPTp, intermittent preventive treatment of malaria in pregnancy; LAMP, loop-mediated isothermal amplification; SP8w, IPTp-SP given every 8 weeks.

### Secondary clinical and safety outcomes

During 2 years of follow-up, there were 9 episodes of complicated malaria: 4 among the 100 infants whose mothers received IPTp-SP8w (0.02 episodes PPY), 2 among 44 infants whose mothers received IPTp-DP8w (0.02 episodes PPY), and 3 among 47 infants whose mothers received IPTp-DP4w (0.04 episodes PPY). The risk of complicated malaria did not significantly differ between IPTp groups, nor between infant sex (5 episodes in males, 4 in females). Of the 9 episodes, 8 occurred between 12 and 24 months of age, 6 of 9 represented the child’s first episode of malaria and were associated with 1–2 convulsive episodes, and no episodes of cerebral malaria or severe anemia were observed. There were 3 deaths among infants included in this study. None of the deaths were due to malaria. Overall, there were 18 hospitalizations or deaths, with no significant difference in the number of hospitalization or deaths between IPTp treatment arms (Table 5). Children whose mothers received IPTp-DP4w or IPTp-DP8w had a nonsignificantly lower risk of anemia (hemoglobin <11 g/dL) compared to children whose mothers received IPTp-SP8w. Children born to mothers who received IPTp-DP4w or IPTp-DP8w also had a nonsignificantly lower incidence of non-malarial febrile illnesses compared to children born to mothers who received IPTp-SP8w (Table 5). These results are consistent with one other observational birth cohort, which found that malaria in pregnancy was associated with a higher risk of non-malarial febrile illness [42]. There were no significant differences in the incidence of adverse events of any severity, grade 3–4 adverse events, or serious adverse events (S1 Table). No adverse events were thought to be related to study drugs.

**Table 5:** Secondary outcomes by mother’s IPTp regimen.

Outcome	Sex	Mother’s IPTp treatment arm	Episodes or prevalence	PY of observation	Incidence PPY	IRR or OR (95% CI)	*p*-value	aIRR^a^ or aOR^a^ (95% CI)	*p*-value
Hospitalizations or deaths	All	SP8w	9	185.2	0.05	reference		reference	
		DP8w	2	84.2	0.02	0.49 (0.11–2.18)	0.35	0.52 (0.11–2.47)	0.41
		DP4w	7	85.5	0.08	1.69 (0.43–4.42)	0.29	1.76 (0.64–4.90)	0.28
Non-malarial febrile illnesses	All	SP8w	769	185.2	4.15	reference		reference	
		DP8w	302	84.2	3.59	0.85 (0.69–1.06)	0.16	0.86 (0.69–1.07)	0.17
		DP4w	292	85.5	3.42	0.82 (0.67–1.02)	0.07	0.82 (0.66–1.02)	0.08
Anemia^b^	All	SP8w	295/619 (47.7%)			reference		reference	
		DP8w	126/279 (45.2%)			0.85 (0.51–1.44)	0.55	0.91 (0.54–1.54)	0.73
		DP4w	119/285 (41.8%)			0.73 (0.44–1.22)	0.24	0.78 (0.46–1.29)	0.33

^a^Adjusted for maternal age, gravidity, LAMP status at enrolment, and maternal clustering for twin gestation.

^b^Hemoglobin <11 gm/dL at the time of routine visits conducted every 16 weeks.

Abbreviations: aIRR, adjusted incidence rate ratio; aOR, adjusted odds ratio (for anemia prevalence models); DP4w, IPTp-DP every 4 weeks; DP8w, IPTp-DP given every 8 weeks; IPTp, intermittent preventive treatment of malaria in pregnancy; IRR, incidence rate ratio; LAMP, loop-mediated isothermal amplification; OR, odds ratio; PY, person year; PPY, per person year; SP8w, IPTp-SP given every 8 weeks.

### Impact of IPTp with DP on antibody responses in infancy

**Fig 3:**
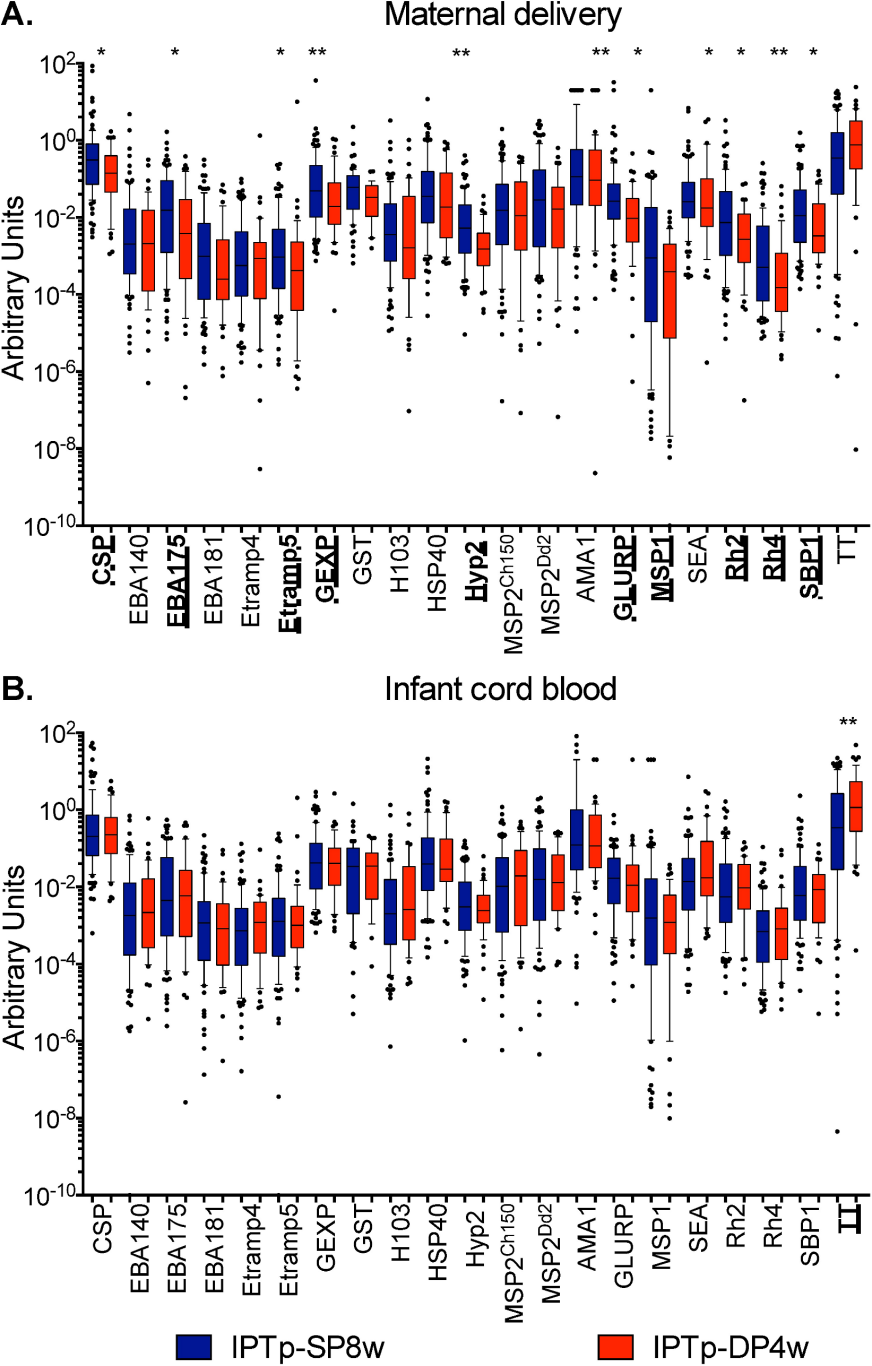
**Antibody levels stratified by maternal IPTp assignment (IPTp-SP8w versus IPTp-DP4w) measured in maternal blood at delivery (A) and infant cord blood (B).** *p < 0.05; **p < 0.01. AMA1, Apical membrane antigen 1; CSP, circumsporozoite protein; EBA, erythrocyte binding antigen; Etramp4, Early transcribed membrane protein 4; GEXP, gametocyte export protein; GST, Glutathione S-transferase; H103/MSP11, merozoite surface protein 11; HSP40, Heat shock protein 40; Hyp2, Plasmodium exported protein; IPTp, intermittent preventive treatment of malaria in pregnancy; IPTp-DP4w, IPTp-DP every 4 weeks; IPTp-SP8w; IPTp-SP every 8 weeks; MSP1, merozoite surface protein 1; MSP2^Ch150^ and MSP2^Dd2^, merozoite surface protein 2 of Ch150/90 and Dd2 alleles; Rh2, Reticulocyte-binding protein homologue 2; Rh4, Reticulocyte-binding protein homologue 4; SBP1, skeleton-binding protein 1; SEA, Schizont egress antigen; TT, tetanus toxoid.

Given the apparent sex-specific differences in malaria risk observed among infants whose mothers were randomized to different IPTp regimens, we then performed exploratory analyses to evaluate potential mechanistic explanations. As passive transfer of protective antibodies from the mother to the newborn may lead to a reduced risk of malaria early in life [37,43], we first assessed the impact of maternal IPTp regimens on malaria-specific IgG antibody levels in maternal and infant cord blood using a high-sensitivity Luminex-based, multiplex fluorescent bead-based assay. Although antibody levels to several antigens were significantly lower at delivery among mothers who received IPTp-DP4w in comparison to mothers who received IPTp-SP, IgG antibody levels measured in infant cord blood were similar between groups for all antigens (Fig 3). Furthermore, no significant sex-specific differences in antibody levels were observed. Therefore, the increased malaria risk observed among female infants whose mothers received IPTp-DP4w did not appear to be due to decreased malaria-specific antibody levels secondary to suppression of infection.

### Exposure to DP during pregnancy and PQ levels in infancy

**Fig 4:**
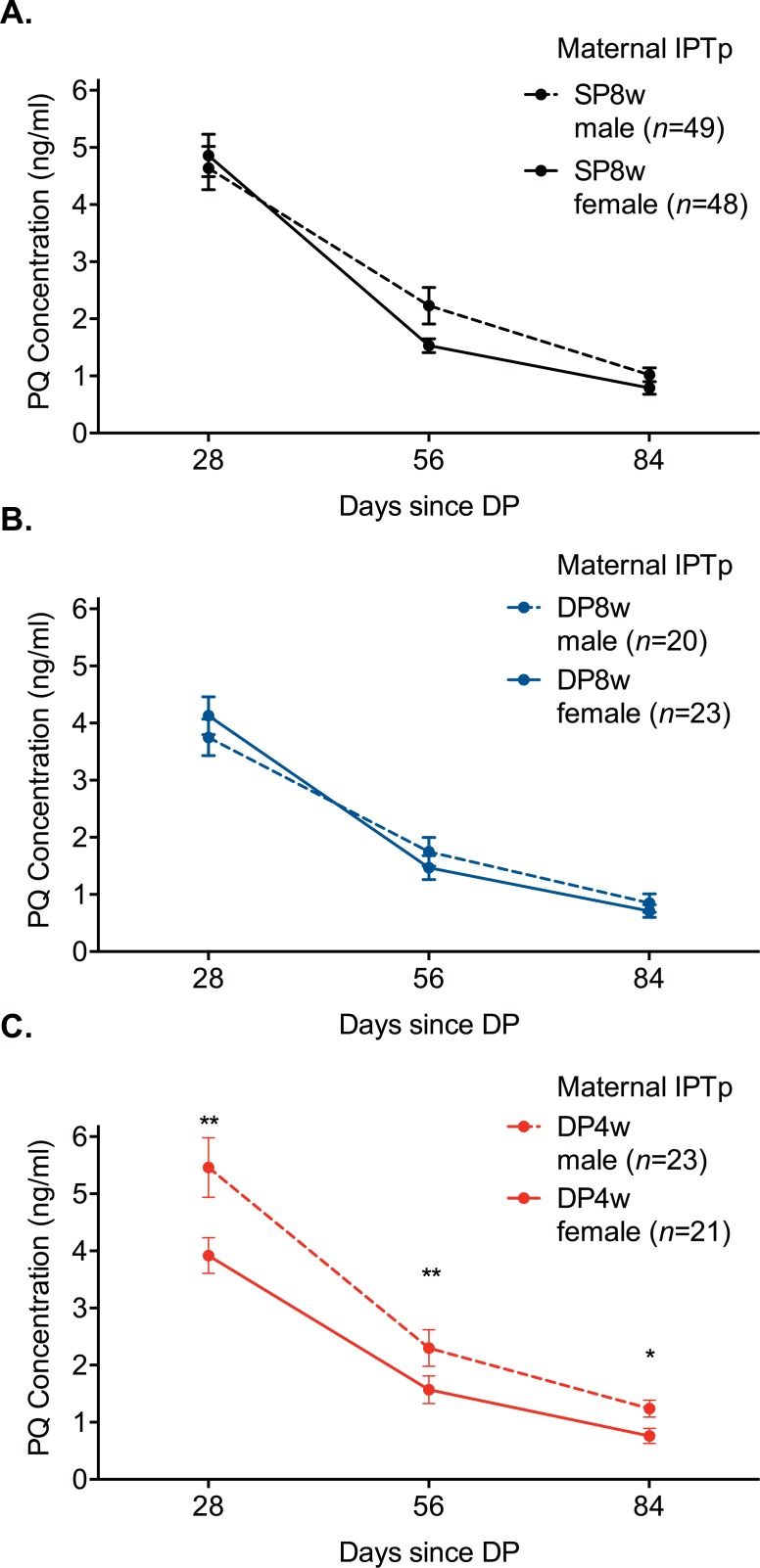
**Mean PQ levels measured 28, 56, and 84 days posttreatment among children whose mothers were randomized to IPTp-SP8w (A), IPTp-DP8w (B), and IPTp-DP4w (C).** Marginal estimates obtained using generalized estimating observations with log link and robust standard errors, accounting for repeated measurements in children. *p < 0.05. **p < 0.01. DP, dihydroartemisinin-piperaquine; IPTp, intermittent preventive treatment of malaria in pregnancy; DP4w, IPTp-DP given every 4 weeks; DP8w, IPTp-DP given every 8 weeks; SP8w, IPTp-SP given every 8 weeks; PQ, piperaquine; SP, sulfadoxine-pyrimethamine.

Because PQ trough levels are strongly predictive of malaria risk among children receiving IPT with DP [29], we next assessed whether there were sex-specific differences in PQ levels in infancy and whether in utero exposure to DP alters these PQ levels. Capillary PQ levels were measured 28, 56, and 84 days post the 8, 32, 56, and 92 week doses of DP in infancy (*n* = 1,505 measurements). Female children whose mothers were randomized to IPTp-DP4w had 19% lower mean PQ levels during infancy compared to female children whose mothers received IPTp-SP8w (coef 0.81, 95% CI 0.65–1.00, *p* = 0.048). In contrast, male children whose mothers were randomized to IPTp-DP4w had 17% higher mean PQ levels during infancy compared to males whose mothers received IPTp-SP8w, although not significantly so (coef 1.17, 95% CI 0.92–1.50, *p* = 0.21). In addition, female children whose mothers were randomized to IPTp-DP4w had 28% lower mean PQ levels post-dose than male children (coef 0.72, 95% CI 0.57–0.91, *p* = 0.006, Fig 4C). However, there were no significant sex-specific differences among children whose mothers were randomized to IPTp-SP8w (coef 1.07, 95% CI 0.86–1.32, *p* = 0.57, Fig 4A) or IPTp-DP8w (coef 1.09, 95% CI 0.97–1.38, *p* = 0.44, Fig 4B). Together, these data suggest that consistent in utero exposure to DP may lead to sex-specific differences in PQ metabolism in infancy, with lower PQ levels following administration among female infants.

## Discussion

In many parts of Africa, the current standard of care for IPTp—SP given at routine antenatal visits—remains inadequate to prevent malaria in pregnancy. DP has emerged as a promising alternative to IPTp-SP in settings with high SP resistance [7,28], but an important consideration is what impact IPTp-DP has on the risk of malaria during infancy. In contrast to our original hypothesis, we found that children born to mothers given IPTp-DP did not have a lower risk of malaria in infancy than children born to mothers given IPTp-SP. Rather, in a setting where children were given IPT with DP every 12 weeks during infancy, IPTp-DP4w was associated with a higher risk of malaria and *P*. *falciparum* infection in infancy—but only in female infants. Although levels of malaria-specific antibodies in cord blood did not significantly differ between IPTp groups and/or sex, female children whose mothers were randomized to IPTp-DP4w had significantly lower PQ levels during infancy compared to male children, suggesting that consistent in utero exposure to DP may lead to sex-specific differences in PQ metabolism in infancy.

Several epidemiologic and immunologic studies have previously suggested that in utero exposure to malaria may significantly increase a child’s subsequent risk of malaria [9–14], potentially by leading to immunologic tolerance of the parasite [11]. However, this has not been a universal observation, with some studies finding no significant association between in utero malaria exposure and subsequent malaria risk [16–18], and others reporting significant effect modification by maternal gravidity [14]. Importantly, as these were all observational studies, it is entirely possible that these associations are the result of confounding (i.e., mothers with malaria in pregnancy may live in foci of higher malaria transmission, and, therefore, their children are more likely to be exposed to infected mosquitoes in infancy) [19]. Although interventional studies following children born to mothers randomized to different IPTp regimens might more causally assess this question, few such RCTs have been performed. In the 4 studies, to our knowledge, in which pregnant women were randomized to different IPTp regimens and children followed during infancy, and exposure to infected mosquitoes can therefore be presumed to be equal between groups of children following birth, the incidence of malaria was not significantly different between groups [12,18,20,21]. However, in 3 of these 4 studies, the experimental group (IPTp-SP + LLIN, IPTp with mefloquine, or ISTp with AL) did not significantly reduce the burden of placental malaria in comparison to controls (LLIN alone or IPTp-SP for the latter 2 trials, respectively) [22–24]. In the older, placebo-controlled trial of IPTp with mefloquine in Thailand, mefloquine significantly reduced the burden of malaria in pregnancy in comparison to placebo. However, the child’s age at first malaria episode was assessed only through interview of the mother, and malaria blood smears were only performed at 1 week, 2 months, and 9 months of age [20].

In our placebo-controlled, randomized controlled study, IPTp-DP4w significantly reduced the burden of malaria infection during pregnancy and at delivery, offering an ideal opportunity to determine what impact reducing exposure to malaria in pregnancy might have on the risk of malaria in infancy. Children were followed through 2 years of age with both active and passive surveillance, allowing for precise determination of malaria metrics in infancy. Our study differed from the previously described studies above in that children born to mothers randomized to different IPTp interventions were themselves given IPT with DP every 12 weeks during infancy. In this setting, where the overall incidence of malaria in infancy was relatively low (0.3 episodes PPY), female infants whose mothers were randomized to IPTp-DP4w had an apparently 4-fold increased incidence of malaria and hazard of parasitemia following receipt of DP in infancy compared to female infants whose mothers were randomized to IPTp-SP8w. Female infants whose mothers were randomized to IPTp-DP8w also had nonsignificantly increased incidence of malaria and hazard of parasitemia. Importantly, no differences were observed among male children, adding to a growing literature suggesting sex-based differences in susceptibility to infectious diseases and vaccinations [44–47].

What might explain these sex-specific differences in malaria risk among children whose mothers were given different IPTp regimens? Several potential mechanisms for sex-based differences in susceptibility to infectious diseases have been described, including genetic differences attributable to X chromosome inactivation [48], sex-dependent differences in glucocorticoid receptor expression and fetal-placental responsivity to cortisol [49], and sex-specific differences in neonatal and infant immune responses [46,50]. In this study, we measured antibody levels to a broad array of antimalarial antigens in cord blood, given prior literature suggesting that passive transfer of protective antibodies from the mother to the newborn may mediate protection from malaria in the first 6–12 months of life [37,43,51]. Although we did not observe significant differences in antibody levels between IPTp groups or between females and males, our assay was limited to the antigens screened and did not include assessment of antibody functionality.

In our setting, an increased malaria risk among children was only observed among female infants whose mothers were randomized to IPTp with DP4w. Furthermore, we observed lower PQ concentrations among female infants whose mothers were randomized to IPTp with DP4w. Although pharmacokinetic studies of DP have not observed sex-specific differences in PQ levels to date, one possible explanation for these findings is that PQ exposure in utero or as a neonate may result in clinically important, sex-specific differences in PQ metabolism. PQ has been found in breast milk [52], and although transplacental transfer has not been documented for PQ, related aminoquinolones chloroquine [53] and mefloquine [54] are known to cross the placenta. PQ is metabolized by cytochrome P450 (CYP) 3A4/2C8 in adults [55], but enzymes responsible for PQ metabolism in infancy are not known. During the first 2 years of life, fetal CYP3A7 is gradually replaced by CYP3A4 [56], and potential explanations for sex-specific differences in PQ metabolism include sex-specific induction of fetal hepatic enzymes such as CYP3A7, which has been observed among female fetuses exposed to smoke [57], or accelerated maturation of adult CYP3A4, which may preferentially affect females who may have higher concentrations of CYP3A4 [58]. Further study will be needed to characterize the etiology of the lower PQ concentrations identified in female infants with mothers who received more frequent DP dosing.

Although we report findings from a randomized clinical trial, there were several important limitations. Our results suggesting an increased risk of malaria in female infants whose mothers were randomized to IPTp-DP must be interpreted with caution, as small sample size (especially when stratified by infant sex) may lend to spurious results. Furthermore, as children in this study all received DP every 12 weeks in infancy, we are unable to determine what impact maternal IPTp-DP has on infants who do not receive any IPT in infancy, which may be a more generalizable population across Africa. We are currently conducting a larger study comparing IPTp-DP with IPTp-SP among 782 mother-child pairs, in which children will be followed for 1 year following birth and not receive IPT in infancy. Finally, although suggestive of a potential mechanism of increased risk, our PQ data are speculative and require more formal pharmacokinetic/pharmacodynamic modelling, which is in progress.

In conclusion, our results show that effective prevention of in utero exposure to malaria with IPTp-DP does not result in a reduced risk of malaria in infants in comparison to IPT-SP. In contrast, in a setting where children were given IPT with DP every 12 weeks during infancy, IPTp-DP was associated with an increased risk of malaria in infancy, but only in female children. Future studies are needed to better understand the biological mechanisms of in utero drug exposure on drug metabolism and how this may affect the dosing of antimalarial drugs for treatment and prevention during infancy.

## Supporting information

S1 CONSORT Checklist (DOC)

S1 Study Protocol (DOC)

S1 Table Measures of safety and adverse events. (DOCX)

S1 Statistical Analysis Plan (DOCX)

S1 Analysis Timeline (XLSX)

## Abbreviations

aHR: adjusted hazard ratio
aIRR: adjusted incidence rate ratio
AL: artemether-lumefantrine
AMA1: Apical membrane antigen 1
AU: arbitrary unit
CBC: complete blood count
CSP: circumsporozoite protein
CYP: cytochrome P450
DBS: dried blood spot
DP: dihydroartemisinin-piperaquine
DP4w: IPTp-DP every 4 weeks
DP8w: IPTp-DP given every 8 weeks
EBA: erythrocyte binding antigen
Etramp: Early transcribed membrane protein
GEXP18: gametocyte exported protein
GLURP-R2: glutamate rich protein
GST: Glutathione S-transferase
HSP40: Heat shock protein 40
Hyp2: Plasmodium exported protein
IPT: intermittent preventive treatment
IPTp: intermittent preventive treatment of malaria in pregnancy
IPTp-DP: intermittent preventive treatment of malaria in pregnancy with dihydroartemisinin-piperaquine
IPTp-SP: intermittent preventive treatment of malaria in pregnancy with sulfadoxine-pyrimethamine
IRS: indoor residual spraying
IST: intermittent screening and treatment
LAMP: loop-mediated isothermal amplification
LBW: low birth weight
LLIN: long-lasting insecticidal net
LLOQ: lower limit of quantification
MSP: Merozoite surface protein
PPY: per person year
PQ: piperaquine
RCT: randomized controlled trial
Rh: Reticulocyte-binding protein homologue
RIII-V: region III-V
SBP1: skeleton-binding protein 1
SEA: Schizont egress antigen
SP: sulfadoxine-pyrimethamine
SP8w: IPTp-SP given every 8 weeks

## References

[1] WHO. World Malaria Report, 2015 Geneva, Switzerland: World Health Organization; 2015.

[2] Desai M, ter Kuile FO, Nosten F, McGready R, Asamoa K, Brabin B, et al Epidemiology and burden of malaria in pregnancy. Lancet Infect Dis. 2007;7(2):93–104. 10.1016/S1473-3099(07)70021-X . 17251080

[3] Gutman J, Kalilani L, Taylor S, Zhou Z, Wiegand RE, Thwai KL, et al The A581G Mutation in the Gene Encoding Plasmodium falciparum Dihydropteroate Synthetase Reduces the Effectiveness of Sulfadoxine-Pyrimethamine Preventive Therapy in Malawian Pregnant Women. J Infect Dis. 2015;211(12):1997–2005. 10.1093/infdis/jiu836 ; PubMed Central PMCID: PMC4539907. 25564249 PMC4539907

[4] Ranson H, N'Guessan R, Lines J, Moiroux N, Nkuni Z, Corbel V. Pyrethroid resistance in African anopheline mosquitoes: what are the implications for malaria control? Trends in parasitology. 2011;27(2):91–8. 10.1016/j.pt.2010.08.004 . 20843745

[5] A head-to-head comparison of four artemisinin-based combinations for treating uncomplicated malaria in African children: a randomized trial. PLoS Med. 2011;8(11):e1001119 Epub 2011/11/17. 10.1371/journal.pmed.1001119 ; PubMed Central PMCID: PMC3210754. 22087077 PMC3210754

[6] White NJ. Intermittent presumptive treatment for malaria. PLoS Med. 2005;2(1):e3 10.1371/journal.pmed.0020003 . 15696210 PMC545196

[7] Desai M, Gutman J, L'Lanziva A, Otieno K, Juma E, Kariuki S, et al Intermittent screening and treatment or intermittent preventive treatment with dihydroartemisinin-piperaquine versus intermittent preventive treatment with sulfadoxine-pyrimethamine for the control of malaria during pregnancy in western Kenya: an open-label, three-group, randomised controlled superiority trial. Lancet. 2015;386(10012):2507–19. 10.1016/S0140-6736(15)00310-4 ; PubMed Central PMCID: PMC4718402. 26429700 PMC4718402

[8] Kakuru A, Jagannathan P, Muhindo MK, Natureeba P, Awori P, Nakalembe M, et al Dihydroartemisinin-Piperaquine for the Prevention of Malaria in Pregnancy. N Engl J Med. 2016;374(10):928–39. 10.1056/NEJMoa1509150 . 26962728 PMC4847718

[9] Le Hesran JY, Cot M, Personne P, Fievet N, Dubois B, Beyeme M, et al Maternal placental infection with Plasmodium falciparum and malaria morbidity during the first 2 years of life. Am J Epidemiol. 1997;146(10):826–31. Epub 1997/12/31. . 9384203 10.1093/oxfordjournals.aje.a009200

[10] Schwarz NG, Adegnika AA, Breitling LP, Gabor J, Agnandji ST, Newman RD, et al Placental malaria increases malaria risk in the first 30 months of life. Clin Infect Dis. 2008;47(8):1017–25. Epub 2008/09/11. 10.1086/591968 . 18781874

[11] Malhotra I, Dent A, Mungai P, Wamachi A, Ouma JH, Narum DL, et al Can prenatal malaria exposure produce an immune tolerant phenotype? A prospective birth cohort study in Kenya. PLoS Med. 2009;6(7):e1000116 10.1371/journal.pmed.1000116 ; PubMed Central PMCID: PMC2707618. 19636353 PMC2707618

[12] Bardaji A, Sigauque B, Sanz S, Maixenchs M, Ordi J, Aponte JJ, et al Impact of malaria at the end of pregnancy on infant mortality and morbidity. J Infect Dis. 2011;203(5):691–9. 10.1093/infdis/jiq049 ; PubMed Central PMCID: PMC3071276. 21199881 PMC3071276

[13] Boudova S, Divala T, Mungwira R, Mawindo P, Tomoka T, Laufer MK. Placental but Not Peripheral Plasmodium falciparum Infection During Pregnancy Is Associated With Increased Risk of Malaria in Infancy. J Infect Dis. 2017;216(6):732–5. 10.1093/infdis/jix372 28934438 PMC5853669

[14] Mutabingwa TK, Bolla MC, Li JL, Domingo GJ, Li X, Fried M, et al Maternal malaria and gravidity interact to modify infant susceptibility to malaria. PLoS Med. 2005;2(12):e407 10.1371/journal.pmed.0020407 ; PubMed Central PMCID: PMC1277932. 16259531 PMC1277932

[15] Ndibazza J, Webb EL, Lule S, Mpairwe H, Akello M, Oduru G, et al Associations between maternal helminth and malaria infections in pregnancy and clinical malaria in the offspring: a birth cohort in entebbe, Uganda. J Infect Dis. 2013;208(12):2007–16. 10.1093/infdis/jit397 ; PubMed Central PMCID: PMC3836463. 23904293 PMC3836463

[16] Asante KP, Owusu-Agyei S, Cairns M, Dodoo D, Boamah EA, Gyasi R, et al Placental malaria and the risk of malaria in infants in a high malaria transmission area in ghana: a prospective cohort study. J Infect Dis. 2013;208(9):1504–13. 10.1093/infdis/jit366 ; PubMed Central PMCID: PMC3789576. 23908483 PMC3789576

[17] Apinjoh TO, Anchang-Kimbi JK, Mugri RN, Njua-Yafi C, Tata RB, Chi HF, et al Determinants of infant susceptibility to malaria during the first year of life in South Western cameroon. Open Forum Infect Dis. 2015;2(1):ofv012 10.1093/ofid/ofv012 ; PubMed Central PMCID: PMC4438893. 26034763 PMC4438893

[18] Awine T, Belko MM, Oduro AR, Oyakhirome S, Tagbor H, Chandramohan D, et al The risk of malaria in Ghanaian infants born to women managed in pregnancy with intermittent screening and treatment for malaria or intermittent preventive treatment with sulfadoxine/pyrimethamine. Malar J. 2016;15:46 10.1186/s12936-016-1094-z ; PubMed Central PMCID: PMC4730594. 26821532 PMC4730594

[19] Cairns M, Gosling R, Chandramohan D. Placental malaria increases malaria risk in the first 30 months of life: not causal. Clin Infect Dis. 2009;48(4):497–8; author reply 8–9. 10.1086/596548 . 19586381

[20] Nosten F, ter Kuile F, Maelankiri L, Chongsuphajaisiddhi T, Nopdonrattakoon L, Tangkitchot S, et al Mefloquine prophylaxis prevents malaria during pregnancy: a double-blind, placebo-controlled study. J Infect Dis. 1994;169(3):595–603. . 8158032 10.1093/infdis/169.3.595

[21] Ruperez M, Gonzalez R, Mombo-Ngoma G, Kabanywanyi AM, Sevene E, Ouedraogo S, et al Mortality, Morbidity, and Developmental Outcomes in Infants Born to Women Who Received Either Mefloquine or Sulfadoxine-Pyrimethamine as Intermittent Preventive Treatment of Malaria in Pregnancy: A Cohort Study. PLoS Med. 2016;13(2):e1001964 10.1371/journal.pmed.1001964 ; PubMed Central PMCID: PMC4764647. 26905278 PMC4764647

[22] Gonzalez R, Mombo-Ngoma G, Ouedraogo S, Kakolwa MA, Abdulla S, Accrombessi M, et al Intermittent preventive treatment of malaria in pregnancy with mefloquine in HIV-negative women: a multicentre randomized controlled trial. PLoS Med. 2014;11(9):e1001733 10.1371/journal.pmed.1001733 ; PubMed Central PMCID: PMC4172436. 25247709 PMC4172436

[23] Tagbor H, Cairns M, Bojang K, Coulibaly SO, Kayentao K, Williams J, et al A Non-Inferiority, Individually Randomized Trial of Intermittent Screening and Treatment versus Intermittent Preventive Treatment in the Control of Malaria in Pregnancy. PLoS ONE. 2015;10(8):e0132247 10.1371/journal.pone.0132247 ; PubMed Central PMCID: PMC4530893. 26258474 PMC4530893

[24] Menendez C, Bardaji A, Sigauque B, Romagosa C, Sanz S, Serra-Casas E, et al A randomized placebo-controlled trial of intermittent preventive treatment in pregnant women in the context of insecticide treated nets delivered through the antenatal clinic. PLoS ONE. 2008;3(4):e1934 10.1371/journal.pone.0001934 ; PubMed Central PMCID: PMC2277457. 18398460 PMC2277457

[25] Kamya MR, Arinaitwe E, Wanzira H, Katureebe A, Barusya C, Kigozi SP, et al Malaria transmission, infection, and disease at three sites with varied transmission intensity in Uganda: implications for malaria control. Am J Trop Med Hyg. 2015;92(5):903–12. 10.4269/ajtmh.14-0312 ; PubMed Central PMCID: PMC4426576. 25778501 PMC4426576

[26] Katureebe A, Zinszer K, Arinaitwe E, Rek J, Kakande E, Charland K, et al Measures of Malaria Burden after Long-Lasting Insecticidal Net Distribution and Indoor Residual Spraying at Three Sites in Uganda: A Prospective Observational Study. PLoS Med. 2016;13(11):e1002167 10.1371/journal.pmed.1002167 ; PubMed Central PMCID: PMC5100985. 27824885 PMC5100985

[27] Muhindo MK, Kakuru A, Natureeba P, Awori P, Olwoch P, Ategeka J, et al Reductions in malaria in pregnancy and adverse birth outcomes following indoor residual spraying of insecticide in Uganda. Malar J. 2016;15(1):437 10.1186/s12936-016-1489-x ; PubMed Central PMCID: PMC5002129. 27566109 PMC5002129

[28] Kakuru A, Jagannathan P, Muhindo MK, Natureeba P, Awori P, Nakalembe M, et al Dihydroartemisinin–Piperaquine for the Prevention of Malaria in Pregnancy. New England Journal of Medicine. 2016;374(10):928–39. 10.1056/NEJMoa1509150 26962728 PMC4847718

[29] Sundell K, Jagannathan P, Huang L, Bigira V, Kapisi J, Kakuru MM, et al Variable piperaquine exposure significantly impacts protective efficacy of monthly dihydroartemisinin-piperaquine for the prevention of malaria in Ugandan children. Malar J. 2015;14(1):368 10.1186/s12936-015-0908-8 ; PubMed Central PMCID: PMC4582734. 26403465 PMC4582734

[30] Division of AIDS (DAIDS) Table for Grading the Severity of Adult and Pediatric Adverse Events VW, D.C. US Department of Health and Human Services, National Institutes of Health, National Institute of Allergy and Infectious Diseases, Division of AIDS. 2014 [cited 2018 June 27]. Available from: https://rsc.tech-res.com/docs/default-source/safety/daids_ae_grading_table_v2_nov2014.pdf

[31] Beeson JG, Drew DR, Boyle MJ, Feng G, Fowkes FJ, Richards JS. Merozoite surface proteins in red blood cell invasion, immunity and vaccines against malaria. FEMS Microbiol Rev. 2016;40(3):343–72. 10.1093/femsre/fuw001 ; PubMed Central PMCID: PMC4852283. 26833236 PMC4852283

[32] Richards JS, Arumugam TU, Reiling L, Healer J, Hodder AN, Fowkes FJ, et al Identification and prioritization of merozoite antigens as targets of protective human immunity to Plasmodium falciparum malaria for vaccine and biomarker development. J Immunol. 2013;191(2):795–809. 10.4049/jimmunol.1300778 ; PubMed Central PMCID: PMC3702023. 23776179 PMC3702023

[33] Helb DA, Tetteh KK, Felgner PL, Skinner J, Hubbard A, Arinaitwe E, et al Novel serologic biomarkers provide accurate estimates of recent Plasmodium falciparum exposure for individuals and communities. Proc Natl Acad Sci U S A. 2015;112(32):E4438–47. Epub 2015/07/29. 10.1073/pnas.1501705112 ; PubMed Central PMCID: PMC4538641. 26216993 PMC4538641

[34] Polley SD, Conway DJ, Cavanagh DR, McBride JS, Lowe BS, Williams TN, et al High levels of serum antibodies to merozoite surface protein 2 of Plasmodium falciparum are associated with reduced risk of clinical malaria in coastal Kenya. Vaccine. 2006;24(19):4233–46. Epub 2005/08/23. 10.1016/j.vaccine.2005.06.030 . 16111789

[35] Collins CR, Withers-Martinez C, Bentley GA, Batchelor AH, Thomas AW, Blackman MJ. Fine mapping of an epitope recognized by an invasion-inhibitory monoclonal antibody on the malaria vaccine candidate apical membrane antigen 1. J Biol Chem. 2007;282(10):7431–41. Epub 2006/12/29. 10.1074/jbc.M610562200 . 17192270

[36] Theisen M, Vuust J, Gottschau A, Jepsen S, Hogh B. Antigenicity and immunogenicity of recombinant glutamate-rich protein of Plasmodium falciparum expressed in Escherichia coli. Clin Diagn Lab Immunol. 1995;2(1):30–4. Epub 1995/01/01. ; PubMed Central PMCID: PMC170096. 7719909 10.1128/cdli.2.1.30-34.1995 PMC170096

[37] Hogh B, Marbiah NT, Burghaus PA, Andersen PK. Relationship between maternally derived anti-Plasmodium falciparum antibodies and risk of infection and disease in infants living in an area of Liberia, west Africa, in which malaria is highly endemic. Infect Immun. 1995;63(10):4034–8. ; PubMed Central PMCID: PMC173567. 7558316 10.1128/iai.63.10.4034-4038.1995 PMC173567

[38] Raj DK, Nixon CP, Nixon CE, Dvorin JD, DiPetrillo CG, Pond-Tor S, et al Antibodies to PfSEA-1 block parasite egress from RBCs and protect against malaria infection. Science. 2014;344(6186):871–7. 10.1126/science.1254417 ; PubMed Central PMCID: PMC4184151. 24855263 PMC4184151

[39] Ambrosino E, Dumoulin C, Orlandi-Pradines E, Remoue F, Toure-Balde A, Tall A, et al A multiplex assay for the simultaneous detection of antibodies against 15 Plasmodium falciparum and Anopheles gambiae saliva antigens. Malar J. 2010;9:317 Epub 2010/11/10. 10.1186/1475-2875-9-317 ; PubMed Central PMCID: PMC2992071. 21059211 PMC2992071

[40] Keh CE, Jha AR, Nzarubara B, Lanar DE, Dutta S, Theisen M, et al Associations between antibodies to a panel of Plasmodium falciparum specific antigens and response to sub-optimal antimalarial therapy in Kampala, Uganda. PLoS ONE. 2012;7(12):e52571 10.1371/journal.pone.0052571 ; PubMed Central PMCID: PMC3526588. 23285095 PMC3526588

[41] Kjellin LL, Dorsey G, Rosenthal PJ, Aweeka F, Huang L. Determination of the antimalarial drug piperaquine in small volume pediatric plasma samples by LC-MS/MS. Bioanalysis. 2014;6(23):3081–9. 10.4155/bio.14.254 ; PubMed Central PMCID: PMC4321809. 25529877 PMC4321809

[42] Rachas A, Le Port A, Cottrell G, Guerra J, Choudat I, Bouscaillou J, et al Placental malaria is associated with increased risk of nonmalaria infection during the first 18 months of life in a Beninese population. Clin Infect Dis. 2012;55(5):672–8. 10.1093/cid/cis490 . 22610927

[43] Edozien JC, Gilles HM, Udeozo IOK. ADULT AND CORD-BLOOD GAMMA-GLOBULIN AND IMMUNITY TO MALARIA IN NIGERIANS. The Lancet. 280(7263):951–5. 10.1016/S0140-6736(62)90725-0

[44] Muenchhoff M, Goulder PJ. Sex differences in pediatric infectious diseases. J Infect Dis. 2014;209 Suppl 3:S120–6. 10.1093/infdis/jiu232 ; PubMed Central PMCID: PMC4072001. 24966192 PMC4072001

[45] Klein SL, Jedlicka A, Pekosz A. The Xs and Y of immune responses to viral vaccines. Lancet Infect Dis. 2010;10(5):338–49. 10.1016/S1473-3099(10)70049-9 . 20417416 PMC6467501

[46] Klein SL, Flanagan KL. Sex differences in immune responses. Nat Rev Immunol. 2016;16(10):626–38. 10.1038/nri.2016.90 . 27546235

[47] Klein SL, Shann F, Moss WJ, Benn CS, Aaby P. RTS,S Malaria Vaccine and Increased Mortality in Girls. MBio. 2016;7(2):e00514–16. 10.1128/mBio.00514-16 ; PubMed Central PMCID: PMC4850267. 27118593 PMC4850267

[48] Fish EN. The X-files in immunity: sex-based differences predispose immune responses. Nat Rev Immunol. 2008;8(9):737–44. 10.1038/nri2394 . 18728636 PMC7097214

[49] Clifton VL. Review: Sex and the human placenta: mediating differential strategies of fetal growth and survival. Placenta. 2010;31 Suppl:S33–9. Epub 2009/12/17. 10.1016/j.placenta.2009.11.010 . 20004469

[50] Prahl M, Jagannathan P, McIntyre TI, Auma A, Wamala S, Nalubega M, et al Sex Disparity in Cord Blood FoxP3+ CD4 T Regulatory Cells in Infants Exposed to Malaria In Utero. Open Forum Infect Dis. 2017;4(1):ofx022 Epub 2017/05/10. 10.1093/ofid/ofx022 ; PubMed Central PMCID: PMC5414097. 28480292 PMC5414097

[51] Murungi LM, Sonden K, Odera D, Oduor LB, Guleid F, Nkumama IN, et al Cord blood IgG and the risk of severe Plasmodium falciparum malaria in the first year of life. Int J Parasitol. 2017;47(2–3):153–62. 10.1016/j.ijpara.2016.09.005 ; PubMed Central PMCID: PMC5297353. 27890694 PMC5297353

[52] Moore BR, Salman S, Benjamin J, Page-Sharp M, Yadi G, Batty KT, et al Pharmacokinetics of piperaquine transfer into the breast milk of Melanesian mothers. Antimicrob Agents Chemother. 2015;59(7):4272–8. 10.1128/AAC.00327-15 ; PubMed Central PMCID: PMC4468664. 25963980 PMC4468664

[53] Law I, Ilett KF, Hackett LP, Page-Sharp M, Baiwog F, Gomorrai S, et al Transfer of chloroquine and desethylchloroquine across the placenta and into milk in Melanesian mothers. Br J Clin Pharmacol. 2008;65(5):674–9. 10.1111/j.1365-2125.2008.03111.x ; PubMed Central PMCID: PMC2432477. 18279478 PMC2432477

[54] Barzago MM, Omarini D, Bortolotti A, Stellari FF, Lucchini G, Efrati S, et al Mefloquine transfer during in vitro human placenta perfusion. J Pharmacol Exp Ther. 1994;269(1):28–31. . 8169835

[55] Lee TM, Huang L, Johnson MK, Lizak P, Kroetz D, Aweeka F, et al In vitro metabolism of piperaquine is primarily mediated by CYP3A4. Xenobiotica; the fate of foreign compounds in biological systems. 2012;42(11):1088–95. Epub 2012/06/08. 10.3109/00498254.2012.693972 . 22671777 PMC5087332

[56] Hines RN. The ontogeny of drug metabolism enzymes and implications for adverse drug events. Pharmacol Ther. 2008;118(2):250–67. 10.1016/j.pharmthera.2008.02.005 . 18406467

[57] O'Shaughnessy PJ, Monteiro A, Bhattacharya S, Fowler PA. Maternal smoking and fetal sex significantly affect metabolic enzyme expression in the human fetal liver. J Clin Endocrinol Metab. 2011;96(9):2851–60. 10.1210/jc.2011-1437 . 21715529

[58] Scandlyn MJ, Stuart EC, Rosengren RJ. Sex-specific differences in CYP450 isoforms in humans. Expert Opin Drug Metab Toxicol. 2008;4(4):413–24. . 18524030 10.1517/17425255.4.4.413

