# Supplementary material for: Dihydroartemisinin-piperaquine for intermittent preventive treatment of malaria during pregnancy and risk of malaria in early childhood: A randomized controlled trial
Source: PLoS Med. 2018 Jul 17;15(7):e1002606. doi: 10.1371/journal.pmed.1002606 (PMC6049882; doi:10.1371/journal.pmed.1002606)
Supplement: S1 Study Protocol — (DOC) [file pmed.1002606.s002.doc]

**“Novel Strategies to Prevent Malaria and Improve**

**Maternal-Child Health in Africa”**

**(promote II)**

**a ucsf/ makerere uNIVERSITy collaboration**

**Title: Reducing the Burden of Malaria in HIV-uninfected**

**Pregnant Women and Infants**

**Short Title: PROMOTE Birth Cohort 1**

**Version 6.0**

**Sponsored by:**

**The National Institute of Child Health and Human Development**

Principal Investigators: Grant Dorsey, MD, PhD

University California San Francisco

Moses Kamya, MBChB, MMed, PhD

Makerere University College of Health Sciences

Protocol Version 6.0 25^th^ October 2017

**TABLE OF CONTENTS**

[PROTOCOL TEAM ROSTER 6](#__RefHeading___Toc402430345)

[GLOSSARY 9](#__RefHeading___Toc402430346)

[SCHEMA 10](#__RefHeading___Toc402430347)

[1. INTRODUCTION 11](#__RefHeading___Toc402430348)

[**1.1.** **Background** 11](#__RefHeading___Toc402430349)

[**1.2.** **Preliminary studies** 14](#__RefHeading___Toc402430350)

[**Figure 1. Prevalence of key antifolate resistance-conferring mutations over time** 16](#__RefHeading___Toc402430351)

[**Figure 2. Cumulative risk of recurrent malaria following treatment with DP vs. AL** 17](#__RefHeading___Toc402430352)

[**Table 1. Protective efficacy against incident episodes of malaria** 18](#__RefHeading___Toc402430353)

[**Figure 3. Incidence of malaria over age stratified by assigned treatment arm** 18](#__RefHeading___Toc402430354)

[**Table 2. Incidence of malaria following cessation of chemoprevention** 19](#__RefHeading___Toc402430355)

[**1.3.** **Rationale** 19](#__RefHeading___Toc402430356)

[2. STUDY OBJECTIVES 20](#__RefHeading___Toc402430357)

[**2.1.** **Objective 1** 20](#__RefHeading___Toc402430358)

[**2.2.** **Objective 2** 21](#__RefHeading___Toc402430359)

[**2.3.** **Objective 3** 21](#__RefHeading___Toc402430360)

[3. STUDY DESIGN 21](#__RefHeading___Toc402430361)

[4. SELECTION AND ENROLLMENT OF SUBJECTS 22](#__RefHeading___Toc402430362)

[**4.1.** **Inclusion Criteria** 22](#__RefHeading___Toc402430363)

[**4.2.** **Exclusion Criteria** 22](#__RefHeading___Toc402430364)

[**4.3.** **Initial Screening** 22](#__RefHeading___Toc402430365)

[**4.4.** **Study Enrollment Procedures and Baseline Evaluation** 23](#__RefHeading___Toc402430366)

[5. STUDY TREATMENT 24](#__RefHeading___Toc402430367)

[**5.1.** **Treatment Group Assignments** 24](#__RefHeading___Toc402430368)

[**Table 3. Treatment arms with assignment of study drugs during pregnancy and infancy** 24](#__RefHeading___Toc402430369)

[**5.2.** **Treatment Allocation** 24](#__RefHeading___Toc402430370)

[**5.3.** **Study Drug Dosing and Formulations** 25](#__RefHeading___Toc402430371)

[**Table 5. Drug formulation and labeling** 25](#__RefHeading___Toc402430372)

[**5.4.** **Blinding, Study Drug Administration, and Duration** 25](#__RefHeading___Toc402430373)

[**5.5.** **Study Drug Accountability** 26](#__RefHeading___Toc402430374)

[6. SUBJECT MANAGEMENT 26](#__RefHeading___Toc402430375)

[**6.1.** **Subject Follow-up** 26](#__RefHeading___Toc402430376)

[**6.2.** **Diagnosis and Management of Malaria** 27](#__RefHeading___Toc402430377)

[**6.3.** **Management of Non-Malaria Illnesses** 28](#__RefHeading___Toc402430378)

[**6.4.** **After Hours Visits** 28](#__RefHeading___Toc402430379)

[**6.5.** **Routine Assessments** 29](#__RefHeading___Toc402430380)

[**6.6.** **Delivery visit** 30](#__RefHeading___Toc402430381)

[**6.7.** **Postpartum visits.** 30](#__RefHeading___Toc402430382)

[**6.8.** **Medical Care Outside the Study Clinic** 31](#__RefHeading___Toc402430383)

[**6.9.** **Duration of Follow-up and Criteria for Premature Study Withdrawal** 31](#__RefHeading___Toc402430384)

[**6.10.** **Diagnostic and Laboratory Testing** 32](#__RefHeading___Toc402430385)

[**6.10.1.** **Microscopy** 32](#__RefHeading___Toc402430386)

[**6.10.2.** **Clinical Laboratory Studies** 32](#__RefHeading___Toc402430387)

[**6.10.3.** **Placental Studies** 32](#__RefHeading___Toc402430388)

[**6.10.4.** **Molecular and Parasitology Studies** 33](#__RefHeading___Toc402430389)

[**6.10.5.** **Immunology Studies** 33](#__RefHeading___Toc402430390)

[**6.10.6.** **Pharmacokinetic (PK) Studies** 34](#__RefHeading___Toc402430391)

[**6.11.** **Co-enrollment Guidelines** 34](#__RefHeading___Toc402430392)

[6.12. Management of Adverse Events Potentially Related to Study Drugs 34](#__RefHeading___Toc402430393)

[**6.13.** **Grade 1 or 2 Adverse Events** 35](#__RefHeading___Toc402430394)

[**6.14.** **Grade 3 or 4 Adverse Events** 35](#__RefHeading___Toc402430395)

[7. MONITORING OF ADVERSE EVENTS AND MANAGEMENT 35](#__RefHeading___Toc402430396)

[**7.1.** **Monitoring and Reporting of Adverse Events** 35](#__RefHeading___Toc402430397)

[**7.1.1.** **Definitions** 36](#__RefHeading___Toc402430398)

[**7.1.2.** **Identification of Adverse Events** 36](#__RefHeading___Toc402430399)

[**7.1.3.** **Reporting of Adverse Events** 37](#__RefHeading___Toc402430400)

[**Table 6. Guidelines for reporting adverse events** 37](#__RefHeading___Toc402430401)

[8. STATISTICAL CONSIDERATIONS 38](#__RefHeading___Toc402430402)

[**8.1.** **Hypothesis 1** 38](#__RefHeading___Toc402430403)

[**8.1.1.** **Primary Outcome** 38](#__RefHeading___Toc402430404)

[**8.1.2.** **Secondary Outcomes** 38](#__RefHeading___Toc402430405)

[**Table 7. Secondary outcomes** 38](#__RefHeading___Toc402430406)

[**8.1.3.** **Analyses** 39](#__RefHeading___Toc402430407)

[**8.2.** **Hypothesis 2** 39](#__RefHeading___Toc402430408)

[**8.2.1.** **Primary Outcome** 40](#__RefHeading___Toc402430409)

[**8.2.2.** **Secondary Outcomes** 40](#__RefHeading___Toc402430410)

[**Table 8. Secondary outcomes** 40](#__RefHeading___Toc402430411)

[**8.2.3.** **Analyses** 40](#__RefHeading___Toc402430412)

[**8.3.** **Hypothesis 3** 41](#__RefHeading___Toc402430413)

[**8.3.1.** **Primary Outcome** 41](#__RefHeading___Toc402430414)

[**8.3.2.** **Secondary Outcomes** 41](#__RefHeading___Toc402430415)

[**8.3.3.** **Analyses** 42](#__RefHeading___Toc402430416)

[**8.4.** **Sample size and power** 42](#__RefHeading___Toc402430417)

[**Table 9. Intervention arms during pregnancy and infancy** 43](#__RefHeading___Toc402430418)

[**Table 10. Minimum relative differences in outcomes detectable given estimated effective sample sizes** 43](#__RefHeading___Toc402430419)

[**8.5.** **Data and Safety Monitoring Plan** 43](#__RefHeading___Toc402430420)

[**8.5.1.** **Data and Safety Monitoring Board** 44](#__RefHeading___Toc402430421)

[**8.5.2.** **Interim safety analysis** 45](#__RefHeading___Toc402430422)

[**Table 11. Schedule of interim safety analysis and boundaries to monitor study outcome** 46](#__RefHeading___Toc402430423)

[**8.5.3.** **Stopping rules** 46](#__RefHeading___Toc402430424)

[9. DATA COLLECTION AND MONITORING 46](#__RefHeading___Toc402430425)

[**9.1.** **Record Keeping** 46](#__RefHeading___Toc402430426)

[**9.2.** **Data Quality Assurance and Monitoring** 47](#__RefHeading___Toc402430427)

[10. HUMAN SUBJECTS 47](#__RefHeading___Toc402430428)

[**10.1.** **Subject Selection Criteria** 47](#__RefHeading___Toc402430429)

[**10.2.** **Risks and Discomforts** 47](#__RefHeading___Toc402430430)

[**10.2.1.** **Privacy** 47](#__RefHeading___Toc402430431)

[**10.2.2.** **Finger Pricks, Heel Sticks, and Venipuncture** 48](#__RefHeading___Toc402430432)

[**10.2.3.** **Risks of Study Medications** 48](#__RefHeading___Toc402430433)

[**Table 12. Summary of adverse events following treatment with dihydroartemisinin-piperaquine** 49](#__RefHeading___Toc402430434)

[**Table 14. Comparison of adverse events among children between 6-24 months of age** 52](#__RefHeading___Toc402430435)

[**10.3.** **Treatment and Compensation for Injury** 52](#__RefHeading___Toc402430436)

[**10.4.** **Costs to the Subjects** 52](#__RefHeading___Toc402430437)

[**10.5.** **Reimbursement of Subjects** 52](#__RefHeading___Toc402430438)

[**10.6.** **Institutional Review Board (IRB) Review and Informed Consent** 53](#__RefHeading___Toc402430439)

[**11.7** **Definition of Parent/Guardianship.** 53](#__RefHeading___Toc402430440)

[12. PUBLICATION OF RESEARCH FINDINGS 53](#__RefHeading___Toc402430441)

[13. BIOHAZARD CONTAINMENT 54](#__RefHeading___Toc402430442)

[14. REFERENCES 54](#__RefHeading___Toc402430443)

[15. APPENDICES 61](#__RefHeading___Toc402430444)

[**Appendix A. Information Sheet** 61](#__RefHeading___Toc402430445)

[Appendix B. Determination of Gestational Age 62](#__RefHeading___Toc402430446)

[**Appendix C. Household survey** 63](#__RefHeading___Toc402430447)

[**Appendix D. Administration of study drugs and placebos** 67](#__RefHeading___Toc402430448)

[**Appendix E. Weight-based dosing guidelines for DP during infancy** 68](#__RefHeading___Toc402430449)

[**Appendix F. WHO Criteria for Severe Malaria and Danger Signs** 69](#__RefHeading___Toc402430450)

[**Appendix G. Uganda Ministry of Health Guidelines for Routine Care of Pregnant and Postpartum Women, and Newborns** 70](#__RefHeading___Toc402430451)

[**Appendix H. Schedule of routine assessments and procedures in pregnant women** 71](#__RefHeading___Toc402430452)

[**Appendix I. Schedule of routine assessments and procedures in children** 73](#__RefHeading___Toc402430453)

[**Appendix J. DAIDS Toxicity Table (Dec 2004, clarification Aug 2009)** 74](#__RefHeading___Toc402430454)

[Adult Laboratory Toxicity Tables 87](#__RefHeading___Toc402430455)

[Pediatric Laboratory Toxicity Tables 88](#__RefHeading___Toc402430456)

[Appendix K. List of drugs associated with known risk of torsades de pointes 89](#__RefHeading___Toc402430457)

[Appendix L. List of drugs that potentially prohibit the metabolism of piperaquine 92](#__RefHeading___Toc402430458)

**PROTOCOL TEAM ROSTER**

**Investigators**

Grant Dorsey, MD, PhD University of California, San Francisco

Address: San Francisco General Hospital, 995 Potrero Avenue,

Building 30, Room 3240, UCSF Box 0811, San Francisco CA, 94110, U.S.A.

Phone Number: 01-415-206-4680

Fax Number: 01-415-648-8425

Email: gdorsey@medsfgh.ucsf.edu

Moses R. Kamya, MBChB, MMed, PhD, Makerere University College of Health Sciences

Address: Department of Medicine, P.O. Box 7072, Kampala, Uganda

Phone Number: +256-312281479

Fax Number: +256-414-540524

Email: [mkamya@infocom.co.ug](mailto:malaria@infocom.co.ug)

Diane V. Havlir, MD, University of California, San Francisco

Address: San Francisco General Hospital, 995 Potrero Avenue,

Building 80, Ward 84, UCSF Box 0874, San Francisco, CA 94110, U.S.A.

Phone Number: 01-415-476-4082, ext 400

Fax Number: 01-415-502-2992

Email: [dhavlir@php.ucsf.edu](mailto:dhavlir@php.ucsf.edu)

Miriam Nakalembe, MBChB, MMed Ob/Gyn, Makerere University College of Health Sciences

Address: Department of Medicine,

P.O. Box 7072, Kampala, Uganda

Phone Number: +256 753 857 433 (Mobile), +256 414 534 361 (Office)

Fax +256 414 533 451 (AOGU Office)

Email: ivuds@yahoo.com

Deborah Cohan, MD MPH, University California San Francisco

Address: San Francisco General Hospital, 995 Potrero Avenue

SFGH Box 6D22, San Francisco, CA. 94143 - SFGH

Phone Number: 01-415-206-3658

Fax Number: 01-415-206-3112

Email: cohand@obgyn.ucsf.edu

Edwin Charlebois, PhD, University of California, San Francisco

Address:  50 Beale St., 13^th^ Floor, San Francisco, CA 94105, U.S.A.

Phone Number: 01-415-597-9301

Fax Number: 01-415-597-9213

Email: [edwin.charlebois@ucsf.edu](mailto:echarlebois@psg.ucsf.edu)

Abel Kakuru, MBChB, MSc, Infectious Diseases Research Collaboration

Address: Infectious Diseases Research Collaboration,

P.O. Box 7475, Kampala, Uganda

Phone Number: +256-414-530692

Fax Number: +256-414-540524

Email: [abelkakuru@gmail.com](mailto:abelkakuru@gmail.com)

Maggie Feeney, MD, MSc, University of California, San Francisco

Address: UCSF, Parnassus Avenue Box 1234, San Francisco, CA 94143, U.S.A.

Phone Number: 415-206-8218

Email: [feeneym@medsfgh.ucsf.edu](mailto:feeneym@medsfgh.ucsf.edu)

Tamara Clark, MPH, University of California, San Francisco

            Address:  UCSF, Parnassus Avenue Box 0811, San Francisco, CA  94143, U.S.A.

Phone Number: 01-415-206-8790

Fax Number: 01-415-648-8425

            Email:   [tclark@medsfgh.ucsf.edu](mailto:gdorsey@medsfgh.ucsf.edu)

Theodore Ruel, MD, Assistant Professor, University of California, San Francisco, Dept Pediatrics

Address: UCSF, 500 Parnassus Avenue, MU4E, Campus Box 0136, San Francisco, CA 94143-0136

Phone Number: 01-415 476 9198

Fax Number: 01-415 476 1343

Email: ruelt@peds.ucsf.edu

Prasanna Jagannathan, MD, Assistant Professor, Stanford University, Division of Infectious Diseases and Geographic Medicine

Address: Lane Building, L133, 300 Pasteur Dr, Stanford, CA 94305

Phone Number: 01-650-724-5343

Email: [prasj@stanford.edu](https://mail.ucsf.edu/owa/redir.aspx?C=CMZMigpLgb2KqpqSgI24rRGEENNCHSyno6GnHSUehoQtYHMYRkDUCA..&URL=mailto%3Aprasj@stanford.edu)

OTHER PERSONNEL

| Name | Title | Institution |
| --- | --- | --- |
| Dr. Mary Kakuru | Study Coordinator | IDRC, Uganda |
| Dr. Paul Natureeba | Study Coordinator | IDRC, Uganda |
| Dr. Stephen Kinara | Study Coordinator | IDRC, Uganda |
| Peter Olwoch | Laboratory Manager | IDRC, Uganda |
| Bishop Opira | Study Pharmacist | IDRC, Uganda |
| Jaffer Okiring | Data Manager | IDRC, Uganda |
| Dr. Bridget Nzarubara | Regulatory Affairs Manager | IDRC, Uganda |
| Katherine Snyman | Quality Assurance Manager | UCSF |

**GLOSSARY**

AE Adverse event

ACT Artemisinin-based combination therapy

AL Artemether-lumefantrine

ALT Alanine transaminase (SGPT)

CAB Community advisory board

CBC Complete blood cell count

CRF Case report form

DP Dihydroartemisinin-piperaquine

DSMB Data and Safety Monitoring Board

IDRC Infectious Diseases Research Collaboration

IPTp Intermittent preventive therapy in pregnancy

IRB Institutional review board

ITN Insecticide treated net

MOH Ministry of Health

MU Makerere University

NICHD National Institute of Child Health and Human Development

NIH National Institute of Health

SAE Serious adverse event

SP Sulfadoxine-pyrimethamine

TDH Tororo District Hospital

UCSF University California San Francisco

WHO World Health Organization

**SCHEMA**

| **Title** | Reducing the Burden of Malaria in HIV-uninfected Pregnant Women and Infants | |
| --- | --- | --- |
| **Description** | Double blinded randomized controlled trial | |
| **Study Objectives** | 1. To compare the risk of placental malaria among HIV-uninfected pregnant women randomized to receive IPTp with 3 dose SP vs. 3 dose DP vs. monthly DP. 2. To compare the incidence of malaria among infants randomized to receive q 3 monthly DP vs. monthly DP between 2-24 months of age. 3. To compare the incidence of malaria among infants and children following the two phases of the intervention through 36 months of age | |
| **Participants and Sample Size** | 300 HIV-uninfected pregnant women and the children born to them | |
| **Clinical Site** | The study will be conducted in Tororo, Uganda. A designated study clinic will be located within the Tororo District Hospital Complex. The study clinic will be open daily from 8:00 am to 5:00 pm and after-hours care will be available at Tororo District Hospital. | |
| **Selection Criteria** | Inclusion Criteria   1. Pregnancy confirmed by positive urine pregnancy test or intrauterine pregnancy by ultrasound 2. Estimated gestational age between 12-20 weeks 3. Confirmed to be HIV- uninfected by rapid test 4. 16 years of age or older 5. Residency within 30km of the study clinic 6. Provision of informed consent 7. Agreement to come to the study clinic for any febrile episode or other illness and avoid medications given outside the study protocol 8. Plan to deliver in the hospital   Exclusion Criteria:   1. History of serious adverse event to SP or DP 2. Active medical problem requiring inpatient evaluation at the time of screening 3. Intention of moving more than 30km from the study clinic 4. Chronic medical condition requiring frequent medical attention 5. Prior SP preventive therapy or any other antimalarial therapy during this pregnancy 6. Early or active labor (documented by cervical change with uterine contractions) | |
| **Treatment assignment** | HIV-uninfected pregnant and their unborn child(ren) will be randomized at the time of enrollment. | |
| **Treatment arms** | IPTp during pregnancy | Chemoprevention in children 2-24 months of age |
|  | 1. 3 dose SP 2. 3 dose DP 3. Monthly DP | 1. Monthly DP 2. 3 monthly DP |
| **Follow-up and Diagnosis of Malaria** | Study participants will be followed for all of their outpatient medical care in our study clinic. Pregnant women will be followed until 6 weeks postpartum and children will be followed until 36 months of age. Routine assessments will be performed in the study clinic for all study participants every 4 weeks. Patients presenting with a new episode of fever will undergo standard evaluation for the diagnosis of malaria. | |
| **Malaria Case Definitions** | **Uncomplicated malaria (all of the following):**  1. Documented fever or history of fever in the prior 24 hours  2. Positive thick blood smear  3. Absence of complicated malaria  **Complicated malaria (any of the following):**  1. Evidence of severe disease with a positive thick blood smear (Appendix F)  2. Danger signs in children with a positive thick blood smear (Appendix F) | |

1. **INTRODUCTION**
   1. **Background**

**Malaria in Pregnancy: burden of disease and prevention.** In sub-Saharan Africa approximately 25 million pregnant women are at risk of *P. falciparum* infection every year and 25% have evidence of placental infection.[^1^](#_ENREF_1) Among pregnant women living in areas of stable transmission few infections lead to symptomatic malaria, however, infection is associated with maternal morbidity, such as anemia, and adverse birth outcomes including abortions, stillbirth, preterm delivery, low birth weight (LBW), and infant mortality.

In Africa the only widely available tools for the prevention of malaria in pregnancy are ITNs and IPTp with SP. Older studies demonstrated the efficacy of IPTp with SP in reducing the risk of placental malaria and LBW.[^4-8^](#_ENREF_4) However, there are now concerns about the continued efficacy of IPTp given the spread of antifolate resistance. A 2007 review suggested that IPTp with SP remained beneficial in areas with antifolate resistance, however, this conclusion was based on communities where SP treatment failure rates in children remained at moderate levels.[^9^](#_ENREF_9) More recently, reports from East Africa have documented SP failure rates over 65% in children when used for treatment[^10^](#_ENREF_10) or prevention[^11^](#_ENREF_11), and near saturation of common SP resistance alleles. In a recent study from Tanzania IPTp with SP was not associated with a decreased risk of placental malaria, maternal anemia, or LBW, and unexpectedly associated with an increased risk of fetal anemia.[^14^](#_ENREF_14) In a recent study from Uganda there was no significant difference in the risk of maternal infection, maternal anemia, and LBW for pregnant women receiving IPTp with SP plus ITNs vs. ITNs alone.[^15^](#_ENREF_15) In addition, most IPTp studies have defined placental malaria on the basis of placental blood smears, which dramatically underestimate the true prevalence of placental malaria. In summary, there are several lines of evidence suggesting that IPTp with SP is no longer effective in areas of East Africa with widespread antifolate resistance. New interventions to reduce the burden of malaria in pregnancy in this region are desperately needed.

The ACT class of drugs offers an attractive alternative to SP for use in pregnancy. In a recent systematic review of parasitological efficacy for the treatment and prevention of falciparum malaria in pregnancy, placenta-positive rates were unacceptably high in a majority of SP trial arms and ACTs provided the lowest parasitological failure rates.[^16^](#_ENREF_16) The authors recommended that SP should no longer be used for treatment or prevention of malaria in pregnancy and that ACTs provide the most efficacious and safe alternative therapy. Two studies of the ACT artesunate (AS) + SP from Africa concluded that this drug was safe for the treatment of malaria in pregnant women. More recent studies have focused on artemether-lumefantrine (AL), considering efficacy, pharmacokinetics, and safety. In a prospective study from Zambia, 495 pregnant women exposed to AL (including 156 in the 1^st^ trimester) had similar risks of adverse maternal and infant outcomes compared to pregnant women exposed to SP.[^19^](#_ENREF_19) In a recent study from Uganda, pregnant women in their 2^nd^ or 3^rd^ trimester with peripheral parasitemia treated with AL had a cure rate of 99%.[^20^](#_ENREF_20) DP has also been safely used for the treatment of uncomplicated malaria in pregnancy in studies from Asia. In two recent studies on the treatment of uncomplicated malaria with DP in pregnant and non-pregnant women, one concluded that there were no clinically important differences in piperaquine pharmacokinetics in pregnancy [^23^](#_ENREF_23) and another concluded that pregnancy was associated with an unaltered total exposure to piperaquine but a shorter terminal elimination half-life.[^24^](#_ENREF_24)

In summary, most African countries continue to recommend IPTp with SP, however, there are serious concerns about the efficacy of SP given widespread resistance, especially in East Africa. Available data have shown that ACTs are effective and safe for the treatment of malaria in pregnancy and are now recommended by the WHO as 1^st^ line therapy for pregnant women in their 2^nd^ or 3^rd^ trimester.[^25^](#_ENREF_25) However, there are no published studies evaluating the safety and efficacy of ACTs for use as preventive therapy in pregnant women.

**Malaria in Infants and Young Children: burden of disease and prevention.** Partial immunity to malaria develops through repeated exposure, leading first to protection against severe forms of disease, followed by protection against symptomatic illness.[^26^](#_ENREF_26) Thus, the burden of malaria in Africa is heavily borne by young children. Newborns are protected during the first few months of life, likely due to the transplacental acquisition of maternal antibodies and relatively high fetal hemoglobin content.[^27^](#_ENREF_27) After about 2-3 months of age, protection from these factors wanes.[^28^](#_ENREF_28) However, the age at which malaria risk peaks in endemic areas of Africa varies from 1-2 years of age in areas of high transmission intensity to approximately 5 years of age in areas of low to moderate transmission intensity.[^29^](#_ENREF_29) When studying malaria incidence and interventions to prevent malaria, it is essential to have a thorough understanding of the local epidemiology of the disease. Our proposed studies will benefit from the extensive experience we have gained studying malaria in Uganda.

The only widely used tool for the prevention of malaria in African children is ITNs, however, there is concern for diminishing efficacy of ITNs due to the alarming emergence of vector resistance to pyrethroids (currently the only class of insecticides used in ITNs) in Uganda and other parts of Africa. Our collaboration has been following a cohort of 350 children since 2007 in Tororo, the site of our proposed trial. To date, children between 6-24 months of age have suffered over 5 episodes of malaria per year, and between 2008-11 the incidence of malaria increased by over 50%, despite the use of ITNs.[^34^](#_ENREF_34) Extending the use of IPT to African infants and children at high risk for malaria offers a potential new preventive tool that has recently received widespread attention. The intervention that has been most extensively studied is SP given at the time of routine vaccination in infants (termed IPTi). A recent pooled analysis of 6 RCTs reported that IPTi was safe and associated with a modest 30% protective efficacy against clinical malaria in the first year of life.[^35-40^](#_ENREF_35) In a study from Kenya, IPTi with SP + AS or amodiaquine (AQ) + artesunate was associated with a 22% and 25% protective efficacy against malaria, respectively.[^41^](#_ENREF_41) A different approach to IPT has been taken in parts of West Africa, where the main burden of malaria is in older children and transmission is highly seasonal. In this setting, studies of IPT (termed seasonal malaria chemoprevention or SMC) have delivered drugs at monthly intervals during the transmission season, targeting children under 5 years of age. In two such studies using SP+AQ, the protective efficacy ranged from 70-82%. In a systematic review and meta-analysis of 12 studies, monthly SMC was safe , with an overall protective efficacy of 82% against clinical malaria and 57% against all-cause mortality.[^44^](#_ENREF_44) SMC has now been recommended as a new malaria control strategy following a meeting of the Malaria Policy Advisory Committee to the WHO in early 2012.[^45^](#_ENREF_45) However, this recommendation only pertains to areas with seasonal malaria transmission, and so not to the vast parts of Africa, such as Uganda, with year-round transmission. In addition, good efficacy for SMC with SP+AQ has been documented in regions of West Africa with considerably lower prevalence of resistance to both SP and AQ than is the case in many other areas, including Uganda.

In summary, IPT offers great promise for reducing the burden of malaria in African infants and children, however, interventions must be carefully chosen based on drug resistance patterns and the local epidemiology of disease. IPTi with SP has been associated with modest protective efficacy, however, the WHO recommends IPTi only in countries with moderate to high malaria transmission, where parasite resistance to SP is low.[^46^](#_ENREF_46) SMC using monthly dosing with primarily SP containing combination therapies has been highly efficacious in areas of West Africa where malaria transmission is seasonal, however, this strategy would not be appropriate in most areas of Central and East Africa were transmission is perennial and SP resistance widespread. DP is a new co-formulated ACT that has been suggested as an excellent candidate for IPT in infants and children.[^27^](#_ENREF_27) Several studies from Africa have demonstrated that DP is safe and highly efficacious for the treatment of uncomplicated malaria and has the added benefit over other ACTs in terms of prolonged post-treatment prophylaxis.[^47-50^](#_ENREF_47) DP is now one of the WHO recommended 1st line treatments for malaria and was approved for use in Uganda in 2005. In the only published randomized, placebo-controlled trial of DP for the prevention of malaria conducted in healthy adult Thai males, the protective efficacy of DP over a 9 month period was 98% when the drug was given monthly and 86% when the drug was given every 2 months. [^51^](#_ENREF_51) In an ongoing study carried out by our group, monthly DP given to infants between the ages of 6-24 months has been highly efficacious for the prevention of malaria in a high transmission setting of Uganda (see section 1.2 preliminary studies).

**Impact of Malaria Prevention on the Development of Immunity.** Increasing evidence suggests that maternal infection during pregnancy affects the developing immune system of fetuses independent of potential vertical transmission of pathogens.[^52^](#_ENREF_52) Several studies indicate that placental malaria is associated with altered parasite-specific immune responses in neonates that could affect response to malaria after birth.[^53-56^](#_ENREF_53) In addition, several clinical studies have reported that infants born to mothers with placental malaria have a higher risk of death [^57^](#_ENREF_57) and malaria during infancy.[^58-61^](#_ENREF_58) However, all of these were observational studies in which it is very difficult to control for exposure, which is tightly linked between pregnant women and their infants. It remains unclear whether, as we hypothesize in this proposal, prevention of malaria during pregnancy improves the development of antimalarial immunity and reduces the risk of malaria after birth.

There is conflicting evidence on the impact of chemoprevention in children on the development of antimalarial immunity and the risk of malaria after chemoprevention has stopped. Studies from Tanzania and the Gambia reported that children receiving highly effective chemoprophylaxis had a higher incidence of malaria compared to those receiving placebo in the year following the intervention. In contrast, more recent studies have reported no association between IPTi with SP and the risk of malaria following the intervention. Differences in these findings could be due to differences in transmission intensity, ages of study subjects, protective efficacy of study drugs, and dosing strategies (continuous prophylaxis vs. intermittent therapy). Recent unpublished data from our group suggest that in a high transmission setting, chemoprevention may actually improve the development of antimalarial immunity. Children randomized to daily trimethoprim-sulfamethoxazole (TS) had a 39% reduction in the incidence of malaria during the time the intervention was given [^13^](#_ENREF_13) and a 23% reduction in the incidence of malaria in the 1 year after the intervention was stopped, although this did not reach statistical significance (p=0.12). This may be due to improved priming of cellular immune responses to pre-erythrocytic stage antigens, as has been observed in several animal and experimental models where parasitemia is suppressed with drugs that are active only against erythrocytic stages.[^64-68^](#_ENREF_64)

- 1. **Preliminary studies**

**Intermittent preventive therapy in pregnancy with sulfadoxine-pyrimethamine.** We conducted a cross-sectional study of 565 HIV-uninfected women giving birth between February – July 2011 at Tororo District Hospital (manuscript currently in press). The primary objective of the study was to measure associations between use of SP during pregnancy from antenatal records and the risk of adverse outcomes including placental malaria, low birth weight, maternal parasitemia and maternal anemia. The proportion of women who reported taking 0, 1, 2, and 3 doses of SP during pregnancy was 5.7%, 35.8%, 56.6% and 2.0% respectively. Overall, the prevalence of placental malaria was 17.5%, 28.1%, and 66.2% by placental smear, PCR, and histopathology, respectively. In multivariate analyses controlling for potential confounders, > 2 doses of SP was associated with non-significant trends towards lower odds of placental malaria by placental smear (OR=0.75, p=0.25), placental malaria by PCR (OR=0.93, p=0.71), placental malaria by histopathology (OR=0.75, p=0.16), low birth weight (OR=0.63, p=0.11), maternal parasitemia (OR=0.88, p=0.60) and maternal anemia (OR=0.88, p=0.48). Using a composite outcome, > 2doses of SP was associated with a significantly lower odds of placental malaria, low birth weight, maternal parasitemia, or maternal anemia (OR=0.52, p=0.01). In this area with intense malaria transmission, the prevalence of placental malaria by histopathology was high even among women who reported taking at least 2 doses of SP during pregnancy. The reported use of > 2 doses of SP was not associated with protection against individual birth and maternal outcome.

**Antifolate Resistance in Uganda.** Surveillance of key mutations in *P. falciparum dhfr* and *dhps* genes, which encode the target enzymes of SP and TS, has been proposed as a means of monitoring antifolate drug resistance in Africa. We have studied the association between the five key mutations commonly reported in Africa and clinical treatment failure in children treated with SP for uncomplicated malaria in Kampala . The prevalences of the *dhfr* 108N (98%) and 51I (95%) mutations were very high, and therefore these mutations were not useful independent predictors of treatment outcome. Considering combinations of mutations, there was generally a “dose response” relationship, with an increasing number of mutations resulting in stronger associations with treatment failure. Infections with parasites containing the quintuple mutant (*dhfr* 108N + 51I +59R; *dhps* 437G + 540E) was associated with over 10 times the odds of treatment failure compared to infections with parasites containing only the 108N and 51I mutations (OR = 10.7, 95%CI 1.8-64.4, p = 0.009).

We have measured the prevalence of key antifolate resistance-conferring mutations from subjects living in Tororo (the site of this proposal) over a 4 year period. From 2002-2006 the prevalence of the *dhfr* triple mutant increased from 40% to almost 80% and the prevalence of the *dhps* triple mutant increased from 60% to almost 100% (Figure 1). This temporal increase in the prevalence of molecular markers of antifolate resistance corresponds to the 2001 implementation of a national policy change from CQ to CQ+SP as the recommended first-line treatment for malaria in Uganda [^71^](#_ENREF_71). We have also measured the prevalence of key antifolate resistance-conferring mutations from the 9 children in a cohort of HIV-infected children who developed symptomatic malaria while taking TS prophylaxis [^72^](#_ENREF_72). All of these samples contained the *dhfr/dhps* quintuple mutant and one sample contained an additional mutation (*dhfr* 164L) associated with high-level antifolate resistance (probably leading to complete loss of antimalarial activity of antifolates) that is rare in Africa [^73^](#_ENREF_73) and was only very rarely detected in hundreds of prior samples from Uganda evaluated by our group. These data provided further evidence that SP, currently the regimen used for IPTp in Uganda, may be faced with diminishing protective efficacy. Thus, alternative regimens and strategies, such as DP as proposed in our protocol, should be considered.

**Figure 1. Prevalence of key antifolate resistance-conferring mutations over time**

**Clinical trials of DP.** DP is a new co-formulated ACT that was approved for use in Uganda in 2005. We have completed two standard clinical trials in Uganda comparing DP to AL, . These trials were conducted at sites with both low (Kanungu) and high (Apac) malaria transmission intensity, enrolling 835 patients, including 741 children under the age of 5 years. Both drugs were highly efficacious, with a low risk of recrudescence after therapy. Relevant to this proposal is the drugs’ post-treatment prophylactic effect up to one month after therapy. One month after therapy, risks of recurrent parasitemia with DP were 4% and 11% at the low and high transmission sites, respectively, compared to risks of 18% and 29% with AL (p<0.0001 for both comparisons). In addition, DP was better tolerated than AL at both sites, with no serious adverse events related to DP.

More recently we completed a longitudinal clinical trial comparing DP to AL in a cohort of young children in Tororo (manuscript submitted for publication). Overall, 312 children were randomized to DP or AL at the time of the first episode of uncomplicated malaria (median age 10.5 months). The same treatment was given for all subsequent episodes of uncomplicated malaria and children were followed until they reached 5 years of age. The cohort included a subgroup that was HIV-infected or HIV-exposed prescribed TS prophylaxis. Outcomes included time to recurrent malaria following individual treatments and the overall incidences of treatments for malaria, complicated malaria, and hospitalizations. Among children not prescribed TS prophylaxis, 4443 treatments for malaria were given over 790 person years following randomization. Treatment with DP was associated with a less than 5% risk of recurrent malaria within 28 days and a lower hazard of recurrent malaria over 84 days compared to AL (HR=0.66, 95% CI 0.61-0.70, *P*<0.001)(Figure 2). Children randomized to DP also had a lower incidence of all treatments for malaria (IRR=0.85, 95% CI 0.75-0.96, *P*=0.01), complicated malaria (IRR=0.12, 95% CI 0.04-0.39, *P*<0.001), and hospitalizations (IRR=0.31, 95% CI 0.13-0.77, *P*=0.01). In addition, repeated treatments with DP were safe, with a low risk of adverse events that were comparable to AL.[^75^](#_ENREF_75)

### **Figure 2. Cumulative risk of recurrent malaria following treatment with DP vs. AL**

**
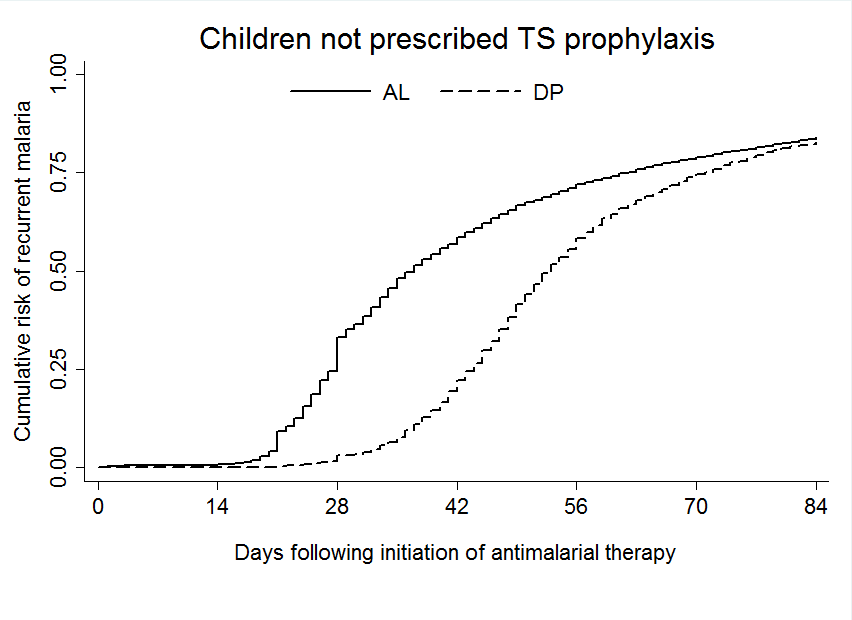
**

**Clinical trial of chemoprevention in young Ugandan children living in Tororo.** Chemoprevention offers a promising strategy for the prevention of malaria in African children. However, the optimal chemoprevention drug and dosing strategy is unclear in areas of year-round transmission and high prevalence of resistance to many antimalarial drugs. We recently completed an open-label, randomized controlled trial of antimalarial chemoprevention in young children living in Tororo, an area of intense, year-round transmission in Eastern Uganda. A total of HIV-unexposed 400 infants were enrolled between 4-5 months of age and 393 randomized to one of four treatment arms at 6 months of age: no chemoprevention, monthly SP, daily TS, or monthly DP. Study drugs were administered at home without supervision. Participants were given a long-lasting insecticide treated bednet at enrollment and followed for all their health care needs. At 24 months of age the intervention was stopped and study participants were then followed to 36 months of age to compare the incidence of malaria over the 1 year period after chemoprevention was stopped. During the intervention, the incidence of malaria in the no chemoprevention group was 6.95 episodes per person-year. Monthly DP had a protective efficacy of 58% (95% CI 45-67%, p<0.001) against malaria and 47% (95% CI 1-72%, p=0.04) against moderate-severe anemia. Daily TS had a protective efficacy of 28% (95% CI 7-44%, p=0.01) against malaria but offered no protection against moderate-severe anemia (-21%, 95% CI -107 to 29%, p=0.49). Monthly SP offered no protection against malaria (7%, 95% CI, -19 to 28%, p=0.57) and had an excess risk of moderate-severe anemia (-70%, 95% CI -184 to -2%, p=0·04)(Table 1).

### **Table 1. Protective efficacy against incident episodes of malaria**

| **Treatment arm** | **6-24 months of age** | | | | |
| --- | --- | --- | --- | --- | --- |
|  | **Number of cases** | **PYAR** | **Incidence per PYAR** | **PE (95% CI)** | **p value** |
| Control | 760 | 109.3 | 6.95 | reference | - |
| Monthly SP | 725 | 107.8 | 6.73 | 7% (-19 to 28) | 0.57 |
| Daily TS | 609 | 116.8 | 5.21 | 28% (7 to 44) | 0.01 |
| Monthly DP | 366 | 121.3 | 3.02 | 58% (45 to 67) | <0.001 |

PYAR=person years at risk; PE=protective efficacy

In all 4 arms the incidence of malaria increased with age (Figure 3), and the protective efficacies of all 3 interventions decreased over the course of the study. Supporting pharmacokinetic data suggests that the majority of children randomized to DP were not compliant with their regimen at the times they were diagnosed with malaria. Another finding of interest was that the incidence of malaria was over 5 episodes per year in children 4-5 months of age prior to randomization, suggesting the need to begin chemoprevention earlier than 6 months of age as was done in this study.

### **Figure 3. Incidence of malaria over age stratified by assigned treatment arm**


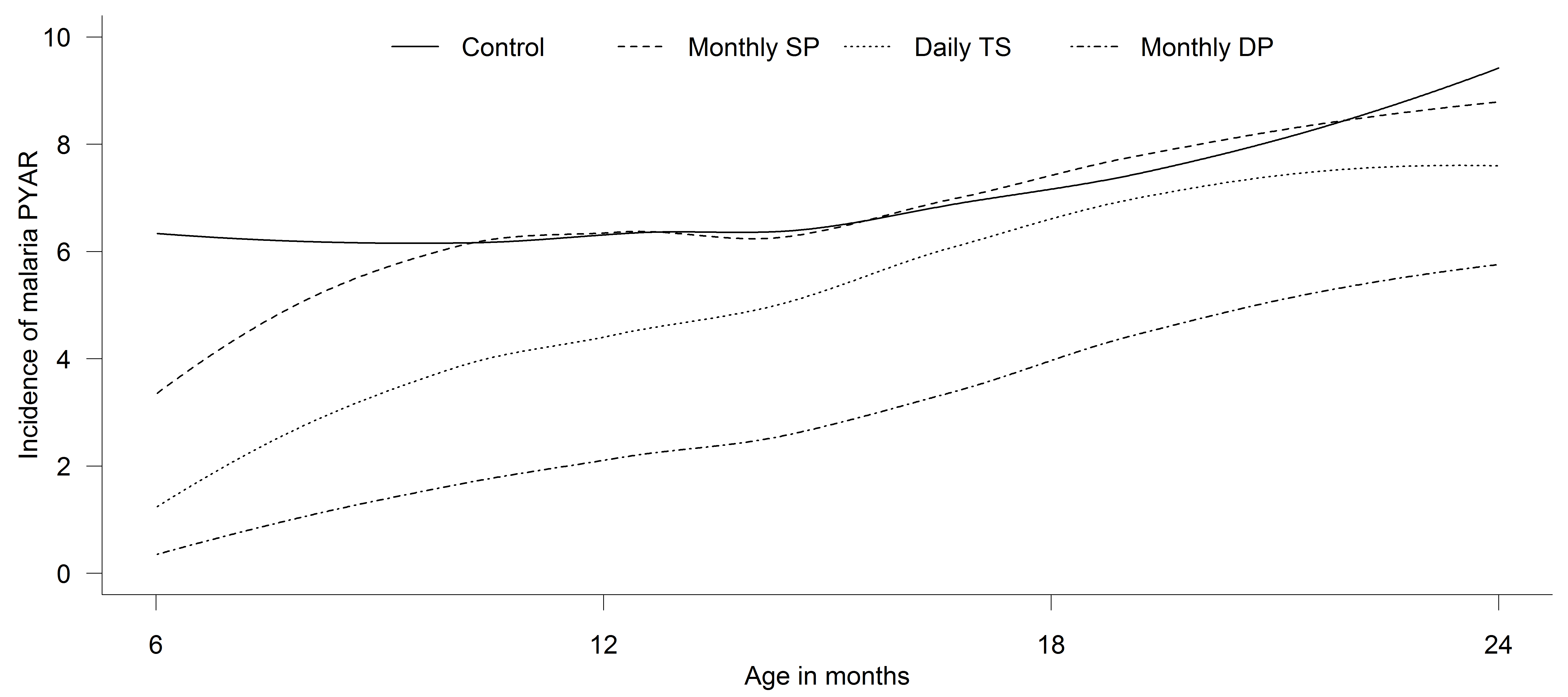


Data are also available for the one year period following the completion of the intervention (Table 2). Of 352 children who reached 24 months of age when chemoprevention was stopped; 340 reached 36 months of age and completed the study and 12 were withdrawn before reaching 36 months of age. The incidence of malaria was very high after stopping chemoprevention, reaching almost 11 episodes per year at risk. Importantly, these data do not show a “rebound” of increased malaria after completion of chemoprevention. For all arms, there has been no significant difference in the incidence of malaria following chemoprevention compared to the control group.

### **Table 2. Incidence of malaria following cessation of chemoprevention**

| **Treatment arm** | **24-36 months of age** | | | | |
| --- | --- | --- | --- | --- | --- |
|  | **Number of cases** | **PYAR** | **Incidence per PYAR** | **IRR (95% CI)** | **p value** |
| No prior therapy | 670 | 61.8 | 10.85 | 1.0 (reference) | - |
| Prior Monthly SP | 689 | 57.5 | 11.98 | 1.11 (0.88-1.40) | 0.37 |
| Prior Daily TS | 678 | 62.2 | 10.90 | 1.01 (0.80-1.27) | 0.95 |
| Prior Monthly DP | 659 | 61.2 | 10.77 | 0.99 (0.79-1.25) | 0.96 |

PYAR=person years at risk; IRR = incidence rate ratio

- 1. **Rationale**

Malaria remains one of the most important infectious diseases worldwide with an estimated 3.3 billion people at risk leading to hundreds of millions of cases and 660,000 deaths each year. In 2010, 81% of cases and 91% of deaths were estimated to have occurred in Africa, with children under five years of age and pregnant women most severely affected.[^46^](#_ENREF_46) The primary tools currently available for malaria prevention in Africa include ITNs and IPTp. However, there are limitations with these interventions and the burden of malaria remains high in many parts of Africa despite recent increases in coverage levels. ITNs do not fully protect against malaria and there is concern for waning efficacy given the emergence of resistance to pyrethroid insecticides (the only class used in ITNs). The only drug widely used for IPTp is SP, and there are recent reports suggesting that IPTp with SP is no longer effective, especially in East Africa, where resistance to this drug is now widespread. Recent increases in funding for malaria control offer an unprecedented opportunity to expand preventative interventions; however, new strategies are urgently needed to reduce the burden of malaria for those at greatest risk.

One of the most important malaria control interventions is the use of ACTs for the treatment of malaria. ACTs are highly efficacious, and now the recommended first-line treatment for uncomplicated falciparum malaria in all countries of sub-Saharan Africa , including the treatment of malaria in pregnant women during the 2^nd^ and 3rd trimesters.[^25^](#_ENREF_25) ACTs also offer the opportunity to greatly reduce the burden of malaria in pregnant women and young children if their role is expanded to chemoprevention. IPT with the ACTs artesunate-amodiaquine, artesunate-SP, and DP have been shown to be effective and safe for the prevention of malaria in African children. IPT with DP is especially attractive given its prolonged post-treatment prophylactic effect, due to the unusually long half-life of piperaquine.[^27^](#_ENREF_27)

This proposal will be the first clinical trial we are aware of to evaluate the efficacy and safety of DP for the prevention of malaria in pregnant women. We will perform a randomized, double-blinded, controlled trial to compare 2 dosing strategies of this novel intervention with the current standard of care of IPTp with SP in an area of high transmission intensity and widespread antifolate resistance. Pregnant women will undergo frequent sampling using a highly sensitive PCR assay to better define the timing and frequency of malaria infection during pregnancy and the primary outcome will be based on placental histopathology to maximize the detection of placental infection throughout pregnancy. At enrollment mothers will also consent for participation of their unborn children in the 2^nd^ phase of the study. Unborn children will be randomly assigned to receive q 3 monthly DP vs. monthly DP between 2-24 months of age and then followed for an additional 1 year after the intervention is stopped. We will be advancing the knowledge gained from our ongoing chemoprevention studies in infants by focusing on DP as the most effective candidate drug, utilizing a birth cohort and starting the intervention at an earlier age (2 months vs. 6 months) when the risk of malaria becomes high, and utilizing a double-blinded trial with directly observed therapy to more accurate assess safety and tolerability. In addition, by studying two different dosing strategies for DP in both pregnancy and infancy, we will be able compare the practical benefits of less frequent dosing with the potential greater efficacy of more frequent dosing. Perhaps the most innovative and novel aspect of this proposal will be the use of a two-phase study design that will allow us to independently test the hypotheses that prevention of malaria in pregnancy and infancy will improve the acquisition of naturally acquired antimalarial immunity and reduce the subsequent risk of malaria in infants and young children. The overall theme of this proposal will be that in areas of high malaria transmission intensity where the burden of malaria remains high despite the use of currently available control interventions, aggressive and strategic use of highly effective drugs for chemoprevention may dramatically reduce malaria associated morbidity and improve the development of antimalarial immunity.

1. **STUDY OBJECTIVES**

We will test the hypothesis that IPT with DP will significantly reduce the burden of malaria in pregnancy and infancy and improve the development of naturally acquired antimalarial immunity. The specific study objectives are as follows:

- 1. **Objective 1**

**To compare the risk of placental malaria among HIV uninfected pregnant women randomized to receive IPTp with 3 dose SP vs. 3 dose DP vs. monthly DP.** We will test the hypothesis that pregnant women who receive IPTp with either 3 doses of DP or monthly DP will have a lower risk of placental malaria defined by histopathology compared to those who receive 3 doses of SP. Secondary outcomes will include maternal and fetal clinical outcomes. We will also compare the two different dosing strategies of DP.

- 1. **Objective 2**

**To compare the incidence of malaria among infants randomized to receive q 3 monthly DP vs. monthly DP between 2-24 months of age.** We will test the hypothesis that infants randomized to receive monthly DP between 2-24 months of age will have a lower incidence of malaria during the first 24 months of life compared to infants randomized to receive q 3 monthly DP. Secondary outcomes will include the incidence of complicated malaria, hospitalizations, adverse events, and the prevalence of anemia.

- 1. **Objective 3**

**To compare the incidence of malaria among infants and children following the two phases of the intervention.** We will test the hypotheses that 1) infants born to mothers randomized to receive IPTp with 3 doses of DP or monthly DP will have a lower incidence of malaria during the first 24 months of life compared to infants born to mothers who were randomized to receive IPTp with 3 doses of SP, and 2) infants randomized to receive monthly DP between 2-24 months of age will have a lower incidence of malaria between 24-36 months of age after the intervention is stopped compared to infants randomized q 3 monthly DP between 2-24 months of age.

1. **STUDY DESIGN**

This will be a double-blinded randomized controlled phase III trial of 300 HIV uninfected pregnant women and the children born to them. The study interventions will be divided into two phases. In the first phase, HIV uninfected women at 12-20 weeks gestation will be randomized in equal proportions to one of three IPTp treatment arms: 1) 3 doses of SP, 2) 3 doses of DP, or 3) monthly DP. All three interventions arms will have either SP or DP placebo to ensure adequate blinding is achieved in the study as outlined in appendix D. Follow-up for the pregnant women will end approximately 6 weeks after giving birth. In the second phase of the study, all children born to mothers enrolled in the study will be followed from birth until they reach 36 months of age. Children born to mothers randomized to receive 3 doses of SP during pregnancy will receive DP every 3 months between 2-24 months of age. Children born to mothers randomized to receive 3 doses of DP or monthly DP during pregnancy will receive either DP every 3 months or monthly DP between 2-24 months of age. To ensure adequate blinding, children who will receive DP every 3 months will be given DP placebo during the months they will not be taking DP. Children will then be followed an additional year between 24-36 months of age following the interventions.

1. **SELECTION AND ENROLLMENT OF SUBJECTS**
   1. **Inclusion Criteria**
2. Pregnancy confirmed by positive urine pregnancy test or intrauterine pregnancy by ultrasound
3. Estimated gestational age between 12-20 weeks
4. Confirmed to be HIV uninfected by rapid test
5. 16 years of age or older
6. Residency within 30km of the study clinic
7. Provision of informed consent by the pregnant woman for herself and her unborn child
8. Agreement to come to the study clinic for any febrile episode or other illness and avoid medications given outside the study protocol
9. Plan to deliver in the hospital
   1. **Exclusion Criteria**
10. History of serious adverse event to SP or DP
11. Active medical problem requiring inpatient evaluation at the time of screening
12. Intention of moving more than 30km from the study clinic
13. Chronic medical condition requiring frequent medical attention
14. Prior SP preventive therapy or any other antimalarial therapy during this pregnancy
15. Early or active labor (documented by cervical change with uterine contractions)
    1. **Initial Screening**

We will recruit pregnant women presenting for routine care at the TDH antenatal clinic, at local health centers within 30 km, or referred by the Uganda government voluntary health teams. Pregnant women will be approached about participating in the study and will be provided an information sheet about the requirements of the study (Appendix A). If women are initially agreeable to screening for participation in the study and are < 20 weeks gestation by LMP, 16 years of age or older, and not known to be HIV infected, they will either be escorted to the study clinic or made an appointment to return at a later date. At our study clinic adjacent to the TDH antenatal clinic, study physicians will assess for initial eligibility criteria through conversation with the woman (confirming the age of the woman, residency within 30km of the study clinic, agreement to come to the study clinic for any febrile episode or other illness and avoid medications given outside the study protocol, plan to deliver in the hospital, no history of serious adverse event to SP or DP, no active medical problems requiring inpatient evaluation at the time of screening, no intention of moving more than 30km from the study clinic, no chronic medical condition requiring frequent medical attention, and no prior SP preventive therapy or any other antimalarial therapy during this pregnancy). Women who pass initial screening based on conversation with study physicians will undergo the following additional screening procedures: 1) rapid HIV testing to confirm HIV negative status, 2) ultrasound dating to confirm intrauterine pregnancy and gestational age of 12-20 weeks in conjunction with LMP (Appendix B), and 3) a pelvic examination to exclude early or active labor if clinically indicated. Women who are not eligible for the study will be referred back to the TDH antenatal clinic.

- 1. **Study Enrollment Procedures and Baseline Evaluation**

Informed consent will be obtained from women who pass initial screening. Study physicians will conduct the informed consent discussion in the study clinic. Informed consent will include both participation of the pregnant woman and her unborn child (or children in the case of non-singleton pregnancies). Informed consent will be conducted in the appropriate language and a translator will be used if necessary. The study will be described and consent obtained in one of 5 languages (Dopadhola, Ateso, Swahili, Luganda, or English). The consent forms will be translated into each language and back-translated into English to check for any loss or change of meaning. Following the informed consent discussion, pregnant women will be asked by the study physicians to sign a written consent form approved by the UCSF Committee for Human Research (UCSF CHR), Makerere University School of Medicine - Research and Ethics Committee (SOM-REC) or Makerere University School of Biomedical Sciences - Research and Ethics Committee (SBS-REC), and the Uganda National Council for Science and Technology (UNCST) for their own participation in the research study and a second approved consent form for the future use of biological specimens obtained during the course of the study. If the pregnant woman is unable to read or write, their fingerprint will substitute for a signature, and a signature from an impartial witness to the informed consent procedures will be obtained. Women will be enrolled in study on the same day that they provide informed consent for participation in the research study. On the day of enrollment, women will undergo a standardized history and physical examination, and have blood collected by venipuncture (15 cc’s) for filter paper sample (for future molecular studies), routine baseline laboratory testing and storage. Routine baseline laboratory testing will consist of a CBC and ALT measurement. Women who have history of fever in the previous 24 hours or a temperature > 38.0ºC (tympanic) will have a thick blood smear read urgently in the study clinic. Women with history of fever in the previous 24 hours or a temperature > 38.0ºC (tympanic) and a positive blood smear will be diagnosed with malaria and treated as described in section 6.2. At the end of the enrolment visit all study participants will be given a long lasting insecticide treated bed net (ITN) and a household survey appointment will be scheduled within 2 weeks to collect household-level information on the use of bednets, house members, household characteristics, and GPS coordinates (Appendix C).

1. **STUDY TREATMENT**
   1. **Treatment Group Assignments**

There will be 5 treatment arms which include both the intervention for the woman during pregnancy and her unborn child(ren) during infancy (Table 3). Non-singleton births from the same mother will be assigned to the same intervention. We will use a 2:1:1:1:1 randomization scheme targeting 100, 50, 50, 50, 50 pregnant women in treatment arms A-E respectively. A randomization list will be computer generated by a member of the project who will not be directly involved in the conduct of the study. The randomization list will include consecutive treatment numbers with corresponding random treatment assignments. Randomized codes will correspond to the 5 treatment groups using permuted variable sized blocks of 6 and 12 (to account for treatment group A being represented twice as often as the other 4 treatment groups). Sealed copies of the original randomization list and documentation of the procedure used to generate the lists will be stored in the project administrative offices in San Francisco and Kampala. Prior to the onset of the study, a set of sequentially numbered, opaque, sealed envelopes will be prepared. Each envelope will be marked on the outside with the treatment allocation number. The inside of the envelope will contain a piece of paper with the treatment allocation number and treatment group assignment along with a piece of carbon paper.

### **Table 3. Treatment arms with assignment of study drugs during pregnancy and infancy**

| **Phase of intervention** | **Treatment arm** | | | | |
| --- | --- | --- | --- | --- | --- |
|  | **A** | **B** | **C** | **D** | **E** |
| During pregnancy | 3 dose SP | 3 dose DP | 3 dose DP | Monthly DP | Monthly DP |
| During infancy | 3 monthly DP | 3 monthly DP | Monthly DP | 3 monthly DP | Monthly DP |

- 1. **Treatment Allocation**

On the day of enrollment, pregnant women will be referred to a study pharmacist responsible for treatment allocation. The study pharmacist will assign treatment arms as follows:

1. Select next available envelope
2. Note treatment number on the outside of the envelope
3. Write date, time, and study number on the outside of the envelope
4. Open envelope
5. Remove form containing code for treatment arm and date, time, and study number (transferred to form via carbon paper inside of envelope)
6. Store form in lockable file box in study pharmacy
7. Record onto the treatment allocation master list the study number, enrollment date, treatment assignment code, treatment arm, and study medications to be given during pregnancy and infancy
8. Store treatment allocation Master list in a lockable cabinet in study pharmacy
9. Record treatment number in the study participant’s file
   1. **Study Drug Dosing and Formulations**

During pregnancy, women will be given 1 of 3 treatment regimens: 1) SP given 3 times during pregnancy, 2) DP given 3 times during pregnancy, or 3) DP given every 4 weeks during pregnancy. Each treatment with SP will be given as a single dose consisting of 3 full strength tablets. Each treatment with DP will consist of 3 full strength tablets given once a day for 3 consecutive days. In addition, placebos will be used to mimic the identical dosing strategy such that every 4 weeks women will receive two pills on day 1 (SP and placebo, DP and placebo, or two placebos) followed by one pill on days 2 and 3 (DP or placebo). Two placebos will be used, one that mimics the appearance of SP and one that mimics the appearance of DP. Dosing schedules for each treatment regimen according to gestational age are presented in Appendix D. Details of the study drug formulations are included in Table 5.

During infancy, children will be given DP either every 4 weeks or every 12 weeks. Each treatment with DP will consist of half-strength tablets given once a day for 3 consecutive days according to weight based guidelines outlined in Appendix E. Infants randomized to receive DP every 12 weeks will receive placebo mimicking the dosing of DP every 4 weeks when they are not receiving study drug. Dosing schedules for each treatment regimen according to the child’s age are presented in Appendix D.

**Table 5. Drug formulation and labeling**

| **Drug** | **Formulations** | **Trade name (Manufacturer)** |
| --- | --- | --- |
| Sulfadoxine-Pyrimethamine (SP) | 500mg/25mg tabs | Kamsidar (KPI) |
| Dihydroartemisinin-Piperaquine (DP)  Full strength tablets | 40mg/320mg tabs | Duo-Cotexin (Holley-Cotec) |
| Dihydroartemisinin-Piperaquine (DP)  Half strength tablets | 20mg/160mg tabs | Duo-Cotexin (Holley-Cotec) |

- 1. **Blinding, Study Drug Administration, and Duration**

Administration of all study drugs will be double blinded. All doses of study drugs will be prepackaged by a study pharmacist and administered by a study nurse blinded to the study participant’s treatment regimen. All doses of SP (or SP placebo) administered will be directly observed in the clinic. For DP (or DP placebo), the first of the 3 daily doses will be directly observed in the clinic and the 2^nd^ and 3^rd^ daily doses will be administered at home using pre-packaged study drugs in opaque envelopes with dosing instructions written on the outside. For doses of study drugs administered in the clinic, if a study participant vomits the study drug within 30 minutes of administration, the drug will be re-administered. For doses of study drugs administered at home, if a study participant vomits the study drug within 30 minutes of administration or study drug is lost, the study participant will be instructed to come to the study clinic as soon as possible where the study drug will be re-administered/replaced. For pregnant women all doses of study drugs will be given between 16 and 40 weeks gestation and for children all doses of study drugs will be given between 8 and 104 weeks (2 years) of age as outlined in Appendix D.

- 1. **Study Drug Accountability**

The study pharmacist will maintain complete records of all study drugs received in the study pharmacy. Lot number and number of pills given to each study participant will be recorded. A registry of all study medication, current product labels, and Certificates of Analysis, provided by suppliers will be maintained within the regulatory binder for the study. The date received, lot number, expiration date, and date used will be recorded for each of the study medications. Monthly inventory of all study medications will be conducted and a record log of investigational medications will be kept at the study clinic.

1. **SUBJECT MANAGEMENT**
   1. **Subject Follow-up**

Pregnant women will be scheduled to be seen in the clinic every 4 weeks during their pregnancy and 6 weeks following delivery. In addition, pregnant women will be instructed to come to the study clinic for all their medical care and avoid the use of any outside medications. Children will be scheduled to be seen in the clinic every 4 weeks and parents /guardians of children will be instructed to bring their child to the study clinic for all medical care and avoid the use of any outside medications. The study clinic will remain open 7 days a week from 8 a.m. to 5 p.m. Pregnant women and children who are not seen on the day of their regularly scheduled visits will be visited at home and instructed to come to the clinic as soon as possible.

Each time a study participant is seen in the clinic a standardized history and physical exam will be performed including temperature, pulse, and blood pressure measurement. Patients who are febrile (tympanic temperature > 3 8.0˚C) or report history of fever in the past 24 hours will have blood obtained by finger prick for a thick blood smear (in very young children, heel sticks may be substituted for finger pricks). If the thick blood smear is positive, the patient will be diagnosed with malaria. If the thick blood smear is negative, the patient will be managed by study physicians for a non-malarial febrile illness (Section 6.3). If the patient is afebrile and does not report a recent fever, a thick blood smear will not be obtained, except when following routine testing schedules (Section 6.5).

- 1. **Diagnosis and Management of Malaria**

Patients found to have malaria based on laboratory confirmation will have a second finger prick for a thin smear (to determine parasite species), hemoglobin measurement using a portable spectrophotometer (HemoCue), and collection of plasma for future PK studies. All episodes of malaria will be classified as uncomplicated or complicated based on the following criteria:

**Uncomplicated malaria (all of the following)**

1) Fever (> 38.0ºC tympanic) or history of fever in the previous 24 hours

2) Positive thick blood smear

3) Absence of complicated malaria

**Complicated malaria (any of the following)**

1) Evidence of severe malaria (Appendix F) and parasitemia

2) Danger signs present in children (Appendix F) and parasitemia

Episodes of malaria will also be classified into the following categories according to the timing of previous malaria episodes for treatment purposes:

1. New episodes of malaria will be defined as any first episode or any episode occurring > 14 days after the diagnosis of a previous episode
2. Treatment failures will be defined as any of the following:
   1. Complicated malaria occurring 1-14 days after the diagnosis of a previous episode
   2. Fever (> 38.0°C tympanic) or history of fever in the previous 24 hours with a parasite density > the parasite density of an episode of malaria diagnosed 2 days prior
   3. Fever (> 38.0°C tympanic) or history of fever in the previous 24 hours with a parasite density > 25% of the parasite density of an episode of malaria diagnosed 3 days prior
   4. Fever (> 38.0°C tympanic) or history of fever in the previous 24 hours with a positive thick blood smear of any parasite density occurring 4-14 days after the diagnosis of a previous episode

All patients diagnosed with new episodes of uncomplicated malaria will be prescribed AL, the recommended first-line treatment in Uganda for children and pregnant women in their 2^nd^ or 3^rd^ trimester. Patients with complicated malaria will be prescribed quinine or artesunate according to national malaria treatment guidelines. Children who are less than 4 months of age or weigh <5kg, will be treated with quinine for uncomplicated malaria in accordance with the Uganda Ministry of Health Guidelines. Patients with treatment failure within 14 days following treatment with AL will be prescribed quinine according to national malaria treatment guidelines. Patients with treatment failure within 14 days following treatment with quinine or artesunate will be treated with quinine plus clindamycin or artesunate.

- 1. **Management of Non-Malaria Illnesses**

Patients who are found to have illnesses other than malaria will receive standard-of-care treatment in the study clinic, according to standardized algorithms, or will be referred to the Tororo District Hospital. We will avoid the routine use of non-study medications with antimalarial activity, including tetracyclines, antifolates (with the exception of assigned chemopreventive regimens), and macrolide antibiotics, when acceptable alternatives are available. In addition, drugs with known risk of torsades de pointes or CYP3A inhibitors (Appendix K and L) will be avoided when prescribing treatment to pregnant women or children during the time they are taking study drugs. If the study clinician deems treatment with one of the drugs listed on Appendix K or L is required, the clinical management team will be consulted. During follow-up for non-malarial illnesses, blood smears will be done at the discretion of the study physician if the subjects are febrile (tympanic temperature > 38.0˚C) or report history of fever in the past 24 hours. If the blood smear is positive, the patient will be diagnosed with a new episode of malaria and managed per study protocol.

- 1. **After Hours Visits**

Pregnant women will be encouraged to come to the TDH maternity ward (open 24 hours a day) and parents/guardians will be encouraged to bring their child to the TDH pediatric inpatient ward (open 24 hours a day) when urgent care is needed outside of study clinic hours. Pregnant women or parents/guardians of children will be instructed to inform hospital personnel of their involvement in the study at the time of registration and to visit the study clinic on the following day. If a patient is diagnosed with uncomplicated malaria they will receive treatment from a hospital supply of AL and the doctors will be instructed to refer patients to our study clinic when it opens at 8 am the following day. If a patient is diagnosed with severe malaria, he/she will receive quinine or artesunate following standard treatment guidelines. Patients with non-malarial illnesses will be managed at the discretion of the Tororo District Hospital staff. Upon discharge, patients will receive follow-up at the study clinic as outlined above. Study personnel will visit the Tororo District Hospital daily to inquire about visits from study subjects and facilitate follow-up in the study clinic.

- 1. **Routine Assessments**

Routine assessments will be done in the clinic every 4 weeks for both pregnant women and children. Study participants not seen in the clinic for their every 4 week routine visits will be visited at home and requested to come to the study clinic as soon as possible. Pregnant women and children will receive standards of care as designated in the Uganda MOH guidelines (Appendix G). Routine antenatal care will include screening and treatment for sexually transmitted Infections, blood pressure assessment, urine dipstick for proteinuria, prescription of iron, folate, multivitamins and mebendazole. Routine care in children will include immunizations, vitamin A supplementation, and management of anemia using Integrated Management of Childhood Illness (IMCI) guidelines. During routine assessments subjects will be asked about visits to outside health facilities and the use of any medications outside the study protocol. The study protocol will be reinforced with discussion regarding the need to come to the study clinic promptly upon the onset of any illness and to avoid use of outside medications. Standardized assessment of adherence will also be done for study drugs administered at home and ITN use. A routine history and physical exam will be performed using a standardized clinical assessment form. Blood will be collected by finger prick (in very young children, heel sticks may be substituted for finger pricks) for thick smear, collection of plasma for PK studies, and filter paper samples. If a pregnant woman or parent/guardian of a child reports a fever in the last 24 hours or the patient has a documented temperature > 38.0˚C tympanic, the patient’s thick blood smear will be read immediately and if positive the patient will be diagnosed and treated for malaria (see section 6.2). Phlebotomy for routine laboratory tests (CBC and ALT) to monitor for potential adverse events from study medications and for immunology studies will be performed every 8 weeks in pregnant women and every 16 weeks in children. Screening for non-malaria parasitic infections will be performed once in pregnant women after the 1^st^ trimester at the time routine medendazole is administered and in children at 1yearof age. Screening will consist of stool ova and parasite examination, circulating filarial antigens (by ICT card for *Wucheria*), and blood smear for microfilaremia (including *Mansonella perstans*) using Knott’s technique. For pregnant women and children 2-24 months of age, study drugs will be administered at the time of each routine visit as described in sections 5.3 and 5.4.

- 1. **Delivery visit**

Addressing one of the main study outcomes, placenta malaria, requires collection and processing of specimens at delivery. Systems will be in place to facilitate a birth plan which will encourage women to come to the hospital for delivery, including access to transportation 24 hours a day. However for women who are unable to travel to the hospital for delivery or choose to deliver at home, a study staff member will be driven to the home to follow study procedures. Study staff will document details of the delivery, including date and time, type of delivery, estimated blood loss and any maternal, obstetrical or neonatal complications. Study staff will document the infant’s Apgar score and birth weight with calibrated scales. Biological samples collected at the time of delivery will include maternal venous blood (for thick blood smear, filter paper samples, CBC, ALT, immunology studies, and PK studies), cord blood (for thick blood smear, filter paper samples, hemoglobin measurement, and immunology studies) and placental tissue. Following delivery, neonatal care, as per national guidelines, will include polio and BCG immunization, ophthalmic tetracycline, vitamin K, and vitamin A supplementation.

At the time of delivery, women will undergo repeat rapid HIV testing based on national guidelines. If women are found to have become HIV-infected during pregnancy, both the mother and their newborn will be withdrawn from the study and immediately referred for care following local prevention of mother-to-child transmission (PMTCT) guidelines.

- 1. **Postpartum visits.**

Women will be evaluated within 1 week following delivery and 6 weeks postpartum as part of routine care. Both visits will include an abdominal exam syndromic management of STIs, and follow-up on any obstetrical complications that occurred including evaluation of the neonate for any congenital abnormalities. Pelvic and breast exam will be done if clinically indicated. Contraceptive counseling, nutritional assessment and infant feeding and support will be provided. Following the 6 weeks visit, women will no longer be considered study participants, although most will remain primary care givers for their children enrolled in the second phase of the study.

Summaries of all procedures done during routine visits in pregnant women and children are presented in Appendix H and I, respectively.

- 1. **Medical Care Outside the Study Clinic**

We will provide routine medical care in our clinic free of charge, including medications, to the extent possible given resources available. Study participants and their guardians we will be reimbursed for costs of any transportation to and from our clinic. In addition, we will reimburse the cost of tests and drugs for referrals of study participants made by study physicians to other clinics and services as well as after-hours visits at TDH. We anticipate reimbursing the cost of most diagnostic tests (including laboratory tests, X-rays, and ultrasounds) and medications resulting from these referrals, using available funds. However, reimbursement of all diagnostic tests and treatment recommended outside the study clinic cannot be guaranteed in all circumstances. Decisions on reimbursement will be made by the study coordinator and the investigators, in conjunction with the funding agency if necessary.

- 1. **Duration of Follow-up and Criteria for Premature Study Withdrawal**

Pregnant women will be followed until their 6 week postpartum visit. Children will be followed through 30^th^ June 2018 until they reach 36 months of age. Women will be asked to bring in the biological father to the study clinic for informed consent discussion when child is born. He will be asked to provide written consent or a fingerprint to allow participation of the infant. If a father is unknown or unavailable, the woman will document in accordance with UNCST guidance and participation will continue for both the woman and infant. If the biological father refuses consent for the child’s participation, the child will not be enrolled.

Study participants will be prematurely withdrawn from the study for: 1) movement out of study area or inability to be located for > 60 consecutive days, 2) withdrawal of informed consent, 3) inability to comply with the study schedule and procedures, 4) at the discretion of the site investigator if the study is not in the best interest of the participant, 5) subject or parent/guardian judged by the site investigator to be at significant risk of failing to comply with the study protocol as to cause them harm or seriously interfere with the validity of study results, 6) women found to have become HIV infected at the time of delivery, or 7) father does not consent to the child’s participation (child withdrawal only). If a subject is withdrawn for reasons # 1 or 2, we will be unable to perform any additional study procedures. If a subject is withdrawn for reasons # 3-5, plans to obtain appropriate follow-up tests outside of the study will be individualized for each subject depending on the health status of the subject at the time of withdrawal and the willingness of the participant and his or her parent/guardian to proceed with additional testing. If a subject is withdrawn for #6-7, the will be referred for appropriate care following local PMTCT guidelines.

- 1. **Diagnostic and Laboratory Testing**
     1. **Microscopy**

Thick and thin blood smears will be stained with 2% Giemsa and read by experienced laboratory technologists. Parasite densities will be calculated by counting the number of asexual parasites per 200 leukocytes (or per 500 leukocytes, if the count is <10 asexual parasites/200 leukocytes), assuming a leukocyte count of 8,000/μl. A blood smear will be considered negative when the examination of 100 high power fields does not reveal asexual parasites. Gametocytemia will also be determined from thick smears. Thin smears will be used for parasite species identification. For quality control, all slides will be read by a second microscopist and a third reviewer will settle any discrepant readings.

- - 1. **Clinical Laboratory Studies**

At enrollment and every 8 weeks during follow-up for pregnant women and every 16 weeks for children, venipuncture blood samples will be collected for routine clinical laboratory studies, including CBC and ALT measurements. Additional venipunctures will be performed, as appropriate, for laboratory testing to evaluate non-malarial medical illnesses at the discretion of study physicians. Results will be made available to study physicians in a timely manner for patient management decision-making. Additional hemoglobin measurements will be performed each time a patient is diagnosed with malaria using a portable spectrophotometer (HemoCue, Angholm, Sweden) and results will be immediately available.

- - 1. **Placental Studies**

Cord blood will be collected for immunology studies described below. Placental blood collected for Giemsa-stained blood smears and PCR will be obtained by making a small incision on the maternal surface of the placenta within 1 hour of delivery, collecting blood from the intervillous space via syringe, and then placing the blood in an EDTA tube. Placental tissue will be collected for histological assessment. Two 1 cm-wide full thickness biopsies from each placenta, obtained about 5 cm from the cord, will be obtained within 1 hour of delivery and placed in 10% neutral buffered formalin. Biopsy specimens will be embedded in paraffin wax, sectioned into 3 μM slices using a rotary microtome, fixed to glass slides, and dehydrated in sequential ethanol baths. Separate slides will then be stained in 0.1% hematoxylin and 1% eosin for 5 and 1 min, respectively, or in 2% Giemsa for 30 minutes. All necessary expertise and infrastructure for these studies is in place in Tororo. For assessment of histologic evidence of placental malaria, placentas will be graded into 5 categories using a standardized approach.[^80^](#_ENREF_80) The presence of intervillous parasite-infected erythrocytes and of pigment in monocyte/macrophages or fibrin will be noted. Quantitative assessments of placental malaria will be as follows. First, 1000 intervillous blood cells will be counted under high power. Percentages of intervillous erythrocytes infected with parasites and of monocyte/macrophages containing malarial pigment will be counted. Placental specimens will be examined by two experts, and any discrepant readings will be resolved by a third reader.

- - 1. **Molecular and Parasitology Studies**

Each time a thick blood smear is obtained; blood will also be collected onto filter paper. Samples will be collected by venipuncture or by finger prick sampling. Blood will be placed onto filter paper in approximately 25 μl aliquots per blood spot (4 blood spots per sample). The samples will be labeled with study numbers and dates, air-dried, and stored in small, sealed sample bags at ambient temperature or 4°C with desiccant. Molecular studies will include the extraction of DNA from filter paper and followed by characterization of parasite genetic polymorphisms using standard molecular procedures including PCR, DNA hybridization, and/or restriction enzyme digestion. Additional molecular studies will include analyses of polymorphisms in parasite genes for mutations that may impact on malaria infection and response to antimalarial therapy. Molecular studies will be performed only for research purposes and will have no impact on the clinical management of study patients. Molecular studies will be formed in Uganda whenever possible and at UCSF when facilities/equipment are not available in Uganda.

Blood collected by venipuncture on the day malaria in child is diagnosed will be used in selected subjects for parasite culture and/or immunology studies (below). For these studies the skin will be prepped with 3 washes with a betadine or equivalent sterilizing solution, and then approximately 3-5 mls of blood in children <2 years of age, or 6-10 mls of blood in children ≥2 years of age will be collected in an anticoagulated sterile tubes and transferred promptly (generally within 30 minutes) to our molecular laboratory. Parasites will be cultured following standard protocols. In brief, erythrocytes will be separated from plasma by centrifugation and removal of the supernatant and buffy coat, and the infected erythrocytes will then be cultured in RPMI medium supplemented with human serum or Albumax serum substitute. Cultured parasites will be evaluated for in vitro drug sensitivity, molecular characteristics, and other features to characterize antimalarial drug resistance and other aspects of malaria. Information from the parasitology studies will have no impact on patient care.

- - 1. **Immunology Studies**

Venipuncture blood samples collected at enrollment, during routine visits done every 8 weeks in pregnant women and every 16 weeks in children, and in select subjects on the day malaria is diagnosed (above) will be made available for immunology studies. Approximately 15mls of blood in pregnant women and 5mls of blood in children will be collected and separated into plasma and peripheral blood mononuclear cells (PBMC) using a Ficoll gradient, following standard protocols.  Plasma will be stored at -80°C for future immunologic, which may include measurement of levels of cytokines, antibodies, and other features related to the host immune response.  PBMCs will be stored in liquid nitrogen to maintain viability, and will be evaluated using flow cytometry, ELISPOT, RT-PCT, expression microarrays and other assays to assess the host immune response.  Stool will be collected at 1, 2, and 3 years of age in children and evaluated for parasitic infections, and remaining stool will be stored for future studies analyzing the influence of parasitic and bacterial infections on immunity. Information from immunology studies will have no impact on patient care. Immunology studies will be done in Uganda whenever possible. However, due to technological limitations some immunology studies may be done at UCSF.

- - 1. **Pharmacokinetic (PK) Studies**

As resources permit, stored plasma samples and dried blood spots on filter paper will be available for measuring DHA and piperaquine levels in a subset of patients who are randomized to receive DP. PK studies will be done at UCSF as the capacity for performing these assays does not exist in Uganda.

- 1. **Co-enrollment Guidelines**

The pregnant women and children from this study may be co-enrolled in observational studies. They may not be co-enrolled in protocols that utilize concomitant study medications. Co-enrollment in other studies will be determined on a case-by-case basis by the protocol team.

- 1. **Management of Adverse Events Potentially Related to Study Drugs**

The following section outlines management of adverse events potentially related to study drugs (SP or DP). Given the double blinded nature of the intervention, study clinicians and study participants will not be aware of what study drugs are being administered. Therefore all study participants will be considered potentially exposed to either study drug during the period study drugs are first given up to one month after the last dose of study drug is given.

- 1. **Grade 1 or 2 Adverse Events**

Participants who develop grade 1 or 2 adverse events may continue study drugs. The study clinicians will manage the grade 1 or 2 events according to standard practice.

- 1. **Grade 3 or 4 Adverse Events**

Management will be as follows:

- Repeat observation or lab test within 72 hours of observation or of receiving lab results report.
- For grade 3 or non-life threatening grade 4 adverse events, subjects may continue taking study drugs pending clinic visit or repeat laboratory tests. Clinician has the option of immediately stopping the study drugs if subject cannot be examined in clinic, if a repeat laboratory test cannot be performed within 72 hours, or if the clinician determines that the continuation of study drugs is unsafe while awaiting clinic exam or test results.
- For grade 4 life-threatening adverse events, study clinicians should hold study drugs pending laboratory confirmation.
- Work-up to exclude other causes.
- For all grade 3 or 4 adverse events supported by repeat clinical exam or laboratory test results, study drugs will be held, and laboratory tests will be repeated every 1-2 weeks, until the adverse event resolves to < grade 2 unless there is strong evidence that the adverse event is not related to either study drug.
- If the adverse event persists at grade 3 or 4 for more than 28 days or recurs on re-challenge, and the adverse event is thought to be possibly related to one of the study drugs, the study drugs will be permanently discontinued.

In the event that study drugs are permanently discontinued, study participants will remain in the study, following our intention-to-treat analysis approach. In the event that study drugs are permanently discontinued, study clinicians may become un-blinded to the study participant assigned treatment regimen if this knowledge may assist in the management of the grade 3 or 4 adverse event(s) that lead to the permanent discontinuation of the study drugs.

1. **MONITORING OF ADVERSE EVENTS AND MANAGEMENT**
   1. **Monitoring and Reporting of Adverse Events**
      1. **Definitions**

An adverse event is defined as "any untoward medical occurrence in a patient or clinical investigation subject administered a pharmaceutical product that does not necessarily have a causal relationship with this treatment" (ICH Guidelines E2A). An adverse event can further be broadly defined as any untoward deviation from baseline health, which includes:

- Worsening of conditions present at the onset of the study
- Deterioration due to the primary disease
- Intercurrent illness
- Events related or possibly related to concomitant medications

(International Centers for Tropical Disease Research Network Investigator Manual, Monitoring and Reporting Adverse Events, 2003).

- - 1. **Identification of Adverse Events**

At each scheduled and unscheduled visit to the clinic, study clinicians will assess patients according to a standardized case record form. A severity grading scale, based on toxicity grading scales developed by the NIH Divisions of AIDS (DAIDS) and the Division of Microbiology and Infectious Diseases (DMID) Pediatric Toxicity Tables, will be used to grade severity of all symptoms, physical exam findings, and laboratory results (Appendix J). All participants, regardless of treatment arm, will be assessed using the same standardized case record form. Adverse event monitoring will occur during the period when study drugs are given and up to 1 month after cessation of study drugs.

Data will be captured on the incidence of all adverse events, regardless of severity. For each adverse event identified and graded as severe or life threatening and felt to be possibly, probably or definitely related to study drugs, an adverse event report form will be completed. In addition, an adverse event form will be completed for all serious adverse events and unexpected events, regardless of severity. An adverse event report form will not be completed for events classified as mild or moderate (unless they are serious or unexpected), as mild and moderate symptoms are common and difficult to distinguish from signs and symptoms due to malaria and other common illnesses. The following information will be recorded for all adverse experiences that are reported:

1. Description of event
2. Date of event onset
3. Date event reported
4. Maximum severity of the event
5. Maximum suspected relationship of the event to study drugs (either SP or DP)
6. Whether the event is a serious adverse event
7. Initials of the person reporting the event
8. Outcome
9. Date event resolved
   - 1. **Reporting of Adverse Events**

Guidelines for reporting of adverse events provided by NICHD, UCSF Committee for Human Research (CHR), and the Food and Drug Administration (FDA) in the U.S. and the Makerere University IRB (SOMREC), and National Drug Authority (NDA) in Uganda will be followed as summarized in Table 6 below.

**Table 6**. Guidelines for reporting adverse events

| **Institution** | **Type of Adverse Events** | **When to Report** |
| --- | --- | --- |
| **NICHD** | - Definitely, Probably, or Possibly related **AND** Serious or Unexpected | - Within 10 working days of awareness |
| **UCSF-CHR** | External [off-site] adverse event that UCSF PI determines:   - changes the study risks or benefits,   OR   - necessitates modification to the CHR-approved consent document(s) and/or the CHR-approved application/protocol | - Within 10 working days of PI’s awareness |
| **MU-SBSREC** | - All Serious and Unexpected events irrespective of relationship; | - Fatal or life-threatening events within 3 working days of awareness - All other SAEs within 7 calendar days |
| **NDA** | - All Serious and Unexpected events irrespective of relationship | - Within 7 calendar days of awareness |
| **FDA** | - Definitely, Probably or Possibly related **AND BOTH** Serious^*^ AND Unexpected^±^ | - For fatal or life-threatening events, by telephone or fax within 7 calendar days of first awareness - All other reportable events within 15 calendar days of first awareness |

***Serious Adverse Event (SAE)*** is any AE that results in any of the following outcomes:

• Death,

• Life-threatening adverse experience.

• Inpatient hospitalization or prolongation of existing hospitalization,

• Persistent or significant disability/incapacity,

• Congenital anomaly/birth defect, or cancer, or

• Any other experience that suggests a significant hazard, contraindication, side effect or precaution that **may require medical or surgical intervention** to prevent one of the outcomes listed above,

• Event occurring in a gene therapy study

• Event that changes the risk/benefit ratio of the study.

***Unexpected Adverse Event*** An adverse event is defined as being unexpected if the event exceeds the nature, severity, or frequency described in the protocol, consent form and investigator brochure (when applicable). An unexpected AE also includes any AE that meets any of the following criteria:

• Results in subject withdrawal from study participation,

• Due to an overdose of study medication, or

• Due to a deviation from the study protocol

1. **STATISTICAL CONSIDERATIONS**
   1. **Hypothesis 1**

We will test the hypothesis that pregnant women who receive IPTp with either 3 dose DP or monthly DP will have a lower risk of placental malaria defined by histopathology compared to those who receive 3 doses of SP. Secondary outcomes will include maternal and fetal clinical outcomes. We will also compare the two different dosing strategies of DP.

- - 1. **Primary Outcome**

The primary outcome will be the prevalence of placental malaria based on placental histopathology and dichotomized into any evidence of placental infection (parasites or pigment) vs. no evidence of placental infection. We will also evaluate placental malaria defined by histopathology as a categorical variable (active-acute, active-chronic, and past infection) based on the criteria developed by Rogerson et al.[^80^](#_ENREF_80)

- - 1. **Secondary Outcomes**

Secondary outcomes are summarized in Table 7 below.

### **Table 7. Secondary outcomes**

| **Secondary outcome** | **Definition** |
| --- | --- |
| Placental parasitemia | Proportion of placental blood samples positive for parasites by microscopy or PCR |
| Maternal malaria | Any treatment for malaria |
| Composite birth outcome | Congenital malformations, late spontaneous abortion (<28 weeks), LBW (<2500g), still birth (fetal demise ≥28 weeks), congenital anomaly, or preterm delivery (<37 weeks). |
| Incidence of adverse events | Adverse events stratified by type, severity score and relationship to study drugs |
| Prevalence of anemia | Proportion of routine hemoglobin measurements < 10 g/dL & < 8 g/dL |
| Prevalence of asymptomatic parasitemia | Proportion of routine monthly samples positive for parasites by PCR |

- - 1. **Analyses**

An intention-to-treat approach to all analyses will be used, including all study participants randomized to therapy and including all follow-up time until the study participants complete the study or early study termination regardless of whether the intervention was stopped due to an adverse event.

**Primary analysis.** We will compare the prevalence of placental malaria defined by histopathology at birth between the study arms using the Chi-Square test for all pair-wise comparisons. We will explore for any differences of potential confounders between the treatment arms and if necessary adjust our analysis using multivariate logistic regression.

**Secondary analyses.** We will compare the prevalence of placental parasitemia and our composite birth outcome using the Chi-Square test. We will compare the incidence of maternal malaria and adverse events using Poisson or negative binomial regression models. The Poisson models will include the logarithm of the follow-up time as an offset. We will translate the fitted coefficients and their confidence bounds into percentage effects with the formula 100*[exp(coefficient)-1]. This approach is closely related to exponential survival models for analyzing events per follow-up time, but is better able to adjust for violated assumptions. Testing for overdispersion in the Poisson regression can detect violations of these assumptions, and variances can be adjusted accordingly to produce valid p-values and confidence interval. If significant deviations from required distributions in study data are detected, we will employ negative-binomial or zero-inflated negative-binomial models to account for the observed pattern of data. We will compare the prevalence of maternal anemia and asymptomatic parasitemia using generalized estimating equations with adjustments for repeated measures in the same study participant. If necessary, multivariate analyses will be performed to adjust for potential confounders and effect modifiers.

- 1. **Hypothesis 2**

We will test the hypothesis that infants randomized to receive monthly DP between 2-24 months of age will have a lower incidence of malaria during the first 24 months of life compared to infants randomized to receive q 3 monthly DP. Secondary outcomes will include the incidence of complicated malaria, hospitalizations, adverse events, and the prevalence of anemia, asymptomatic parasitemia, and gametocytemia. This hypothesis will be limited to only infants born to mothers assigned to receive IPTp with 3 dose DP or monthly DP to ensure an equal balance of this prior phase of the interventions.

- - 1. **Primary Outcome**

The primary outcome will be the incidence of malaria, defined as the number of incident episodes per time at risk. Incident cases will include all treatments for malaria not proceeded by another treatment in the previous 14 days. Time at risk will begin at birth and will end when study participants reaches 24 months of age, when the intervention will be stopped, or early study termination (if prior to 24 months of age).

- - 1. **Secondary Outcomes**

Secondary outcomes are summarized in Table 8 below.

### **Table 8. Secondary outcomes**

| **Secondary outcome** | **Definition** |
| --- | --- |
| Incidence of complicated malaria | Any treatment for malaria meeting criteria for severe malaria or danger sings |
| Incidence of hospital admissions | Admission to the pediatric ward for any cause |
| Incidence of adverse event | Adverse events stratified by type, severity score and relationship to study drugs |
| Prevalence of anemia | Proportion of routine hemoglobin measurements < 10 g/dL & < 8 g/dL |
| Prevalence of asymptomatic parasitemia | Proportion of routine samples (PCR or blood smears) positive for asexual parasites. |
| Prevalence of gametocytemia | Proportion of routine blood smears positive for gametocytes. |

- - 1. **Analyses**

An intention-to-treat approach to all analyses will be used, including all infants born to mothers assigned to receive IPTp with 3 dose DP or monthly DP and including all follow-up time until the study participants reach 24 months of age or early study termination regardless of whether the intervention was stopped due to an adverse event.

**Primary analysis.** We will compare the incidence of malaria using Poisson or negative binomial regression models as described in section 9.1.3. In necessary, multivariate analyses will be performed to adjust for potential confounders and effect modifiers.

**Secondary analyses.** We will compare the incidence of complicated malaria, hospitalizations, and adverse events using Poisson or negative binomial regression models as described in section 9.1.3. We will compare the prevalence of anemia, asymptomatic parasitemia, and gametocytemia using generalized estimating equations with adjustments for repeated measures in the same study participant. In necessary, multivariate analyses will be performed to adjust for potential confounders and effect modifiers.

- 1. **Hypothesis 3**

We will test the hypotheses that A) infants born to mothers randomized to receive IPTp with 3 dose DP or monthly DP will have a lower incidence of malaria during the first 24 months of life compared to infants born to mothers who were randomized to receive IPTp with 3 doses of SP, and, B) infants randomized to receive monthly DP between 2-24 months of age will have a lower incidence of malaria between 24-36 months of age after the intervention is stopped compared to infants randomized q 3 monthly DP between 2-24 months of age. For hypothesis 3A, we will only include infants randomized to receive q 3 monthly DP during infancy. For hypothesis 3B, we will also explore for interaction with the intervention given during pregnancy.

- - 1. **Primary Outcome**

The primary outcome will be the incidence of malaria, defined as the number of incident episodes per time at risk. Incident cases will include all treatments for malaria not proceeded by another treatment in the previous 14 days. For hypothesis 3A, time at risk will begin at birth and will end when study participants reaches 24 months of age or early study termination (if prior to 24 months of age). For hypothesis 3B, time at risk will begin at 24 months of age and will end when study participants reaches 36 months of age or early study termination (if between 24-36 months of age).

- - 1. **Secondary Outcomes**

The incidence of complicated malaria will be included as a secondary outcome for both hypothesis 3A and 3B.

- - 1. **Analyses**

An intention-to-treat approach to all analyses will be used. For hypothesis 3A, we will only include infants randomized to receive q 3 monthly DP during infancy. For hypothesis 3B, we will include all infants that reach 24 months of age. We will compare the incidence of malaria and the incidence of complicated malaria using Poisson or negative binomial regression models as described in section 9.1.3. For hypothesis 3B, we will also explore for interaction with the intervention given during pregnancy.

- 1. **Sample size and power**

The number of pregnant women enrolled and the number of study participants reaching the various endpoints will determine the samples sizes for each of the primary outcomes of our study aims (Table 9). The primary determinant of our target sample size was based on testing hypotheses 3 given that the magnitude of differences anticipated for this hypothesis would be smaller than those anticipated for hypothesis 1 and 2. We conservatively estimate that 90% of pregnant women enrolled will reach the primary study endpoint and we will lose 5% of follow-up time per year in the infants. The minimum relative differences detectable for the primary outcomes of the 3 hypotheses given our estimated sample sizes are summarized in Table 10 below. For hypotheses 1, we will be powered to detect a 33% relative difference in the prevalence of placental malaria in the 3 dose DP arm or the monthly DP arm compared to the 3 dose SP arm assuming a prevalence of placental malaria of 62% in the 3 dose SP arm based on prior data. We will also be powered to detect a 41-70% relative difference in the prevalence of placental malaria in the monthly DP arm compared to the 3 dose DP arm assuming a prevalence of placental malaria in the 3 dose DP arm ranging from 20-50%. For hypothesis 2, we will be powered to detect an 18-23% relative difference in the incidence of malaria between 0-24 months of age in the monthly DP arm compared to the q 3 monthly DP arm assuming an incidence of malaria in the q 3 monthly DP arm ranging from 3-5 episodes PPY. For hypothesis 3A, we will be powered to detect a 22-28% relative difference in the incidence of malaria between 0-24 months of age among children assigned to receive q 3 monthly DP when comparing those born to mothers who received 3 dose SP to those born to mothers who received 3 dose DP or monthly DP assuming an incidence of malaria in the control group ranging from 3-5 episodes PPY. For hypothesis 3B, we will also be powered to detect a 16-21% relative difference in the incidence of malaria between 24-36 months of age among children assigned to receive monthly DP during infancy compared to those who received q 3 monthly DP during infancy assuming an incidence of malaria in the control group ranging from 3-5 episodes PPY.

Table 9. Intervention arms during pregnancy and infancy

| **Study populations** | **Intervention arms** | | | | |
| --- | --- | --- | --- | --- | --- |
| Assigned treatment arms in pregnant women | 3 doses of SP | 3 doses of DP | | monthly DP | |
| Estimated number of pregnant women enrolled | 100 | 100 | | 100 | |
| Estimated number with placental outcomes | 90 | 90 | | 90 | |
| Assigned treatment arms in infants | q 3 monthly DP | q 3 monthly DP | monthly DP | q 3 monthly DP | monthly DP |
| Estimated number of infants enrolled | 90 | 45 | 45 | 45 | 45 |
| Estimated number of infants reaching 24 months of age | 81 | 41 | 41 | 41 | 41 |

Table 10. Minimum relative differences in outcomes detectable given estimated effective sample sizes

| **Hypothesis** | **Analysis population** | **Control group and**  **estimated sample size** | **Comparison group and estimated sample size** | **Estimated outcome measure in control group** | **Minimum relative difference detectable*** |
| --- | --- | --- | --- | --- | --- |
| 1 | Pregnant women with placental malaria measured at birth | 3 dose SP  (n=90) | 3 dose DP or monthly DP (n=90 each) | Prevalence of placental malaria = 62% | 33% |
|  |  | 3 dose DP  (n=90) | monthly DP  (n=90) | Prevalence of placental malaria = 20-50% | 41-70% |
| 2 | Infants 0-24 months of age born to mothers assigned to receive IPTp with 3 dose DP or monthly DP | Infants assigned q 3 monthly DP (n=90) | Infants assigned monthly DP (n=90) | Incidence of malaria =  3-5 episode PPY | 18-23% |
| 3 | Infants 0-24 months of age randomized to q 3 monthly DP | Infants of mothers assigned 3 dose SP (n=90) | Infants of mothers assigned 3 dose DP or monthly DP (n=45 each) | Incidence of malaria =  3-5 episodes PPY | 22-28% |
|  | Infants 24-36 months of age after chemoprevention stopped | Infants assigned q 3 monthly DP (n=162) | Infants assigned monthly DP (n=81) | Incidence of malaria =  3-5 episodes PPY | 16-21% |

* Relative difference = (estimated outcome in control arm – estimated outcome in the comparison arm) / estimated outcome on the control arm (two-sided alpha = 0.05, power = 80%).

- 1. **Data and Safety Monitoring Plan**

The proposed study will conform to rigorous standard monitoring procedures, standardized reporting of adverse events (Adverse Event Report Forms are completed by study coordinators and sent immediately to the investigators), and regular review of the study by a Data and Safety Monitoring Board (DSMB). The PI has primary responsibility for the overall conduct of the study, including the safety of human subjects. The PI will ensure appropriate (1) conduct of the informed consent process (e.g. that informed consent is obtained before proceeding with study procedures); (2) enrollment of study subjects; (3) collection and analysis of data; (4) implementation of study procedures to ensure consistent monitoring of subjects for possible adverse events; (5) review of adverse events and reporting to the DSMB and the IRBs; and (6) maintenance of the privacy and confidentiality of study subjects. The PI maintains ultimate responsibility for the project and for the safety of study participants. The PI will be in contact with the research team on a regular basis to review the progress of the study and address any human subject issues that occur. These discussions may involve adverse event prevention measures, recruiting of appropriate study subjects, research staff training on protection of human subjects, as well as occurrence of adverse events, unexpected incidents, or protocol problems.

- - 1. **Data and Safety Monitoring Board**

A DSMB will be established by the study team in cooperation with the sponsor to assess at intervals the progress of a clinical trial, safety data, and critical efficacy variables and recommend to the sponsor whether to continue, modify or terminate a trial. The DSMB will have written operating procedures and maintain records of all its meetings, including interim results; these will be available for review when the trial is complete. The DSMB will be a separate entity from the US and host-country Institutional Review Boards (IRBs). The independence of the DSMB is intended to control the sharing of important comparative information and to protect the integrity of the clinical trial from adverse impact resulting from access to trial information. DSMB members will not participate in the study as investigators and will not have conflicts of interest regarding the study or the investigational product. The composition of the DSMB will include at minimum:

DSMB Chair, having experience and expertise in clinical trials

Scientist with expertise in malaria.

Scientist with expertise in pharmacology

Biostatistician with expertise in clinical trials.

A member of the sponsor, NICHD, will be invited to attend and thus have access to unblinded information, and control of dissemination of interim trial results within the sponsor organization. The DSMB will meet at least annually to review progress of the clinical trial and safety data.

The DSMB will review the study for progress and safety. The PI will provide information that will allow the DSMB to review and assess the following:

- The research protocol, informed consent documents and plans for data safety and monitoring;
- Periodic assessments of data quality and timeliness, participant recruitment, accrual and retention, participant risk versus benefit, and other factors that can affect study outcome;
- Factors external to the study when relevant information, such as scientific or therapeutic developments, may have an impact on the safety of the participants or the ethics of the trial;
- Study performance to make recommendations and assist in the resolution of problems;
- The safety of the study participants;
- The safety and scientific progress of the trial;
- The continuation, termination or other modifications of the trial based on the observed beneficial or adverse effects of the treatment under study;
- The confidentiality of the data and the results of monitoring; and
- Any problems with study conduct, enrollment, sample size and/or data collection.

The first meeting of the DSMB will take place prior to the initiation of the study to discuss the protocol and the Data Safety Monitoring Plan. Meetings of the DSMB shall be held according to the plan outlined above. Meetings shall be closed to the public because discussions may address confidential patient data. Meetings may be convened as conference calls as well as in person. An emergency meeting of the Board may be called at any time should questions of patient safety arise. The DSMB may request the presence of study investigators at such meetings.

The study PI will distribute study information to the DSMB at least 10 days prior to a scheduled meeting. The DSMB may request additions and other modifications to this information on a one‑time or continuing basis. This information will consist of two parts: (1) information on study progress such as accrual, baseline characteristics, and other general information on study status and (2) any confidential data on study outcomes, including safety data. A formal report from the DSMB should be supplied to the PI within 6 weeks of each meeting. Each report should conclude with a recommendation to continue or to terminate the study. This recommendation should be made by formal majority vote. A recommendation to terminate the study should be transmitted to the PI, IRBs and NIH as rapidly as possible, by immediate telephone and fax if sufficiently urgent. In the event of a split vote in favor of continuation, a minority report should be contained within the regular DSMB report.

- - 1. **Interim safety analysis**

Over the course of the trial, we will perform an interim safety analysis in addition to a final safety analysis for a total of two sequential evaluations of study safety. Safety will be evaluated separately for pregnant women and infants evaluating the incidence rate ratio of significant adverse events (grade 3/4 & SAEs). The interim safety analysis for pregnant women will be performed when ½ of the study subjects have given birth and for infants 0-24 months of age when ½ of the anticipated total observation time has been accrued. A separate standardized test statistic will be calculated for the adverse event incidence rate ratio for pregnant women and infants. If this statistic exceeds the nominal critical value calculated using the error spending function (Table 11), then a statistically significant result will have been achieved at the time of that analysis. In that event, the sponsor will be notified and a report submitted for review by the Data Safety Monitoring Board (DSMB). In each interim safety analysis, the study team will present information on recruitment and the results of interim safety analyses to the DSMB, which will review the data and recommend a course of action.

| **Table 11. Schedule of interim safety analysis and boundaries to monitor study outcome** | | | | |
| --- | --- | --- | --- | --- |
| **Number of Evaluable Subjects Accrued or % of Total Accrual Time** | **Test Statistic** | | **Alpha** | **Cumulative Alpha** |
|  | **Lower Bound** | **Upper Bound** |  |  |
| N=150 or 50% of accrual time | -2.51 | 2.51 | 0.00601 | 0.01210 |
| N=300 or 100% of accrual time | -1.99 | 1.99 | 0.02313 | 0.05000 |

This analysis assumes α=0.05 (two-sided test), O’Brien-Fleming boundaries (DeMets error-spending function) and 300 trial participants. We will utilize Programs for Computing Group Sequential Boundaries Using the Lan-DeMets Method.

- - 1. **Stopping rules**

The DSMB will determine whether to stop the study for early evidence of intervention safety problems after a thorough review of interim data. Interim reports will provide cumulative enrollment figures and cumulative adverse birth outcomes, serious adverse events (classified according to grade) for both women and children, sorted by study arm. Brief clinical descriptions of key events will also be provided. The PI will be responsible for immediately reporting to the funding agency any temporary or permanent suspension of the project and the reason for the suspension.

1. **DATA COLLECTION AND MONITORING**
   1. **Record Keeping**

All clinical data will be recorded onto standardized case record forms (CRFs) by study clinicians. Blood smear results will be recorded in a laboratory record book by the study laboratory technologists and then transferred to the case record forms by study coordinators, who will review the case record forms frequently for completeness and accuracy. Other laboratory data (CBC, ALT measurements) will be entered into the CRFs and hard copies of the original results will be stored in a file. Data will be entered directly from CRFs into a computerized database or transferred from the CRFs onto standardized data extraction forms and then into a computerized database. All computerized data will be double entered to verify accuracy of entry. Electronic data including all study databases and supporting electronic documentation will be archived to large-scale digital tape on a daily basis. On a monthly basis, a complete backup tape will be transported off-site to the Kampala Data Management Center for rotating secure storage. In addition, the database from the backup will be placed onto one of the Kampala DMC servers as a data mirror for read-only access in the event that the Tororo web-site becomes temporary unavailable.

- 1. **Data Quality Assurance and Monitoring**

In order to insure data quality, the study Data Manager will perform a quarterly data quality audit. For this audit a 1% random sample of study forms entered into the data management system from the previous 2 weeks will be selected and compared for accuracy with the original case-report forms and source documents. In addition the study the Data Manager will perform monthly reviews of the 100% double data entry data verification logs and the data management system audit trail log to identify potential data quality issues**.** The data will be owned by the Makerere University-University of California, San Francisco Research Collaboration.

1. **HUMAN SUBJECTS**
   1. **Subject Selection Criteria**

Study subjects will be HIV-uninfected pregnant women and the children born to them who meet our selection criteria and provide informed consent. We plan to recruit only Ugandan residents and will recruit both pregnant women age 16 and above and male or female children. Enrollment of children is appropriate for study, as malaria is primarily a pediatric disease in highly endemic countries such as Uganda. In addition, pregnant women and young children are groups that have a high risk of malaria and therefore are expected to benefit most from antimalarial chemoprevention.

- 1. **Risks and Discomforts**
     1. **Privacy**

Care will be taken to protect the privacy of subjects, as described in this protocol. However, there is a risk that others may inadvertently see patients’ medical information, and thus their privacy may be compromised.

- - 1. **Finger Pricks, Heel Sticks, and Venipuncture**

Risks of these procedures include pain, transient bleeding and soft-tissue infection.

- - 1. **Risks of Study Medications**

**Risk of Sulfadoxine-Pyrimethamine**

Although technically a combination regimen, SP is generally considered a single antimalarial agent, as its success depends on the synergistic action of its two component inhibitors of folate synthesis. SP is approved in the USA for the treatment of falciparum malaria and for chemoprophylaxis against malaria in travelers, but it is no longer recommended for this second use due to rare, but serious toxicity. Adverse reactions listed on the SP package insert (Roche, USA) are blood dyscrasias (agranulocytosis, aplastic anemia, thrombocytopenia), allergic reactions (erythema multiforme and other dermatological conditions), gastrointestinal reactions (glossitis, stomatitis, nausea, emesis, abdominal pain, hepatitis, diarrhea), central nervous system reactions (headache, peripheral neuritis, convulsions, ataxia, hallucinations), respiratory reactions (pulmonary infiltrates), and miscellaneous reactions (fever, chills, nephrosis); based on widespread experience with the drug, all of these reactions appear to be uncommon or rare with short-term therapeutic use. The best-documented severe adverse effects with SP are cutaneous reactions, primarily noted when SP was used for long-term chemoprophylaxis in non-African populations. Reported rates of serious reactions to SP in the UK, with long-term use for chemoprophylaxis, were 1:2100, with 1:4900 serious dermatological reactions and 1:11,100 deaths.[^81^](#_ENREF_81) Estimated rates of toxicity in the US were 1:5000-8000 severe cutaneous reactions and 1:11,000-25,000 deaths.[^82^](#_ENREF_82) Clinical experience suggests that risks of severe toxicity are much lower with malaria treatment regimens in Africa.

The WHO currently recommends IPTp with SP in areas with moderate-to-high malaria transmission.[^83^](#_ENREF_83) In a recent systematic review and meta-analysis of 7 trials from sub-Saharan Africa, IPTp with 3 or more doses of SP was associated with a higher birth weight and lower risk of low birth weight compared to 2 doses of SP. In addition there were no differences in the rates of serious adverse events between the two groups.[^84^](#_ENREF_84)

**Risk of Dihydroartemisinin-piperaquine**

**Risks associated with DP among adults and children.** Minyt and colleagues conducted a systematic review of DP efficacy and safety for treatment of malaria using data from 14 clinical trials involving adults and children.[^85^](#_ENREF_85) There were 2636 study participants treated with DP in 13 trials in which safety data were reported. Overall, DP was associated with fewer adverse events compared to comparator medications. The most common adverse events were dizziness, nausea and vomiting, though generally the medication was well-tolerated by both adults and children (Table 12). Of note, the only serious adverse events in these 14 studies included 5 deaths (2 adults, 3 children) that were thought unrelated to DP.

### **Table 12. Summary of adverse events following treatment with dihydroartemisinin-piperaquine**

More recently Lwin et al conducted a randomized controlled trial of monthly vs. bimonthly DP IPT among 961 adults at high risk of malaria at the Northwest border of Thailand.[^51^](#_ENREF_51) Overall, 69% of the participants included in the final analysis reported at least one adverse event. There was no difference in the proportion of those reporting at least one adverse event among participants in the monthly vs. bimonthly vs. placebo arms. There was an increased risk of joint pain among participants randomized to the placebo arm, but otherwise there was no differences noted in adverse events by study arm. There was only one serious adverse event not related to the use of DP (Table 13).

**Table 13. Frequency, incidence, and risk of the 20 most frequently reported adverse events**

**Risks associated the use of DP for IPT in infants.** Relevant to this protocol is the PROMOTE Chemoprevention study, an open label randomized clinical trial being conducted by our group evaluating the protective efficacy of 3 different chemoprevention regimens against malaria compared to the current standard of care of no chemoprevention. This is the first study to evaluate the safety of DP when used for IPT in infants. Final results have been generated and show DP to be very well-tolerated and associated with a significantly lower rate of all grade 3-4 adverse events, elevated temperature, anemia, and thrombocytopenia compared to the control arm (Table 14)(unpublished data). In addition, 145 ECGs performed 19 study participants randomized to DP have documented QTc intervals to be within normal limits.

### **Table 14. Comparison of adverse events among children between 6-24 months of age**

| **Characteristic** | **Number of events (incidence per PYAR) by treatment arm** | | | |
| --- | --- | --- | --- | --- |
|  | **Control** | **Monthly SP** | **Daily TS** | **Monthly DP** |
| All grade 3-4 adverse events | 169 (1·159) | 202 (1·415) | 135 (0·914) | 87 (0·611) ^‡^ |
| All serious adverse events | 26 (0·178) | 52 (0·364) | 29 (0·196) | 13 (0·091) |
| Grade 3-4 adverse events possibly related to study drugs | N/A | 8 (0·056) | 8 (0·054) | 3 (0·021) |
| Individual grade 3-4 adverse events*  Elevated temperature  Anaemia  Thrombocytopenia  Elevated aspartate aminotransferase  Elevated alanine aminotransferase  Neutropenia | 79 (0·542)  56 (0·384)  18 (0·123)  7 (0·048)  4 (0·027)  3 (0·021) | 78 (0·546)  86 (0·602)  17 (0·119)  8 (0·056)  4 (0·028)  6 (0·042) | 58 (0·393)  47 (0·318)  9 (0·061)  6 (0·041)  4 (0·027)  2 (0·014) | 46 (0·323) ^†^  24 (0·168) ^†^  5 (0·035)^α^  3 (0·021)  3 (0·021)  1 (0·007) |

PYAR = person-years at risk.

SP = sulfadoxine-pyrimethamine. TS = trimethoprim-sulfamethoxazole. DP = dihydroartemisinin-piperaquine.

* Only includes those with at least 5 total events.

^α^ p-value < 0.05 compared to control group; ^†^ p-value < 0.01 compared to control group; ^‡^ p-value < 0.001 compared to control group.

**Risks associated with DP during pregnancy.** While data are limited, preclinical animal studies [^86-88^](#_ENREF_86) and clinical studies involving pregnant women have not demonstrated significant safety concerns with the use of DP. Given the devastating effects of malaria during pregnancy and the suboptimal preventive measures of ITNs and IPTp with SP, the potential benefit of DP during pregnancy is tremendous. Other than IPTp with SP and ITNs, there are no other viable, sustainable antimalarial preventive measures that have proven successful at addressing malaria during pregnancy. While there are no known teratogenic risks of DP, women will undergo ultrasound at screening to ensure that there is no first trimester exposure to DP. If it is launched prior to study implementation or while the study is underway, we will enroll study participants in the Antimalarial Pregnancy Exposure Registry.

- 1. **Treatment and Compensation for Injury**

If the participant is injured as a result of being in this study, treatment will be available through Tororo District Hospital. The costs of the treatment may be covered by the study sponsor, NICHD, depending on a number of factors. Makerere University, UCSF, and NICHD do not normally provide any other form of compensation for injury.

- 1. **Costs to the Subjects**

There will be no cost to the participant or their parents/guardians for participation in this study.

- 1. **Reimbursement of Subjects**

Participants will not be paid for their participation in the study. The study will provide all routine medical care, including evaluations, medications available in our clinic, and cost of any transportation free of charge. In addition, we will reimburse the cost of consultation for referrals made by study physicians to other clinics and services within Tororo District Hospital and visits the cost of most diagnostic tests (including laboratory test, X-rays, and ultrasounds) and medications resulting from referrals by the study team, using available funds. However, reimbursement of all diagnostic tests and treatment recommended outside the study clinic cannot be guaranteed in all circumstances.

- 1. **Institutional Review Board (IRB) Review and Informed Consent**

This protocol, all procedures and consent forms, and any subsequent modifications must be reviewed and approved by the IRBs of all the participating institutions in both the U.S. and in Uganda. This includes the UCSF Committee on Human Research (CHR), the MU School of Medicine - Research and Ethics Committee (SOM-REC) or MU School of Biomedical Sciences – Research and Ethics Committee (SBS-REC), and the Uganda National Council of Science and Technology (UNCST).

All consent forms will be translated into the local languages (Dopadhola, Ateso, Swahili, Luganda, and English) and back-translated into English to ensure correct use of language. Consent forms will be read aloud to parents by trained study interviewers. The informed consent will describe the purpose of the study, all the procedures involved, and the risks and benefits of participation. Study physicians will ask parents/guardians of study participants to summarize the study and explain the reasons why they want to participate. Either a signature or a thumbprint (for parents/guardians who cannot read) will be acceptable to confirm informed consent for participation in the study.

**11.7 Definition of Parent/Guardianship.**

For this study, we will define a parent/primary guardian of the child enrolled in the study as the women giving birth to the child. However to the extent possible, consent of the father will be also obtained.

**12. PUBLICATION OF RESEARCH FINDINGS**

The findings from this study may be published in a medical journal. No individual identities will be used in any reports or publications resulting from the study. The researchers will publish results of the trial in accordance with NICHD, UNCST, UCSF, and Makerere University guidelines.

**13. BIOHAZARD CONTAINMENT**

As the transmission of HIV and other blood-borne pathogens can occur through contact with contaminated needles, blood, and blood products, appropriate blood and secretion precautions will be employed by all personnel involved in the drawing of blood, exposure to blood and secretions, and shipping and handling of all specimens for this study. We will follow the current guidelines set forth by the Centers for Disease Control and Prevention and the NIH. All infectious specimens will be transported using packaging mandated in the Federal Code of Regulations, CDC 42 CFR Part 72.

**14. REFERENCES**

1. Desai M, ter Kuile FO, Nosten F, et al. Epidemiology and burden of malaria in pregnancy. *Lancet Infect Dis* 2007; **7**(2): 93-104.

2. Guyatt HL, Snow RW. The epidemiology and burden of Plasmodium falciparum-related anemia among pregnant women in sub-Saharan Africa. *Am J Trop Med Hyg* 2001; **64**(1-2 Suppl): 36-44.

3. Steketee RW, Nahlen BL, Parise ME, Menendez C. The burden of malaria in pregnancy in malaria-endemic areas. *Am J Trop Med Hyg* 2001; **64**(1-2 Suppl): 28-35.

4. Garner P, Brabin B. A review of randomized controlled trials of routine antimalarial drug prophylaxis during pregnancy in endemic malarious areas. *Bull World Health Org* 1994; **72**(1): 89-99.

5. Kayentao K, Kodio M, Newman RD, et al. Comparison of intermittent preventive treatment with chemoprophylaxis for the prevention of malaria during pregnancy in Mali. *J Infect Dis* 2005; **191**(1): 109-16.

6. Schultz LJ, Steketee RW, Macheso A, Kazembe P, Chitsulo L, Wirima JJ. The efficacy of antimalarial regimens containing sulfadoxine-pyrimethamine and/or chloroquine in preventing peripheral and placental Plasmodium falciparum infection among pregnant women in Malawi. *Am J Trop Med Hyg* 1994; **51**(5): 515-22.

7. Shulman CE, Dorman EK, Cutts F, et al. Intermittent sulphadoxine-pyrimethamine to prevent severe anaemia secondary to malaria in pregnancy: a randomised placebo-controlled trial. *Lancet* 1999; **353**(9153): 632-6.

8. Verhoeff FH, Brabin BJ, Chimsuku L, Kazembe P, Russell WB, Broadhead RL. An evaluation of the effects of intermittent sulfadoxine-pyrimethamine treatment in pregnancy on parasite clearance and risk of low birthweight in rural Malawi. *Ann Trop Med Parasitol* 1998; **92**(2): 141-50.

9. ter Kuile FO, van Eijk AM, Filler SJ. Effect of sulfadoxine-pyrimethamine resistance on the efficacy of intermittent preventive therapy for malaria control during pregnancy: a systematic review. *JAMA* 2007; **297**(23): 2603-16.

10. Gesase S, Gosling RD, Hashim R, et al. High resistance of Plasmodium falciparum to sulphadoxine/pyrimethamine in northern Tanzania and the emergence of dhps resistance mutation at Codon 581. *PLoS ONE* 2009; **4**(2): e4569.

11. Nankabirwa J, Cundill B, Clarke S, et al. Efficacy, safety, and tolerability of three regimens for prevention of malaria: a randomized, placebo-controlled trial in Ugandan schoolchildren. *PLoS ONE* 2010; **5**(10): e13438.

12. Harrington WE, Mutabingwa TK, Muehlenbachs A, et al. Competitive facilitation of drug-resistant Plasmodium falciparum malaria parasites in pregnant women who receive preventive treatment. *Proc Natl Acad Sci U S A* 2009; **106**(22): 9027-32.

13. Sandison TG, Homsy J, Arinaitwe E, et al. Protective efficacy of co-trimoxazole prophylaxis against malaria in HIV exposed children in rural Uganda: a randomised clinical trial. *BMJ* 2011; **342**: d1617.

14. Harrington WE, Mutabingwa TK, Kabyemela E, Fried M, Duffy PE. Intermittent treatment to prevent pregnancy malaria does not confer benefit in an area of widespread drug resistance. *Clin Inf Dis* 2011; **53**(3): 224-30.

15. Ndyomugyenyi R, Clarke SE, Hutchison CL, Hansen KS, Magnussen P. Efficacy of malaria prevention during pregnancy in an area of low and unstable transmission: an individually-randomised placebo-controlled trial using intermittent preventive treatment and insecticide-treated nets in the Kabale Highlands, southwestern Uganda. *Trans Roy Soc Trop Med Hyg* 2011; **105**(11): 607-16.

16. McGready R, White NJ, Nosten F. Parasitological efficacy of antimalarials in the treatment and prevention of falciparum malaria in pregnancy 1998 to 2009: a systematic review. *BJOG* 2011; **118**(2): 123-35.

17. Adam I, Ali DM, Abdalla MA. Artesunate plus sulfadoxine-pyrimethamine in the treatment of uncomplicated Plasmodium falciparum malaria during pregnancy in eastern Sudan. *Trans Roy Soc Trop Med Hyg* 2006; **100**(7): 632-5.

18. Deen JL, von Seidlein L, Pinder M, Walraven GE, Greenwood BM. The safety of the combination artesunate and pyrimethamine-sulfadoxine given during pregnancy. *Trans Roy Soc Trop Med Hyg* 2001; **95**(4): 424-8.

19. Manyando C, Mkandawire R, Puma L, et al. Safety of artemether-lumefantrine in pregnant women with malaria: results of a prospective cohort study in Zambia. *Malar J* 2010; **9**: 249.

20. Piola P, Nabasumba C, Turyakira E, et al. Efficacy and safety of artemether-lumefantrine compared with quinine in pregnant women with uncomplicated Plasmodium falciparum malaria: an open-label, randomised, non-inferiority trial. *Lancet Infec Dis* 2010; **10**(11): 762-9.

21. Poespoprodjo JR, Fobia W, Kenangalem E, et al. Highly effective therapy for maternal malaria associated with a lower risk of vertical transmission. *J Infec Dis* 2011; **204**(10): 1613-9.

22. Rijken MJ, McGready R, Boel ME, et al. Dihydroartemisinin-piperaquine rescue treatment of multidrug-resistant Plasmodium falciparum malaria in pregnancy: a preliminary report. *Am J Trop Med Hyg* 2008; **78**(4): 543-5.

23. Rijken MJ, McGready R, Phyo AP, et al. Pharmacokinetics of dihydroartemisinin and piperaquine in pregnant and nonpregnant women with uncomplicated falciparum malaria. *Antimicrob Agents Chemo* 2011; **55**(12): 5500-6.

24. Tarning J, Rijken MJ, McGready R, et al. Population pharmacokinetics of dihydroartemisinin and piperaquine in pregnant and nonpregnant women with uncomplicated malaria. *Antimicrob Agents Chemo* 2012; **56**(4): 1997-2007.

25. World Heatlh Organization. Guidelines for the treatment of malaria, 2010.

26. Rogier C. Natural history of Plasmodium falciparum malaria and determining factors of the acquisition of antimalaria immunity in two endemic areas, Dielmo and Ndiop (Senegal). *Bulletin et memoires de l'Academie royale de medecine de Belgique* 2000; **155**(5-6): 218-26.

27. White NJ. Intermittent presumptive treatment for malaria. *PLoS Med* 2005; **2**(1): e3.

28. Snow RW, Craig M, Deichmann U, Marsh K. Estimating mortality, morbidity and disability due to malaria among Africa's non-pregnant population. *Bull World Health Organ* 1999; **77**(8): 624-40.

29. Rogier C, Tall A, Diagne N, Fontenille D, Spiegel A, Trape JF. Plasmodium falciparum clinical malaria: lessons from longitudinal studies in Senegal. *Parassitologia* 1999; **41**(1-3): 255-9.

30. Ramphul U, Boase T, Bass C, Okedi LM, Donnelly MJ, Muller P. Insecticide resistance and its association with target-site mutations in natural populations of Anopheles gambiae from eastern Uganda. *Trans Roy Soc Trop Med Hyg* 2009; **103**(11): 1121-6.

31. Verhaeghen K, Bortel WV, Roelants P, Okello PE, Talisuna A, Coosemans M. Spatio-temporal patterns in kdr frequency in permethrin and DDT resistant Anopheles gambiae s.s. from Uganda. *Am J Trop Med Hyg* 2010; **82**(4): 566-73.

32. Lengeler C. Insecticide-treated bed nets and curtains for preventing malaria. *Cochrane database of systematic reviews* 2004; (2): CD000363.

33. Trape JF, Tall A, Diagne N, et al. Malaria morbidity and pyrethroid resistance after the introduction of insecticide-treated bednets and artemisinin-based combination therapies: a longitudinal study. *Lancet Infec Dis* 2011; **11**(12): 925-32.

34. Jagannathan P, Muhindo MK, Kakuru A, et al. Increasing incidence of malaria in children despite insecticide-treated bed nets and prompt anti-malarial therapy in Tororo, Uganda. *Malar J* 2012; **11**: 435.

35. Aponte JJ, Schellenberg D, Egan A, et al. Efficacy and safety of intermittent preventive treatment with sulfadoxine-pyrimethamine for malaria in African infants: a pooled analysis of six randomised, placebo-controlled trials. *Lancet* 2009; **374**(9700): 1533-42.

36. Chandramohan D, Owusu-Agyei S, Carneiro I, et al. Cluster randomised trial of intermittent preventive treatment for malaria in infants in area of high, seasonal transmission in Ghana. *BMJ* 2005; **331**(7519): 727-33.

37. Kobbe R, Kreuzberg C, Adjei S, et al. A randomized controlled trial of extended intermittent preventive antimalarial treatment in infants. *Clin Infect Dis* 2007; **45**(1): 16-25.

38. Macete E, Aide P, Aponte JJ, et al. Intermittent preventive treatment for malaria control administered at the time of routine vaccinations in mozambican infants: a randomized, placebo-controlled trial. *J Infect Dis* 2006; **194**(3): 276-85.

39. Mockenhaupt FP, Reither K, Zanger P, et al. Intermittent preventive treatment in infants as a means of malaria control: a randomized, double-blind, placebo-controlled trial in northern Ghana. *Antimicrob Agents Chemother* 2007; **51**(9): 3273-81.

40. Schellenberg D, Menendez C, Kahigwa E, et al. Intermittent treatment for malaria and anaemia control at time of routine vaccinations in Tanzanian infants: a randomised, placebo-controlled trial. *Lancet* 2001; **357**(9267): 1471-7.

41. Odhiambo FO, Hamel MJ, Williamson J, et al. Intermittent preventive treatment in infants for the prevention of malaria in rural Western kenya: a randomized, double-blind placebo-controlled trial. *PLoS ONE* 2010; **5**(4): e10016.

42. Dicko A, Diallo AI, Tembine I, et al. Intermittent preventive treatment of malaria provides substantial protection against malaria in children already protected by an insecticide-treated bednet in Mali: a randomised, double-blind, placebo-controlled trial. *PLoS Med* 2011; **8**(2): e1000407.

43. Konate AT, Yaro JB, Ouedraogo AZ, et al. Intermittent preventive treatment of malaria provides substantial protection against malaria in children already protected by an insecticide-treated bednet in Burkina Faso: a randomised, double-blind, placebo-controlled trial. *PLoS Med* 2011; **8**(2): e1000408.

44. Wilson AL. A systematic review and meta-analysis of the efficacy and safety of intermittent preventive treatment of malaria in children (IPTc). *PLoS ONE* 2011; **6**(2): e16976.

45. Inaugural meeting of the malaria policy advisory committee to the WHO: conclusions and recommendations. *Malar J* 2012; **11**: 137.

46. World Health Organization. World Malaria Report 2011, 2011.

47. ABC Study Group. A head-to-head comparison of four artemisinin-based combinations for treating uncomplicated malaria in African children: a randomized trial. *PLoS Med* 2011; **8**(11): e1001119.

48. Arinaitwe E, Sandison TG, Wanzira H, et al. Artemether-lumefantrine versus dihydroartemisinin-piperaquine for falciparum malaria: a longitudinal, randomized trial in young Ugandan children. *Clin Infect Dis* 2009; **49**(11): 1629-37.

49. Kamya MR, Yeka A, Bukirwa H, et al. Artemether-lumefantrine versus dihydroartemisinin-piperaquine for treatment of malaria: a randomized trial. *PLoS Clinical Trials* 2007; **2**(5): e20.

50. Yeka A, Dorsey G, Kamya MR, et al. Artemether-lumefantrine versus dihydroartemisinin-piperaquine for treating uncomplicated malaria: a randomized trial to guide policy in Uganda. *PLoS ONE* 2008; **3**(6): e2390.

51. Lwin KM, Phyo AP, Tarning J, et al. Randomized, double-blind, placebo-controlled trial of monthly versus bimonthly dihydroartemisinin-piperaquine chemoprevention in adults at high risk of malaria. *Antimicrob Agents Chemother* 2012; **56**(3): 1571-7.

52. Dauby N, Goetghebuer T, Kollmann TR, Levy J, Marchant A. Uninfected but not unaffected: chronic maternal infections during pregnancy, fetal immunity, and susceptibility to postnatal infections. *Lancet Infec Dis* 2012; **12**(4): 330-40.

53. Adegnika AA, Kohler C, Agnandji ST, et al. Pregnancy-associated malaria affects toll-like receptor ligand-induced cytokine responses in cord blood. *J Infect Dis* 2008; **198**(6): 928-36.

54. Ismaili J, van der Sande M, Holland MJ, et al. Plasmodium falciparum infection of the placenta affects newborn immune responses. *Clin Exp Immunol* 2003; **133**(3): 414-21.

55. Malhotra I, Mungai P, Muchiri E, et al. Distinct Th1- and Th2-Type prenatal cytokine responses to Plasmodium falciparum erythrocyte invasion ligands. *Infect Immun* 2005; **73**(6): 3462-70.

56. Metenou S, Suguitan AL, Jr., Long C, Leke RG, Taylor DW. Fetal immune responses to Plasmodium falciparum antigens in a malaria-endemic region of Cameroon. *J Immunol* 2007; **178**(5): 2770-7.

57. Bardaji A, Sigauque B, Sanz S, et al. Impact of malaria at the end of pregnancy on infant mortality and morbidity. *J Infect Dis* 2011; **203**(5): 691-9.

58. Le Hesran JY, Cot M, Personne P, et al. Maternal placental infection with Plasmodium falciparum and malaria morbidity during the first 2 years of life. *Am J Epidemiol* 1997; **146**(10): 826-31.

59. Malhotra I, Dent A, Mungai P, et al. Can prenatal malaria exposure produce an immune tolerant phenotype? A prospective birth cohort study in Kenya. *PLoS Med* 2009; **6**(7): e1000116.

60. Mutabingwa TK, Bolla MC, Li JL, et al. Maternal malaria and gravidity interact to modify infant susceptibility to malaria. *PLoS Med* 2005; **2**(12): e407.

61. Schwarz NG, Adegnika AA, Breitling LP, et al. Placental malaria increases malaria risk in the first 30 months of life. *Clin Infect Dis* 2008; **47**(8): 1017-25.

62. Greenwood BM, David PH, Otoo-Forbes LN, et al. Mortality and morbidity from malaria after stopping malaria chemoprophylaxis. *Trans R Soc Trop Med Hyg* 1995; **89**(6): 629-33.

63. Menendez C, Kahigwa E, Hirt R, et al. Randomised placebo-controlled trial of iron supplementation and malaria chemoprophylaxis for prevention of severe anaemia and malaria in Tanzanian infants. *Lancet* 1997; **350**(9081): 844-50.

64. Belnoue E, Costa FT, Frankenberg T, et al. Protective T cell immunity against malaria liver stage after vaccination with live sporozoites under chloroquine treatment. *J Immunol* 2004; **172**(4): 2487-95.

65. Belnoue E, Voza T, Costa FT, et al. Vaccination with live Plasmodium yoelii blood stage parasites under chloroquine cover induces cross-stage immunity against malaria liver stage. *J Immunol* 2008; **181**(12): 8552-8.

66. Friesen J, Silvie O, Putrianti ED, Hafalla JC, Matuschewski K, Borrmann S. Natural immunization against malaria: causal prophylaxis with antibiotics. *Sci Transl Med* 2010; **2**(40): 40ra9.

67. Roestenberg M, McCall M, Hopman J, et al. Protection against a malaria challenge by sporozoite inoculation. *New Engl J Med* 2009; **361**(5): 468-77.

68. Roestenberg M, Teirlinck AC, McCall MB, et al. Long-term protection against malaria after experimental sporozoite inoculation: an open-label follow-up study. *Lancet* 2011; **377**(9779): 1770-6.

69. Dorsey G, Dokomajilar C, Kiggundu M, Staedke SG, Kamya MR, Rosenthal PJ. Principal role of dihydropteroate synthase mutations in mediating resistance to sulfadoxine-pyrimethamine in single-drug and combination therapy of uncomplicated malaria in Uganda. *Am J Trop Med Hyg* 2004; **71**(6): 758-63.

70. Kyabayinze D, Cattamanchi A, Kamya MR, Rosenthal PJ, Dorsey G. Validation of a simplified method for using molecular markers to predict sulfadoxine-pyrimethamine treatment failure in African children with falciparum malaria. *Am J Trop Med Hyg* 2003; **69**(3): 247-52.

71. Kamya MR, Bakyaita NN, Talisuna AO, Were WM, Staedke SG. Increasing antimalarial drug resistance in Uganda and revision of the national drug policy. *Trop Med Int Health* 2002; **7**(12): 1031-41.

72. Kamya MR, Gasasira AF, Achan J, et al. Effects of trimethoprim-sulfamethoxazole and insecticide-treated bednets on malaria among HIV-infected Ugandan children. *AIDS* 2007; **21**(15): 2059-66.

73. Sibley CH, Hyde JE, Sims PF, et al. Pyrimethamine-sulfadoxine resistance in Plasmodium falciparum: what next? *Trends Parasitol* 2001; **17**(12): 582-8.

74. Kamya MR, Yeka A, Bukirwa H, et al. Artemether-lumefantrine versus dihydroartemisinin-piperaquine for treatment of malaria: a randomized trial. *PLoS Clin Trials* 2007; **2**(5): e20.

75. Katrak S, Gasasira A, Arinaitwe E, et al. Safety and tolerability of artemether-lumefantrine versus dihydroartemisinin-piperaquine for malaria in young HIV-infected and uninfected children. *Malar J* 2009; **8**: 272.

76. Murray CJ, Rosenfeld LC, Lim SS, et al. Global malaria mortality between 1980 and 2010: a systematic analysis. *Lancet* 2012; **379**(9814): 413-31.

77. Menendez C, Bardaji A, Sigauque B, et al. A randomized placebo-controlled trial of intermittent preventive treatment in pregnant women in the context of insecticide treated nets delivered through the antenatal clinic. *PLoS ONE* 2008; **3**(4): e1934.

78. Cisse B, Cairns M, Faye E, et al. Randomized trial of piperaquine with sulfadoxine-pyrimethamine or dihydroartemisinin for malaria intermittent preventive treatment in children. *PLoS ONE* 2009; **4**(9): e7164.

79. Cisse B, Sokhna C, Boulanger D, et al. Seasonal intermittent preventive treatment with artesunate and sulfadoxine-pyrimethamine for prevention of malaria in Senegalese children: a randomised, placebo-controlled, double-blind trial. *Lancet* 2006; **367**(9511): 659-67.

80. Rogerson SJ, Hviid L, Duffy PE, Leke RF, Taylor DW. Malaria in pregnancy: pathogenesis and immunity. *Lancet Infect Dis* 2007; **7**(2): 105-17.

81. Phillips-Howard PA, West LJ. Serious adverse drug reactions to pyrimethamine-sulphadoxine, pyrimethamine-dapsone and to amodiaquine in Britain. *J Roy Soc Med* 1990; **83**(2): 82-5.

82. Miller KD, Lobel HO, Satriale RF, Kuritsky JN, Stern R, Campbell CC. Severe cutaneous reactions among American travelers using pyrimethamine-sulfadoxine (Fansidar) for malaria prophylaxis. *Am J Trop Med Hyg* 1986; **35**(3): 451-8.

83. World Health Organization. Updated WHO Policy Recommendation (October 2012). Intermittent Preventive Treatment of malaria in pregnancy using Sulfadoxine-Pyrimethamine (IPTp-SP). Geneva, 2012.

84. Kayentao K, Garner P, van Eijk AM, et al. Intermittent preventive therapy for malaria during pregnancy using 2 vs 3 or more doses of sulfadoxine-pyrimethamine and risk of low birth weight in Africa: systematic review and meta-analysis. *JAMA* 2013; **309**(6): 594-604.

85. Myint HY, Ashley EA, Day NP, Nosten F, White NJ. Efficacy and safety of dihydroartemisinin-piperaquine. *Trans R Soc Trop Med Hyg* 2007; **101**(9): 858-66.

86. Longo M, Zanoncelli S, Della Torre P, et al. Investigations of the effects of the antimalarial drug dihydroartemisinin (DHA) using the Frog Embryo Teratogenesis Assay-Xenopus (FETAX). *Reprod Toxicol* 2008; **25**(4): 433-41.

87. Longo M, Zanoncelli S, Manera D, et al. Effects of the antimalarial drug dihydroartemisinin (DHA) on rat embryos in vitro. *Reprod Toxicol* 2006; **21**(1): 83-93.

88. Longo M, Zanoncelli S, Torre PD, et al. In vivo and in vitro investigations of the effects of the antimalarial drug dihydroartemisinin (DHA) on rat embryos. *Reprod Toxicol* 2006; **22**(4): 797-810.

**15. APPENDICES**

**Appendix A****. Information Sheet**

**A STUDY ON PREVENTING MALARIA IN PREGNANT WOMEN AND YOUNG CHILDREN**

Makerere University in Uganda and the University of California, San Francisco in the United States are combining efforts in Tororo to study new ways of using malaria drugs to prevent malaria in pregnant women and their babies.

Malaria during pregnancy can have a harmful effect on you or your child. We want to study 2 different malaria drugs to see if they can be used to prevent malaria if taken during pregnancy.

Our study clinic is located at Tororo District Hospital next to the antenatal clinic and is open every day from 8:00 am to 5:00 pm

We want to enroll pregnant women who are at least 16 years old and follow them during pregnancy.

We will then follow the child born up to 3 years of age. We want to study the same 2 different malaria drugs to see if they can be used to prevent malaria in young children.

Women and children in this study will receive free medical care

We shall also give reimbursement for transport to and from our study clinic

For more information, please come to our study clinic where our doctors will be happy to talk to you and see if you and your baby can be in the study.

## Appendix B. Determination of Gestational Age

Gestational age will be based on the first day of the last menstrual period (LMP) and the earliest available ultrasound performed at > 6 weeks gestation. The estimated due date (EDD) is calculated as 280 days following the LMP.

If the first available ultrasound is consistent with a gestational age of 6 to 12 weeks, and the ultrasound gestational age is within 7 days of that given LMP, then the LMP is used to determine gestational age. However, if the ultrasound gestational age differs from the LMP gestational age by more than 7 days, then the ultrasound is used to determine gestational age.

If the first available ultrasound is consistent with a gestational age of 13-24 weeks, and the ultrasound gestational age is within 14 days of that given by the LMP, then the LMP is used to determine gestational age. However, if the ultrasound gestational age differs from the LMP by more than 14 days, then the ultrasound is used to determine gestational age.

If the first available ultrasound is consistent with a gestational age of 25 weeks or more, and the ultrasound gestational age is within 21 days of that given by the LMP, then the LMP is used to determine gestational age. However, if the ultrasound gestational age differs from the LMP by more than 21 days, then the ultrasound is used to determine gestational age. Care here should be taken to rule out intrauterine growth restriction (IUGR).

If the LMP is not known, then the earliest ultrasound performed at > 6 weeks should be used to determine gestational age.

**Appendix C. Household survey**

The household survey will be administered through a completely paperless QDS software system using hand-held tablet computers. A list of questions that will be used in the survey is provided below.

| **Ques. No.** | **Variable Name** | **Question** | |
| --- | --- | --- | --- |
| **Section 1: Identification** | | | |
| 1 | VISDATE | Date of final visit | |
| 2 | STARTIME | Start time of interview | |
| 4 | BC | Birth Cohort Number | |
| 3 | PPTIDA | Study Participant ID | |
| 5 | PPTID | To ensure data integrity, please re-enter the Participant ID. | |
| 7 | INTNUM | Interviewer number | |
| 8 | AGREE | Are you going to conduct the interview with this household? | |
| **Section 2: Household Characteristics** | | | |
| 9 | SWATER | What is the main source of drinking water for members of your household? | |
| 10 | OTHERSCS | Specify other source of water | |
| 11 | TFACLTY | What kind of toilet facility do members of your household usually use? | |
| 12 | OTHERFCY | Specify other kind of toilet facilities | |
| 13 | ELECTRIC | Does your household have...  ...Electricity? | |
| 14 | RADIO | ...Radio? | |
| 15 | CASSETTE | …Cassette player? | |
| 16 | TV | …Television? | |
| 17 | MOBILE | …Mobile phone? | |
| 18 | PHONE | …Fixed phone? | |
| 19 | FRIDGE | …Refrigerator? | |
| 20 | TABLE | …Table? | |
| 21 | CHAIR | …Chairs? | |
| 22 | SOFA | …Sofa set? | |
| 23 | BED | …Bed? | |
| 24 | CUPBOARD | …Cupboard? | |
| 25 | CLOCK | …Clock? | |
| 26 | FUELTYPE | What type of fuel does your household mainly use for cooking? | |
| 27 | OTHERFUE | Specify other type of fuel used | |
| 28 | SENERGY | What is the main source of energy for lighting in the household? | |
| 29 | OTHERENG | Specify other source of energy for lighting | |
| 30 | MMFLOOR | MAIN MATERIAL OF THE FLOOR     RECORD OBSERVATION.    MARK ONLY ONE. | |
| 31 | OTHERMMF | Specify other material of the floor | |
| 32 | MMROOF | MAIN MATERIAL OF THE ROOF.    RECORD OBSERVATION.    MARK ONLY ONE. | |
| 33 | OTHERMMR | Specify other material of the roof | |
| 34 | MMEWALLS | MAIN MATERIAL OF THE EXTERIOR WALLS.    RECORD OBSERVATION.   MARK ONLY ONE. | |
| 35 | OTHERMME | Specify other material of the exterior walls | |
| 36 | HHROOMS | How many rooms in your household are used for sleeping?  (INCLUDING ROOMS OUTSIDE THE MAIN DWELLING)  If there are 15 or more rooms, enter 15 | |
| 37 | HHSPACES | How many sleeping spaces like mats, mattresses, or beds are available in your household?  If there are 25 or more sleeping places, enter 25 | |
| 38 | WATCH | Does any member of your household own or have…   …A watch? | |
| 39 | BICYCLE | ….A bicycle? | |
| 40 | SCOOTER | …A motorcycle or motor scooter? | |
| 41 | CART | …An animal-drawn cart? | |
| 42 | CAR | …A car or truck? | |
| 43 | MBOAT | …A boat with a motor? | |
| 44 | NOMBOAT | …A boat without a motor? | |
| 45 | BANKACCO | …A bank account? | |
| 46 | NUMALAND | How many acres of agricultural land do members of this household own? | |
| 47 | DMARKT | How far is it to the nearest market place? | |
| 48 | HHMEALS | Now I would like to ask you about the food your household eats. How many meals does your household usually have per day? | |
| 49 | HHNUMT | In the past week, on how many days did the household eat meat? | |
| 50 | HHPSF | How often in the last year did you have problems in satisfying the food needs of the household? | |
| 51 | DHFCTY | How far is it to the nearest health facility? | |
| 52 | MTHFCTY | If you were to go this facility,how would you most likely go there? | |
| 53 | OTHERMTH | Specify other means of transport to the health facility | |
| 54 | PSPRAY | At any time in the past 12 months, has anyone asked permission to come into your dwelling to spray the interior walls against mosquitoes? | |
| 55 | GPSPRAY | Did you grant them permission to spray the interior walls of your dwelling? | |
| 56 | RGSPRAY | What was the primary reason that you did not grant permission to spray the interior walls of your dwelling against mosquitoes? | |
| 57 | OTHERRGS | Specify other reasons for not granting permission to spray the interior walls of your dwelling against mosquitoes | |
| 58 | TSPRAY | How many months ago was the dwelling last sprayed? | |
| 59 | WSPRAY | Who sprayed the dwelling? | |
| 60 | OTHERWSP | Specify other people who sprayed the dwelling | |
| 61 | DSPRAY | Did you pay for the dwelling to be sprayed? | |
| 62 | PPWALLS | Since the spraying, have the walls in your dwelling been plastered or painted? | |
| 63 | TPPWALLS | How many months ago were the walls plastered or painted? | |
| 64 | MSPRAY | In the past 12 months, have you seen or heard any messages about spraying the interior walls of your dwelling against mosquitoes? | |
| 65 | MSGA | Where did you hear or see message(s)?  ...Radio? | |
| 66 | MSGB | ...TV? | |
| 67 | MSGC | ...Newspaper/Leaflet? | |
| 68 | MSGD | …Health worker/CMD? | |
| 69 | MSGE | …Neighbour/Relative/Friend | |
| 70 | MSGF | ...Community Leader? | |
| 71 | MSGG | …Village public adress system | |
| 72 | MSGH | ...Don't know | |
| 73 | MSGI | …Other | |
| 74 | OTHERMSG | Specify other | |
| 75 | AHWKER | Is there a community health worker (community medicine distributor/CMD, village health team/VHT, community own resource person/CORP) who distributes malaria medicines in your village or community? | |
| 76 | AMCHWKER | Does the community health worker currently have malaria medicines available? | |
| **Section 3: Study Participants Sleeping Area Characteristics** All questions in this section will be repeated with variable names entopen1, entcov1 , etc. for each entryway and window in the study participants room | | | |
| 77 | SRENTRY | OBSERVATION: How many entryways into the room are there? | |
| 78 | ENTOPN1 | OBSERVATION: Does it open to the outside? | |
| 79 | ENTCOV1 | OBSERVATION: Is the entry way covered? | |
| 80 | ETMM1 | OBSERVATION: Main material is the covering made of. | |
| 81 | OTHCOV1 | Specify Other covering type | |
| 82 | SRWINDOW | OBSERVATION: How many windows are in the room? | |
| 83 | WNDCOV1 | OBSERVATION: Is the window covered? | |
| 84 | WNDOPN1 | OBSERVATION: Does the window open to the outside | |
| 85 | SREAVES | OBSERVATION: Does the room have eaves? | |
| 86 | EAVESCOV | OBSERVATION: If room has eaves, are the eaves covered? | |
| 87 | EAVESOPN | OBSERVATION: Do the eaves open to the outside? | |
| 88 | AIRBRICK | OBSERVATION: Does the room have airbricks? | |
| 89 | AIRBRCOV | OBSERVATION: If the room has airbricks, are the airbricks covered? | |
| 90 | AIRBROPN | OBSERVATION: Do the airbricks open to the outside? | |
| 91 | AIRBRNUM | OBSERVATION: How many airbricks are in the room? | |
| 92 | SLEEP | Where does the study participant usually sleep? | |
| 93 | OTHERSL | Specify other sleeping area | |
| 94 | SRSLNUM | Usually, how many people sleep in the same room as the study participant (excluding the study participant)? | |
| 95 | SRSLNUM5 | How many of those people are under 5 years old (excluding the study participant)? | |
| 96 | SLAREAS | How many sleeping areas are in the room where the study participant sleeps? | |
| 97 | SASLNUM | How many people sleep in the same bed/sleeping area as the study participant under the mosquito net (excluding the study participant)? | |
| 98 | SASLNUM5 | How many of those people are under 5 years old (excluding the study participant)? | |
| **Section 4: Bednets** All questions in this section will be repeated with variable names obs2,mnths2,where2, etc. for each mosquito net in the household (hhnumnet) | | | |
| 99 | HHAMNETS | IMMEDIATELY BEFORE Enrollment, did your household have any mosquito nets that can be used while sleeping? | |
| 100 | HNUMNETS | IMMEDIATELY BEFORE study enrollment, How many mosquito nets did your household have? | |
| 101 | OBS1 | May I have a look at (all) the net(s) to establish the brand? | |
| 102 | MNTHS1 | How many months ago did your household obtain the mosquito net? | |
| 103 | WHERE1 | Where did you get the mosquito net from? | |
| 104 | SPCFRO1 | Specify other sources of the mosquito net | |
| 105 | BRAND1 | OBSERVE OR ASK THE BRAND OR TYPE OF MOSQUITO NET. | |
| 106 | OTHERB1 | Specify other brands or types of mosquito net | |
| 107 | SMNET1 | Since you got the mosquito net, was it ever soaked or dipped in a liquid to repel mosquitoes or bugs? | |
| 108 | TSMNET1 | How many months ago was the net last soaked or dipped? | |
| 109 | SLPNET1 | Did anyone sleep under this mosquito net last night? | |
| 110 | NUSED1A | What are some of the reasons why this net was not used?  ...Too hot | |
| 111 | NUSED1B | ...Don't like smell | |
| 112 | NUSED1C | ...No mosquitoes | |
| 113 | NUSED1D | ...Net too old/too many holes | |
| 114 | NUSED1E | ...Net not hung | |
| 115 | NUSED1F | ...Net too dirty | |
| 116 | NUSED1G | ...Net no longer kill insects | |
| 117 | NUSED1H | ...Don’t know | |
| 118 | NUSED1I | ...Other | |
| 119 | NTHUNG1 | If not hung, why not? | |
| 120 | OTHRNT1 | Specify other reason why the net was not hung. | |
| **Section 5: Interviewer Details** | | | |
| 122 | STOPTIME | End time of interview | |
| 123 | VSTATUS | Result of Visit | |
| 124 | OVSTATUS | Specify other result | |
| 125 | TOTVISIT | Total number of visits | |
| 126 | COMMENTS | Interviewer's Comments | |
|  |  | |  |
|  |  | |  |
|  |  | |  |
|  |  | |  |
|  |  | |  |
|  |  | |  |
|  |  | |  |

**Appendix D. Administration of study drugs and placebos**

Timing of administration of study drugs during pregnancy

| **Weeks of gestation** | **3 dose SP**  **(treatment arm A)** | **3 dose DP**  **(treatment arms B+C)** | **Monthly DP**  **(treatment arms D+E)** |
| --- | --- | --- | --- |
| 16 | DP placebo + SP placebo | DP placebo + SP placebo | DP + SP placebo |
| 20 | SP + DP placebo | DP + SP placebo | DP + SP placebo |
| 24 | DP placebo + SP placebo | DP placebo + SP placebo | DP + SP placebo |
| 28 | SP + DP placebo | DP + SP placebo | DP + SP placebo |
| 32 | DP placebo + SP placebo | DP placebo + SP placebo | DP + SP placebo |
| 36 | SP + DP placebo | DP + SP placebo | DP + SP placebo |
| 40 | DP placebo + SP placebo | DP placebo + SP placebo | DP + SP placebo |

**Timing of administration of study drugs during infancy**

| **Weeks of age** | **3 monthly DP**  **(treatment arms A+B+D)** | **Monthly DP**  **(treatment arms C+E)** |
| --- | --- | --- |
| 8 | DP | DP |
| 12 | DP placebo | DP |
| 16 | DP placebo | DP |
| 20 | DP | DP |
| 24 | DP placebo | DP |
| 28 | DP placebo | DP |
| 32 | DP | DP |
| 36 | DP placebo | DP |
| 40 | DP placebo | DP |
| 44 | DP | DP |
| 48 | DP placebo | DP |
| 52 | DP placebo | DP |
| 56 | DP | DP |
| 60 | DP placebo | DP |
| 64 | DP placebo | DP |
| 68 | DP | DP |
| 72 | DP placebo | DP |
| 76 | DP placebo | DP |
| 80 | DP | DP |
| 84 | DP placebo | DP |
| 88 | DP placebo | DP |
| 92 | DP | DP |
| 96 | DP placebo | DP |
| 100 | DP placebo | DP |
| 104 | DP | DP |

**Appendix E. Weight-based dosing guidelines for DP during infancy**

| **Weight (kg)** | **Dihydroartemisinin-Piperaquine (20mg/160mg tabs)**  **monthly dosing given once a day for 3 consecutive days** | | |
| --- | --- | --- | --- |
|  | **Day 1** | **Day 2** | **Day 3** |
| < 5.9 | ½ tab | ½ tab | ½ tab |
| 6.0-10.9 | 1 tab | 1 tab | 1 tab |
| 11.0-14.9 | 1 ½ tab | 1 ½ tab | 1 ½ tab |
| 15.0-19.9 | 2 tabs | 2 tabs | 2 tabs |
| 20.0-23.9 | 2 ½ tab | 2 ½ tab | 2 ½ tab |
| 24.0-25.9 | 3 tabs | 3 tabs | 3 tabs |

**Appendix F. WHO Criteria for Severe Malaria and Danger Signs**

Criteria for severe malaria

Cerebral malaria - defined as unarousable coma not attributable to any other cause in a patient with falciparum malaria

Generalized convulsions (> 3 convulsions over 24 hours period)

Severe normocytic anemia (Hb < 5 gm/dL)

Hypoglycemia

Metabolic acidosis with respiratory distress

Fluid and electrolyte disturbances

Acute renal failure

Acute pulmonary edema and adult respiratory distress syndrome (ARDS)

Circulatory collapse, shock, septicemia ("algid malaria")

Abnormal bleeding

Jaundice

Danger signs (in children only)

Less than 3 convulsions over 24 hour period

Inability to sit up or stand

Vomiting everything

Unable to breastfeed or drink

Lethargy

**Appendix G. Uganda Ministry of Health Guidelines for Routine Care of Pregnant and Postpartum Women, and Newborns**

In addition to receiving medical care as described above in the protocol, women and infants enrolled in the study will receive standard routine prenatal and postpartum care according to Uganda Ministry of Health guidelines. These standard procedures are subject to availability at Tororo District Hospital.

**Routine antenatal care**. Women enrolled in the study will receive routine care as designated in the Uganda Ministry of Health Guidelines. Routine antenatal care includes screening and treating for syphilis and syndromic management of sexually transmitted infections (STIs). Pregnant women will receive iron and folic acid supplementation. In addition, women will be given multivitamins that will be given once daily. In addition, women receive mebendazole 500mg as a single dose as early as possible after the 1^st^ trimester. Each antenatal visit also includes blood pressure assessment and urine dip stick for proteinuria.

**Routine intrapartum/delivery care.** Routine delivery care for in-hospital births will include labor management by the midwifery staff and management of obstetrical complications as per Ministry of Health guidelines. Immediate postpartum infant care will include polio and BCG immunization, ophthalmic tetracycline, and vitamin K.

**Routine postpartum care.** All postpartum women will receive vitamin A supplementation (200,000 IU) immediately following delivery. Depending on clinical circumstances and based on local standard of care, women may receive 2 weeks of multivitamins twice a day. Common indications for postpartum multivitamins include anemia, postpartum hemorrhage and prolonged labor. Women will be seen at 1 week after delivery as per Ugandan standards of care. Women also undergo a 6 weeks postpartum visit as part of routine care. These visits include an abdominal exam, syndromic management of STIs,and follow-up on any obstetrical complications that occurred. In addition, women receive vitamin A at this visit, if not given immediately postpartum, and are continued on iron and folic acid supplementation. Pelvic and breast exam will be done if clinically indicated. Contraceptive counseling is performed at this visit as is a nutritional assessment and infant feeding and support. Screening for cervical cancer will be performed postpartum by clinical TDH staff if available at Tororo District Hospital.

**Routine infant care.** Infants will be referred to the TDH antenatal clinic for routine immunizations at 6 weeks, 10 weeks, 14 weeks, 6 and 9 months of life.

**Appendix H. Schedule of routine assessments and procedures** in pregnant women

| **Evaluations and Interventions** | **Enrollment** | **Weeks of gestation** | | | | | | | **Delivery** | **1 and 6 weeks postpartum** |
| --- | --- | --- | --- | --- | --- | --- | --- | --- | --- | --- |
|  |  | **16*** | **20** | **24** | **28** | **32** | **36** | **40** |  |  |
| **Informed consent** | X |  |  |  |  |  |  |  |  |  |
| **HIV testing^1^** | X |  |  |  | X |  |  |  | X |  |
| **Pregnancy confirmation^2^** | X |  |  |  |  |  |  |  |  |  |
| **Obstetrical ultrasound^3^** | X |  |  |  |  |  |  |  |  |  |
| **Blood collected by phlebotomy for CBC, ALT, and immunology studies** | X |  | X |  | X |  | X |  | X |  |
| **Blood collected by finger prick for dried blood spot and plasma** | X | X | X | X | X | X | X | X | X |  |
| **Routine assessment in the study clinic^4^** | X | X | X | X | X | X | X | X |  | X |
| **Administration of study drugs** |  | X | X | X | X | X | X | X |  |  |
| **Collection of cord blood and placental tissue** |  |  |  |  |  |  |  |  | X |  |
| **Labor and delivery documentation^5^** |  |  |  |  |  |  |  |  | X |  |
| Standard Care | | | | | | | | | | |
| **Obstetrical exam^6^** | X | X | X | X | X | X | X | X | X | X |
| **Syphilis screening** | X |  |  |  |  |  |  |  |  |  |
| **Iron and Folic Acid** | X | X | X | X | X | X | X | X |  |  |
| **Prenatal vitamins** | X |  |  |  |  |  |  |  |  |  |
| **Mebendazole^7^** |  | X | |  |  |  |  |  |  |  |
| **Screening for non-malarial parasitic infections^8^** |  | X | |  |  |  |  |  |  |  |
| **Vitamin A^9^** |  |  |  |  |  |  |  |  | X |  |
| **Insecticide treated bednet** | X |  |  |  |  |  |  |  | X |  |

***** Only if study subject enrolled prior to 16 weeks gestation; If the woman is enrolled between 18 or 20 weeks, then the week 20 phlebotomy will not be performed.

**Explanation of maternal schedule of events**

1. HIV test will be done at enrollment and documented. A repeat rapid HIV test will be done at delivery. HIV testing shall be done using standard rapid HIV-testing algorithm.
2. Pregnancy confirmation by positive urine pregnancy test, or confirmed intrauterine pregnancy by ultrasound. A pregnancy test may be skipped if an intrauterine pregnancy has been noted on ultrasound at the screening visit.
3. Ultrasound will be done to confirm intrauterine pregnancy and estimate gestational age at enrollment. See Appendix B for dating criteria.
4. Targeted physical exam will include anthropometric measurements (e.g. weight) and vital signs (i.e. temperature, pulse, and blood pressure). Measurement of height at the enrollment visit only.
5. Labor & Delivery documentation will include: Peripartum history, mode of delivery, Apgar scores (when available), weight, length, and head circumference of the child at birth, approximate gestational age, duration of labor, signs of fetal distress (presence of meconium), summary of events in first days of life (including feeding, breathing patterns, jaundice, lethargy, or any additional abnormal findings), duration of admission if delivered in hospital.
6. Obstetrical exam includes estimation of gestational age at study entry, fundal height measurement, fetal heart tones and urine dipstick for protein. A cervical exam will also be performed at screening and during antepartum study visits as clinically indicated.
7. Mebendazole is typically given as 500mg as a single dose as early as possible after the 1^st^ trimester (16 or 20 week visit).
8. Screening for non-malarial parasitic infections will be done prior to administering Mebendazole and will include stool ova and parasite examination, circulating filarial antigens, and blood smear for microfilaremia.
9. Vitamin A supplementation is dosed as 200,000 IU.

**Appendix I. Schedule of routine assessments and procedures in children**

| **Weeks of age** | **Blood collected by finger prick** | | | **Blood collected by phlebotomy for CBC, and immunology studies** | **Blood collected by phlebotomy for ALT** | **Routine assessment in the study clinic** | **Administration of study drugs** | **Screening for non-malarial parasitic infections** |
| --- | --- | --- | --- | --- | --- | --- | --- | --- |
|  | **Blood smear** | **Dried blood spots** | **Plasma** |  |  |  |  |  |
| 4 | X | X | X |  |  | X |  |  |
| 8 | X | X | X | X | X | X | X |  |
| 12 | X | X | X |  |  | X | X |  |
| 16 | X | X | X |  |  | X | X |  |
| 20 | X | X | X |  |  | X | X |  |
| 24 | X | X | X | X | X | X | X |  |
| 28 | X | X | X |  |  | X | X |  |
| 32 | X | X | X |  |  | X | X |  |
| 36 | X | X | X |  |  | X | X |  |
| 40 | X | X | X | X | X | X | X |  |
| 44 | X | X | X |  |  | X | X |  |
| 48 | X | X | X |  |  | X | X |  |
| 52 | X | X | X |  |  | X | X |  |
| 56 | X | X | X | X | X | X | X | X |
| 60 | X | X | X |  |  | X | X |  |
| 64 | X | X | X |  |  | X | X |  |
| 68 | X | X | X |  |  | X | X |  |
| 72 | X | X | X |  | X | X | X |  |
| 76 | X | X | X |  |  | X | X |  |
| 80 | X | X | X |  |  | X | X |  |
| 84 | X | X | X |  |  | X | X |  |
| 88 | X | X | X | X | X | X | X |  |
| 92 | X | X | X |  |  | X | X |  |
| 96 | X | X | X |  |  | X | X |  |
| 100 | X | X | X |  |  | X | X |  |
| 104 | X | X | X | X | X | X | X |  |
| 108 | X | X |  |  |  | X |  |  |
| 112 | X | X |  |  |  | X |  |  |
| 116 | X | X |  |  |  | X |  |  |
| 120 | X | X |  | X |  | X |  |  |
| 124 | X | X |  |  |  | X |  |  |
| 128 | X | X |  |  |  | X |  |  |
| 132 | X | X |  |  |  | X |  |  |
| 136 | X | X |  | X |  | X |  |  |
| 140 | X | X |  |  |  | X |  |  |
| 144 | X | X |  |  |  | X |  |  |
| 148 | X | X |  |  |  | X |  |  |
| 152 | X | X |  |  |  | X |  |  |
| 156 | X | X |  | X |  | X |  |  |
|  |  |  |  |  |  |  |  |  |
|  |  |  |  |  |  |  |  |  |
|  |  |  |  |  |  |  |  |  |
|  |  |  |  |  |  |  |  |  |
|  |  |  |  |  |  |  |  |  |
|  |  |  |  |  |  |  |  |  |
|  |  |  |  |  |  |  |  |  |
|  |  |  |  |  |  |  |  |  |
|  |  |  |  |  |  |  |  |  |
|  |  |  |  |  |  |  |  |  |
|  |  |  |  |  |  |  |  |  |
|  |  |  |  |  |  |  |  |  |
|  |  |  |  |  |  |  |  |  |
|  |  |  |  |  |  |  |  |  |
|  |  |  |  |  |  |  |  |  |
|  |  |  |  |  |  |  |  |  |
|  |  |  |  |  |  |  |  |  |
|  |  |  |  |  |  |  |  |  |
|  |  |  |  |  |  |  |  |  |
|  |  |  |  |  |  |  |  |  |
|  |  |  |  |  |  |  |  |  |
|  |  |  |  |  |  |  |  |  |
|  |  |  |  |  |  |  |  |  |
|  |  |  |  |  |  |  |  |  |
|  |  |  |  |  |  |  |  |  |
|  |  |  |  |  |  |  |  |  |
|  |  |  |  |  |  |  |  |  |
|  |  |  |  |  |  |  |  |  |
|  |  |  |  |  |  |  |  |  |
|  |  |  |  |  |  |  |  |  |
|  |  |  |  |  |  |  |  |  |
|  |  |  |  |  |  |  |  |  |

**Appendix J. DAIDS Toxicity Table (Dec 2004, clarification Aug 2009)**

**Division of AIDS Table for Grading the Severity of ADULT AND PEDIATRIC Adverse Events Version 1.0, December, 2004; clarification AUGUST 2009**

The Division of AIDS Table for Grading the Severity of Adult and Pediatric Adverse Events (“DAIDS AE Grading Table”) is a descriptive terminology which can be utilized for Adverse Event (AE) reporting. A grading (severity) scale is provided for each AE term.

This clarification of the DAIDS Table for Grading the Severity of Adult and Pediatric AE’s provides additional explanation of the DAIDS AE Grading Table and clarifies some of the parameters.

**I. Instructions and Clarifications**

**Grading Adult and Pediatric AEs**

The DAIDS AE Grading Table includes parameters for grading both Adult and Pediatric AEs. When a single set of parameters is not appropriate for grading specific types of AEs for both Adult and Pediatric populations, separate sets of parameters for Adult and/or Pediatric populations (with specified respective age ranges) are given in the Table. If there is no distinction in the Table between Adult and Pediatric values for a type of AE, then the single set of parameters listed is to be used for grading the severity of both Adult and Pediatric events of that type.

**Note:** In the classification of adverse events, the term “**severe**” is not the same as “**serious**.” Severity is an indication of the intensity of a specific event (as in mild, moderate, or severe chest pain). The term “**serious**” relates to a participant/event outcome or action criteria, usually associated with events that pose a threat to a participant’s life or functioning.

Grade 5

For any AE where the outcome is death, the severity of the AE is classified as Grade 5.

Estimating Severity Grade for Parameters Not Identified in the Table

In order to grade a clinical AE that is not identified in the DAIDS AE grading table, use the category “Estimating Severity Grade” located on Page 3.

Determining Severity Grade for Parameters “Between Grades”

If the severity of a clinical AE could fall under either one of two grades (e.g., the severity of an AE could be either Grade 2 or Grade 3), select the higher of the two grades for the AE. If a laboratory value that is graded as a multiple of the ULN or LLN falls between two grades, select the higher of the two grades for the AE. For example, Grade 1 is 2.5 x ULN and Grade 2 is 2.6 x ULN for a parameter. If the lab value is 2.53 x ULN (which is between the two grades), the severity of this AE would be Grade 2, the higher of the two grades.

Values Below Grade 1

Any laboratory value that is between either the LLN or ULN and Grade 1 should not be graded.

Determining Severity Grade when Local Laboratory Normal Values Overlap with Grade 1 Ranges

In these situations, the severity grading is based on the ranges in the DAIDS AE Grading Table, even when there is a reference to the local lab LLN.

*For example: Phosphate, Serum, Low, Adult and Pediatric > 14 years (Page 20) Grade 1 range is 2.50 mg/dL - < LLN.* A particular laboratory’s normal range for Phosphate is 2.1 – 3.8 mg/dL. A participant’s actual lab value is 2.5. In this case, the value of 2.5 exceeds the LLN for the local lab, but will be graded as Grade 1 per DAIDS AE Grading Table.

**II. Definitions of terms used in the Table:**

| Basic Self-care Functions | Adult  Activities such as bathing, dressing, toileting, transfer/movement, continence, and feeding.  Young Children  Activities that are age and culturally appropriate (e.g., feeding self with culturally appropriate eating implement). |
| --- | --- |
| LLN | Lower limit of normal |
| Medical Intervention | Use of pharmacologic or biologic agent(s) for treatment of an AE. |
| NA | Not Applicable |
| Operative Intervention | Surgical OR other invasive mechanical procedures. |
| ULN | Upper limit of normal |
| Usual Social & Functional Activities | Adult  Adaptive tasks and desirable activities, such as going to work, shopping, cooking, use of transportation, pursuing a hobby, etc.  Young Children  Activities that are age and culturally appropriate (e.g., social interactions, play activities, learning tasks, etc.). |

| PARAMETER | | | | | GRADE 1 MILD | | | GRADE 2 MODERATE | | | | GRADE 3 SEVERE | | | GRADE 4 POTENTIALLY LIFE-THREATENING | | |
| --- | --- | --- | --- | --- | --- | --- | --- | --- | --- | --- | --- | --- | --- | --- | --- | --- | --- |
| ESTIMATING SEVERITY GRADE | | | | | | | | | | | | | | | | | |
| Clinical adverse event NOT identified elsewhere in this DAIDS AE Grading Table | | | | | Symptoms causing no or minimal interference with usual social & functional activities | | | Symptoms causing greater than minimal interference with usual social & functional activities | | | | Symptoms causing inability to perform usual social & functional activities | | | Symptoms causing inability to perform basic self-care functions OR Medical or operative intervention indicated to prevent permanent impairment, persistent disability, or death | | |
| SYSTEMIC | | | | | | | | | | | | | | | | | |
| Acute systemic allergic reaction | | | | | Localized urticaria (wheals) with no medical intervention indicated | | | Localized urticaria with medical intervention indicated OR Mild angioedema with no medical intervention indicated | | | | Generalized urticaria OR Angioedema with medical intervention indicated OR Symptomatic mild bronchospasm | | | Acute anaphylaxis OR Life-threatening bronchospasm OR laryngeal edema | | |
| Chills | | | | | Symptoms causing no or minimal interference with usual social & functional activities | | | Symptoms causing greater than minimal interference with usual social & functional activities | | | | Symptoms causing inability to perform usual social & functional activities | | | NA | | |
| Fatigue  Malaise | | | | | Symptoms causing no or minimal interference with usual social & functional activities | | | Symptoms causing greater than minimal interference with usual social & functional activities | | | | Symptoms causing inability to perform usual social & functional activities | | | Incapacitating fatigue/ malaise symptoms causing inability to perform basic self-care functions | | |
| Fever (nonaxillary) | | | | | 37.7 – 38.6°C | | | 38.7 – 39.3°C | | | | 39.4 – 40.5°C | | | > 40.5°C | | |
| Pain (indicate body site)  DO NOT use for pain due to injection (See Injection Site Reactions: Injection site pain)  See also Headache, Arthralgia, and Myalgia | | | | | Pain causing no or minimal interference with usual social & functional activities | | | Pain causing greater than minimal interference with usual social & functional activities | | | | Pain causing inability to perform usual social & functional activities | | | Disabling pain causing inability to perform basic self-care functions OR Hospitalization (other than emergency room visit) indicated | | |
| Unintentional weight loss | | | | | NA | | | 5 – 9% loss in body weight from baseline | | | | 10 – 19% loss in body weight from baseline | | | ≥ 20% loss in body weight from baseline OR Aggressive intervention indicated [e.g., tube feeding or total parenteral nutrition (TPN)] | | |
| INFECTION | | | | | | | | | | | | | | | | | |
| Infection (any other than HIV infection) | | | | | Localized, no systemic antimicrobial treatment indicated AND Symptoms causing no or minimal interference with usual social & functional activities | | | Systemic antimicrobial treatment indicated OR Symptoms causing greater than minimal interference with usual social & functional activities | | | | Systemic antimicrobial treatment indicated AND Symptoms causing inability to perform usual social & functional activities OR Operative intervention (other than simple incision and drainage) indicated | | | Life-threatening consequences (e.g., septic shock) | | |
| **INJECTION SITE REACTIONS** | | | | | | | | | | | | | | | | | |
| Injection site pain (pain without touching)  Or  Tenderness (pain when area is touched) | | | | | Pain/tenderness causing no or minimal limitation of use of limb | | | Pain/tenderness limiting use of limb OR Pain/tenderness causing greater than minimal interference with usual social & functional activities | | | | Pain/tenderness causing inability to perform usual social & functional activities | | | Pain/tenderness causing inability to perform basic self-care function OR Hospitalization (other than emergency room visit) indicated for management of pain/tenderness | | |
| Injection site reaction (localized) | | | | | | | | | | | | | | | | | |
|  | | **Adult > 15 years** | | | | Erythema OR Induration of 5x5 cm – 9x9 cm (or 25 cm^2^ – 81cm^2^) | | | Erythema OR Induration OR Edema  > 9 cm any diameter (or > 81 cm^2^) | Ulceration OR Secondary infection OR Phlebitis OR Sterile abscess OR Drainage | | | | | | | Necrosis (involving dermis and deeper tissue) |
|  | | **Pediatric ≤ 15 years** | | | | Erythema OR Induration OR Edema present but ≤ 2.5 cm diameter | | | Erythema OR Induration OR Edema > 2.5 cm diameter but < 50% surface area of the extremity segment (e.g., upper arm/thigh) | Erythema OR Induration OR Edema involving  ≥ 50% surface area of the extremity segment (e.g., upper arm/thigh) OR Ulceration OR Secondary infection OR Phlebitis OR Sterile abscess OR Drainage | | | | | | | Necrosis (involving dermis and deeper tissue) |
| Pruritis associated with injection  See also Skin: Pruritis (itching - no skin lesions) | | | | | Itching localized to injection site AND Relieved spontaneously or with < 48 hours treatment | | | Itching beyond the injection site but not generalized OR Itching localized to injection site requiring ≥ 48 hours treatment | | | | Generalized itching causing inability to perform usual social & functional activities | | | NA | | |
| SKIN – DERMATOLOGICAL | | | | | | | | | | | | | | | | | |
| Alopecia | | | | | Thinning detectable by study participant (or by caregiver for young children and disabled adults) | | | Thinning or patchy hair loss detectable by health care provider | | | | Complete hair loss | | | NA | | |
| Cutaneous reaction – rash | | | | | Localized macular rash | | | Diffuse macular, maculopapular, or morbilliform rash OR Target lesions | | | | Diffuse macular, maculopapular, or morbilliform rash with vesicles or limited number of bullae OR Superficial ulcerations of mucous membrane limited to one site | | | Extensive or generalized bullous lesions OR Stevens-Johnson syndrome OR Ulceration of mucous membrane involving two or more distinct mucosal sites OR Toxic epidermal necrolysis (TEN) | | |
| Hyperpigmentation | | | | | Slight or localized | | | Marked or generalized | | | | NA | | | NA | | |
| Hypopigmentation | | | | | Slight or localized | | | Marked or generalized | | | | NA | | | NA | | |
| Pruritis (itching – no skin lesions)  (See also Injection Site Reactions: Pruritis associated with injection) | | | | | Itching causing no or minimal interference with usual social & functional activities | | | Itching causing greater than minimal interference with usual social & functional activities | | | | Itching causing inability to perform usual social & functional activities | | | NA | | |
| CARDIOVASCULAR | | | | | | | | | | | | | | | | | |
| Cardiac arrhythmia (general)  (By ECG or physical exam) | | | | | | Asymptomatic AND No intervention indicated | | | Asymptomatic AND Non-urgent medical intervention indicated | Symptomatic, non-life-threatening AND Non-urgent medical intervention indicated | | | | | | | Life-threatening arrhythmia OR Urgent intervention indicated |
| Cardiac-ischemia/infarction | | | | | NA | | | NA | | | | Symptomatic ischemia (stable angina) OR Testing consistent with ischemia | | | Unstable angina OR Acute myocardial infarction | | |
| Hemorrhage (significant acute blood loss) | | | | | NA | | | Symptomatic AND No transfusion indicated | | | | Symptomatic AND Transfusion of ≤ 2 units packed RBCs (for children ≤ 10 cc/kg) indicated | | | Life-threatening hypotension OR Transfusion of > 2 units packed RBCs (for children > 10 cc/kg) indicated | | |
| Hypertension | | | | | | | | | | | | | | | | | |
|  | | | **Adult > 17 years**  (with repeat testing at same visit) | | | 140 – 159 mmHg systolic  OR  90 – 99 mmHg diastolic | | | 160 – 179 mmHg systolic  OR  100 – 109 mmHg diastolic | | | | **≥** 180 mmHg systolic  OR  **≥** 110 mmHg diastolic | | | | Life-threatening consequences (e.g., malignant hypertension) OR Hospitalization indicated (other than emergency room visit) |
|  | | | **Correction**: in Grade 2 to 160 - 179 from > 160-179 (systolic) and to ≥ 100 -109 from > 100-109 (diastolic) and  in Grade 3 to  **≥** 180 from > 180 (systolic) and to  **≥** 110 from > 110 (diastolic). | | | | | | | | | | | | | | |
|  | | | **Pediatric ≤ 17 years**  (with repeat testing at same visit) | | | NA | | | 91^st^ – 94^th^ percentile adjusted for age, height, and gender (systolic and/or diastolic) | | | | ≥ 95^th^ percentile adjusted for age, height, and gender (systolic and/or diastolic) | | | | Life-threatening consequences (e.g., malignant hypertension) OR Hospitalization indicated (other than emergency room visit) |
| Hypotension | | | | | NA | | | Symptomatic, corrected with oral fluid replacement | | | | Symptomatic, IV fluids indicated | | | Shock requiring use of vasopressors or mechanical assistance to maintain blood pressure | | |
| Pericardial effusion | | | | | Asymptomatic, small effusion requiring no intervention | | | Asymptomatic, moderate or larger effusion requiring no intervention | | | | Effusion with non-life threatening physiologic consequences OR Effusion with non-urgent intervention indicated | | | Life-threatening consequences (e.g., tamponade) OR Urgent intervention indicated | | |
| Thrombosis/embolism | | | | | NA | | | Deep vein thrombosis AND No intervention indicated (e.g., anticoagulation, lysis filter, invasive procedure) | | | | Deep vein thrombosis AND Intervention indicated (e.g., anticoagulation, lysis filter, invasive procedure) | | | Embolic event (e.g., pulmonary embolism, life-threatening thrombus) | | |
| Vasovagal episode (associated with a procedure of any kind) | | | | | Present without loss of consciousness | | | Present with transient loss of consciousness | | | | NA | | | NA | | |
| Ventricular dysfunction (congestive heart failure) | | | | | NA | | | Asymptomatic diagnostic finding AND intervention indicated | | | | New onset with symptoms OR Worsening symptomatic congestive heart failure | | | Life-threatening congestive heart failure | | |
| GASTROINTESTINAL | | | | | | | | | | | | | | | | | |
| Anorexia | | | | | Loss of appetite without decreased oral intake | | | Loss of appetite associated with decreased oral intake without significant weight loss | | | | Loss of appetite associated with significant weight loss | | | Life-threatening consequences OR Aggressive intervention indicated [e.g., tube feeding or total parenteral nutrition (TPN)] | | |
| **Comment:** Please note that, while the grading scale provided for Unintentional Weight Loss may be used as a guideline when grading anorexia, this is not a requirement and should not be used as a substitute for clinical judgment. | | | | | | | | | | | | | | | | | |
| Ascites | | | | | Asymptomatic | | | Symptomatic AND Intervention indicated (e.g., diuretics or therapeutic paracentesis) | | | | Symptomatic despite intervention | | | Life-threatening consequences | | |
| Cholecystitis | | | | | NA | | | Symptomatic AND Medical intervention indicated | | | | Radiologic, endoscopic, or operative intervention indicated | | | Life-threatening consequences (e.g., sepsis or perforation) | | |
| Constipation | | | | | NA | | | Persistent constipation requiring regular use of dietary modifications, laxatives, or enemas | | | | Obstipation with manual evacuation indicated | | | Life-threatening consequences (e.g., obstruction) | | |
| Diarrhea | | | | | | | | | | | | | | | | | |
|  | | **Adult and Pediatric ≥ 1 year** | | | | Transient or intermittent episodes of unformed stools OR Increase of ≤ 3 stools over baseline per 24-hour period | | | Persistent episodes of unformed to watery stools OR Increase of 4 – 6 stools over baseline per 24-hour period | Bloody diarrhea OR Increase of ≥ 7 stools per 24-hour period OR IV fluid replacement indicated | | | | | | | Life-threatening consequences (e.g., hypotensive shock) |
|  | | **Pediatric < 1 year** | | | | Liquid stools (more unformed than usual) but usual number of stools | | | Liquid stools with increased number of stools OR Mild dehydration | Liquid stools with moderate dehydration | | | | | | | Liquid stools resulting in severe dehydration with aggressive rehydration indicated OR Hypotensive shock |
| Dysphagia-Odynophagia | | | | | Symptomatic but able to eat usual diet | | | Symptoms causing altered dietary intake without medical intervention indicated | | | | Symptoms causing severely altered dietary intake with medical intervention indicated | | | Life-threatening reduction in oral intake | | |
| Mucositis/stomatitis  (clinical exam)  Indicate site (e.g., larynx, oral)  See Genitourinary for Vulvovaginitis  See also Dysphagia-Odynophagia and Proctitis | | | | | Erythema of the mucosa | | | Patchy pseudomembranes or ulcerations | | | | Confluent pseudomembranes or ulcerations OR Mucosal bleeding with minor trauma | | | Tissue necrosis OR Diffuse spontaneous mucosal bleeding OR Life-threatening consequences (e.g., aspiration, choking) | | |
| Nausea | | | | | Transient (< 24 hours) or intermittent nausea with no or minimal interference with oral intake | | | Persistent nausea resulting in decreased oral intake for 24 – 48 hours | | | | Persistent nausea resulting in minimal oral intake for > 48 hours OR Aggressive rehydration indicated (e.g., IV fluids) | | | Life-threatening consequences (e.g., hypotensive shock) | | |
| Pancreatitis | | | | | NA | | | Symptomatic AND Hospitalization not indicated (other than emergency room visit) | | | | Symptomatic AND Hospitalization indicated (other than emergency room visit) | | | Life-threatening consequences (e.g., circulatory failure, hemorrhage, sepsis) | | |
| Proctitis (functional- symptomatic)  Also see Mucositis/stomatitis  for clinical exam | | | | | Rectal discomfort AND No intervention indicated | | | Symptoms causing greater than minimal interference with usual social & functional activities OR Medical intervention indicated | | | | Symptoms causing inability to perform usual social & functional activities OR Operative intervention indicated | | | Life-threatening consequences (e.g., perforation) | | |
| Vomiting | | | | | Transient or intermittent vomiting with no or minimal interference with oral intake | | | Frequent episodes of vomiting with no or mild dehydration | | | | Persistent vomiting resulting in orthostatic hypotension OR Aggressive rehydration indicated (e.g., IV fluids) | | | Life-threatening consequences (e.g., hypotensive shock) | | |
| NEUROLOGIC | | | | | | | | | | | | | | | | | |
| Alteration in personality-behavior or in mood (e.g., agitation, anxiety, depression, mania, psychosis) | | | | | Alteration causing no or minimal interference with usual social & functional activities | | | Alteration causing greater than minimal interference with usual social & functional activities | | | | Alteration causing inability to perform usual social & functional activities | | | Behavior potentially harmful to self or others (e.g., suicidal and homicidal ideation or attempt, acute psychosis) OR Causing inability to perform basic self-care functions | | |
| Altered Mental Status  For Dementia, see Cognitive and behavioral/attentional disturbance (including dementia and attention deficit disorder) | | | | | Changes causing no or minimal interference with usual social & functional activities | | | Mild lethargy or somnolence causing greater than minimal interference with usual social & functional activities | | | | Confusion, memory impairment, lethargy, or somnolence causing inability to perform usual social & functional activities | | | Delirium OR obtundation, OR coma | | |
| Ataxia | | | | | Asymptomatic ataxia detectable on exam OR Minimal ataxia causing no or minimal interference with usual social & functional activities | | | Symptomatic ataxia causing greater than minimal interference with usual social & functional activities | | | | Symptomatic ataxia causing inability to perform usual social & functional activities | | | Disabling ataxia causing inability to perform basic self-care functions | | |
| Cognitive and behavioral/attentional disturbance (including dementia and attention deficit disorder) | | | | | Disability causing no or minimal interference with usual social & functional activities OR Specialized resources not indicated | | | Disability causing greater than minimal interference with usual social & functional activities OR Specialized resources on part-time basis indicated | | | | Disability causing inability to perform usual social & functional activities OR Specialized resources on a full-time basis indicated | | | Disability causing inability to perform basic self-care functions OR Institutionalization indicated | | |
| CNS ischemia (acute) | | | | | NA | | | NA | | | | Transient ischemic attack | | | Cerebral vascular accident (CVA, stroke) with neurological deficit | | |
| Developmental delay – **Pediatric ≤ 16 years** | | | | | Mild developmental delay, either motor or cognitive, as determined by comparison with a developmental screening tool appropriate for the setting | | | Moderate developmental delay, either motor or cognitive, as determined by comparison with a developmental screening tool appropriate for the setting | | | | Severe developmental delay, either motor or cognitive, as determined by comparison with a developmental screening tool appropriate for the setting | | | Developmental regression, either motor or cognitive, as determined by comparison with a developmental screening tool appropriate for the setting | | |
| Headache | | | | | Symptoms causing no or minimal interference with usual social & functional activities | | | Symptoms causing greater than minimal interference with usual social & functional activities | | | | Symptoms causing inability to perform usual social & functional activities | | | Symptoms causing inability to perform basic self-care functions OR Hospitalization indicated (other than emergency room visit) OR Headache with significant impairment of alertness or other neurologic function | | |
| Insomnia | | | | | NA | | | Difficulty sleeping causing greater than minimal interference with usual social & functional activities | | | | Difficulty sleeping causing inability to perform usual social & functional activities | | | Disabling insomnia causing inability to perform basic self-care functions | | |
| Neuromuscular weakness  (including myopathy & neuropathy) | | | | | Asymptomatic with decreased strength on exam OR Minimal muscle weakness causing no or minimal interference with usual social & functional activities | | | Muscle weakness causing greater than minimal interference with usual social & functional activities | | | | Muscle weakness causing inability to perform usual social & functional activities | | | Disabling muscle weakness causing inability to perform basic self-care functions OR Respiratory muscle weakness impairing ventilation | | |
| Neurosensory alteration (including paresthesia and painful neuropathy) | | | | | Asymptomatic with sensory alteration on exam or minimal paresthesia causing no or minimal interference with usual social & functional activities | | | Sensory alteration or paresthesia causing greater than minimal interference with usual social & functional activities | | | | Sensory alteration or paresthesia causing inability to perform usual social & functional activities | | | Disabling sensory alteration or paresthesia causing inability to perform basic self-care functions | | |
| Seizure: (new onset)  **– Adult ≥ 18 years**  See also Seizure: (known pre-existing seizure disorder) | | | | | NA | | | 1 seizure | | | | 2 **–** 4 seizures | | | Seizures of any kind which are prolonged, repetitive (e.g., status epilepticus), or difficult to control (e.g., refractory epilepsy) | | |
| Seizure: (known pre-existing seizure disorder)  **– Adult ≥ 18 years**  For worsening of existing epilepsy the grades should be based on an increase from previous level of control to any of these levels. | | | | | NA | | | Increased frequency of pre-existing seizures (non-repetitive) without change in seizure character OR Infrequent break-through seizures while on stable medication in a previously controlled seizure disorder | | | | Change in seizure character from baseline either in duration or quality (e.g., severity or focality) | | | Seizures of any kind which are prolonged, repetitive (e.g., status epilepticus), or difficult to control (e.g., refractory epilepsy) | | |
| Seizure  **– Pediatric < 18 years** | | | | | Seizure, generalized onset with or without secondary generalization, lasting < 5 minutes with < 24 hours post ictal state | | | Seizure, generalized onset with or without secondary generalization, lasting 5 – 20 minutes with  < 24 hours post ictal state | | | | Seizure, generalized onset with or without secondary generalization, lasting  > 20 minutes | | | Seizure, generalized onset with or without secondary generalization, requiring intubation and sedation | | |
| Syncope (not associated with a procedure) | | | | | NA | | | Present | | | | NA | | | NA | | |
| Vertigo | | | | | Vertigo causing no or minimal interference with usual social & functional activities | | | Vertigo causing greater than minimal interference with usual social & functional activities | | | | Vertigo causing inability to perform usual social & functional activities | | | Disabling vertigo causing inability to perform basic self-care functions | | |
| RESPIRATORY | | | | | | | | | | | | | | | | | |
| Bronchospasm (acute) | | | | | FEV1 or peak flow reduced to  70 **–** 80% | | | FEV1 or peak flow  50 **–** 69% | | | | FEV1 or peak flow  25 **–** 49% | | | Cyanosis OR FEV1 or peak flow < 25% OR Intubation | | |
| Dyspnea or respiratory distress | | | | | | | | | | | | | | | | | |
|  | | **Adult ≥ 14 years** | | | | Dyspnea on exertion with no or minimal interference with usual social & functional activities | | | Dyspnea on exertion causing greater than minimal interference with usual social & functional activities | | | | Dyspnea at rest causing inability to perform usual social & functional activities | | | Respiratory failure with ventilatory support indicated | |
|  | | **Pediatric < 14 years** | | | | Wheezing OR minimal increase in respiratory rate for age | | | Nasal flaring OR Intercostal retractions OR Pulse oximetry 90 – 95% | | | | Dyspnea at rest causing inability to perform usual social & functional activities OR Pulse oximetry < 90% | | | Respiratory failure with ventilatory support indicated | |
| MUSCULOSKELETAL | | | | | | | | | | | | | | | | | |
| Arthralgia  See also Arthritis | | | | | Joint pain causing no or minimal interference with usual social & functional activities | | | Joint pain causing greater than minimal interference with usual social & functional activities | | | | Joint pain causing inability to perform usual social & functional activities | | | Disabling joint pain causing inability to perform basic self-care functions | | |
| Arthritis  See also Arthralgia | | | | | Stiffness or joint swelling causing no or minimal interference with usual social & functional activities | | | Stiffness or joint swelling causing greater than minimal interference with usual social & functional activities | | | | Stiffness or joint swelling causing inability to perform usual social & functional activities | | | Disabling joint stiffness or swelling causing inability to perform basic self-care functions | | |
| Bone Mineral Loss | | | | | | | | | | | | | | | | | |
|  | **Adult ≥ 21 years** | | | BMD t-score  -2.5 to -1.0 | | | BMD t-score < -2.5 | | | | Pathological fracture (including loss of vertebral height) | | | Pathologic fracture causing life-threatening consequences | | | |
|  | **Pediatric < 21 years** | | | BMD z-score  -2.5 to -1.0 | | | BMD z-score < -2.5 | | | | Pathological fracture (including loss of vertebral height) | | | Pathologic fracture causing life-threatening consequences | | | |
| Myalgia (non-injection site) | | | | | Muscle pain causing no or minimal interference with usual social & functional activities | | | Muscle pain causing greater than minimal interference with usual social & functional activities | | | | Muscle pain causing inability to perform usual social & functional activities | | | Disabling muscle pain causing inability to perform basic self-care functions | | |
| Osteonecrosis | | | | | NA | | | Asymptomatic with radiographic findings AND No operative intervention indicated | | | | Symptomatic bone pain with radiographic findings OR Operative intervention indicated | | | Disabling bone pain with radiographic findings causing inability to perform basic self-care functions | | |
| GENITOURINARY | | | | | | | | | | | | | | | | | |
| Cervicitis  (symptoms)  (For use in studies evaluating topical study agents)  For other cervicitis see Infection: Infection (any other than HIV infection) | | | | | Symptoms causing no or minimal interference with usual social & functional activities | | | Symptoms causing greater than minimal interference with usual social & functional activities | | | | Symptoms causing inability to perform usual social & functional activities | | | Symptoms causing inability to perform basic self-care functions | | |
| Cervicitis  (clinical exam)  (For use in studies evaluating topical study agents)  For other cervicitis see Infection: Infection (any other than HIV infection) | | | | | Minimal cervical abnormalities on examination (erythema, mucopurulent discharge, or friability) OR Epithelial disruption  < 25% of total surface | | | Moderate cervical abnormalities on examination (erythema, mucopurulent discharge, or friability) OR Epithelial disruption of 25 – 49% total surface | | | | Severe cervical abnormalities on examination (erythema, mucopurulent discharge, or friability) OR Epithelial disruption  50 – 75% total surface | | | Epithelial disruption  > 75% total surface | | |
| Inter-menstrual bleeding (IMB) | | | | | Spotting observed by participant OR Minimal blood observed during clinical or colposcopic examination | | | Inter-menstrual bleeding not greater in duration or amount than usual menstrual cycle | | | | Inter-menstrual bleeding greater in duration or amount than usual menstrual cycle | | | Hemorrhage with life-threatening hypotension OR Operative intervention indicated | | |
| Urinary tract obstruction (e.g., stone) | | | | | NA | | | Signs or symptoms of urinary tract obstruction without hydronephrosis or renal dysfunction | | | | Signs or symptoms of urinary tract obstruction with hydronephrosis or renal dysfunction | | | Obstruction causing life-threatening consequences | | |
| Vulvovaginitis  (symptoms)  (Use in studies evaluating topical study agents)  For other vulvovaginitis see Infection: Infection (any other than HIV infection) | | | | | Symptoms causing no or minimal interference with usual social & functional activities | | | Symptoms causing greater than minimal interference with usual social & functional activities | | | | Symptoms causing inability to perform usual social & functional activities | | | Symptoms causing inability to perform basic self-care functions | | |
| Vulvovaginitis  (clinical exam)  (Use in studies evaluating topical study agents)  For other vulvovaginitis see Infection: Infection (any other than HIV infection) | | | | | Minimal vaginal abnormalities on examination OR Epithelial disruption  < 25% of total surface | | | Moderate vaginal abnormalities on examination OR Epithelial disruption of 25 - 49% total surface | | | | Severe vaginal abnormalities on examination OR Epithelial disruption  50 - 75% total surface | | | Vaginal perforation OR Epithelial disruption > 75% total surface | | |
| OCULAR/VISUAL | | | | | | | | | | | | | | | | | |
| Uveitis | | | | | Asymptomatic but detectable on exam | | | Symptomatic anterior uveitis OR Medical intervention indicated | | | | Posterior or pan-uveitis OR Operative intervention indicated | | | Disabling visual loss in affected eye(s) | | |
| Visual changes (from baseline) | | | | | Visual changes causing no or minimal interference with usual social & functional activities | | | Visual changes causing greater than minimal interference with usual social & functional activities | | | | Visual changes causing inability to perform usual social & functional activities | | | Disabling visual loss in affected eye(s) | | |
| ENDOCRINE/METABOLIC | | | | | | | | | | | | | | | | | |
| Abnormal fat accumulation (e.g., back of neck, breasts, abdomen) | | | | | Detectable by study participant (or by caregiver for young children and disabled adults) | | | Detectable on physical exam by health care provider | | | | Disfiguring OR Obvious changes on casual visual inspection | | | NA | | |
| Diabetes mellitus | | | | | NA | | | New onset without need to initiate medication OR Modification of current medications to regain glucose control | | | | New onset with initiation of medication indicated OR Diabetes uncontrolled despite treatment modification | | | Life-threatening consequences (e.g., ketoacidosis, hyperosmolar non-ketotic coma) | | |
| Gynecomastia | | | | | Detectable by study participant or caregiver (for young children and disabled adults) | | | Detectable on physical exam by health care provider | | | | Disfiguring OR Obvious on casual visual inspection | | | NA | | |
| Hyperthyroidism | | | | | Asymptomatic | | | Symptomatic causing greater than minimal interference with usual social & functional activities OR Thyroid suppression therapy indicated | | | | Symptoms causing inability to perform usual social & functional activities OR Uncontrolled despite treatment modification | | | Life-threatening consequences (e.g., thyroid storm) | | |
| Hypothyroidism | | | | | Asymptomatic | | | Symptomatic causing greater than minimal interference with usual social & functional activities OR Thyroid replacement therapy indicated | | | | Symptoms causing inability to perform usual social & functional activities OR Uncontrolled despite treatment modification | | | Life-threatening consequences (e.g., myxedema coma) | | |
| Lipoatrophy (e.g., fat loss from the face, extremities, buttocks) | | | | | Detectable by study participant (or by caregiver for young children and disabled adults) | | | Detectable on physical exam by health care provider | | | | Disfiguring OR Obvious on casual visual inspection | | | NA | | |

## Adult Laboratory Toxicity Tables

| **Routine Tests** | | | | | | | | | | |
| --- | --- | --- | --- | --- | --- | --- | --- | --- | --- | --- |
| **PARAMETER** | | **Grade 1**  **MILD** | | **Grade 2**  **MODERATE** | | | **Grade 3**  **SEVERE** | | | **Grade 4**  **LIFE THREATENING** |
| **HEMATOLOGY** | | | | | | | | | | |
| WBC, decreased | | 2,000 – 2,500/mm^3^ | | | 1,500 – 1,999/mm^3^ | | | 1,000 – 1,499/mm^3^ | | < 1,000/mm^3^ |
| Absolute neutrophil count (ANC) | | 1,000 – 1,300/mm^3^ | | | 750 – 999/mm^3^ | | | 500 – 749/mm^3^ | | < 500/mm^3^ |
| Hemoglobin (Hb) | | 10.0 – 10.9 g/dL | | | 9.0 – 9.9 g/dL | | | 7.0 – 8.9 g/dL | | < 7.0 g/dL |
| Platelets, decreased | | 100,000 –  124,999/mm^3^ | | | 50,000 –  99,999/mm^3^ | | | 25,000 –  49,999/mm^3^ | | < 25,000/mm^3^ |
| AST (SGOT) | | 1.25 – 2.5 x ULN  56 – 113 U/L | | | 2.6 – 5.0 x ULN  114 – 225 U/L | | | 5.1 – 10.0 x ULN  226 – 450 U/L | | - 1. 10.0 x ULN   > 450 U/L |
| **Non-routine tests available** | | | | | | | | | | |
| **PARAMETER** | **Grade 1**  **MILD** | | **Grade 2**  **MODERATE** | | | **Grade 3**  **SEVERE** | | | | **Grade 4**  **LIFE THREATENING** |
| ALT (SGPT) | 1.25 – 2.5 x ULN  56 – 113 U/L | | 2.6 – 5.0 x ULN  114 – 225 U/L | | | 5.1 – 10.0 x ULN  226 – 450 U/L | | | | > 10.0 x ULN  > 450 U/L |
| Bilirubin (Total) | 1.1 – 1.5 x ULN | | 1.6 – 2.5 x ULN | | | 2.6 – 5.0 x ULN | | | | > 5.0 x ULN |
| Creatinine | 1.1 – 1.3 x ULN | | 1.4 – 1.8 x ULN | | | 1.9 – 3.4 x ULN | | | | ≥ 3.5 x ULN |
| Hyperglycemia | 116 – 160 mg/dL *6.44* – *8.88 mmol/L* | | 161 – 250 mg/dL *8.89 – 13.88 mmol/L* | | | 251 – 500 mg/dL *13.89 – 27.75 mmol/L* | | | | > 500 mg/dL *> 27.75 mmol/L* |
| Hypoglycemia | 55 – 64 mg/dL *3.05 – 3.55 mmol/L* | | 40 – 54 mg/dL *2.22 – 3.06 mmol/L* | | | 30 – 39 mg/dL *1.67* – *2.23 mmol/L* | | | | < 30 mg/dL *< 1.67 mmol/L* |
| Potassium, serum, high | 5.6 – 6.0 mEq/L *5.6* – *6.0 mmol/L* | | 6.1 – 6.5 mEq/L *6.1* – *6.5 mmol/L* | | | 6.6 – 7.0 mEq/L *6.6 – 7.0 mmol/L* | | | | > 7.0 mEq/L *> 7.0 mmol/L* |
| Potassium, serum, low | 3.0 – 3.4 mEq/L *3.0 – 3.4 mmol/L* | | 2.5 – 2.9 mEq/L *2.5* – *2.9 mmol/L* | | | 2.0 – 2.4 mEq/L *2.0* – *2.4 mmol/L* | | | | < 2.0 mEq/L *< 2.0 mmol/L* |
| Sodium, serum, high | 146 – 150 mEq/L *146* – *150 mmol/L* | | 151 – 154 mEq/L *151* – *154 mmol/L* | | | 155 – 159 mEq/L *155* – *159 mmol/L* | | | | ≥ 160 mEq/L *≥ 160 mmol/L* |
| Sodium, serum, low | 130 – 135 mEq/L *130 – 135 mmol/L* | | 125 – 129 mEq/L *125* – *129 mmol/L* | | | 121 – 124 mEq/L *121* – *124 mmol/L* | | | | ≤ 120 mEq/L *≤ 120 mmol/L* |
| **Urinalysis** | | | | | | | | | | |
| Hematuria (microscopic) | 6 – 10 RBC/HPF | | > 10 RBC/HPF | | | Gross, with or without clots OR with RBC casts | | | Transfusion indicated | |
| Proteinuria, random  collection | 1 + (30 mg/dL) | | 2 – 3 + (100-300 mg/dL) | | | 4 + (2000 mg/dL) | | | NA | |

## Pediatric Laboratory Toxicity Tables

| **Routine Tests** | | | | | | | | | | |
| --- | --- | --- | --- | --- | --- | --- | --- | --- | --- | --- |
| **PARAMETER** | | **Grade 1**  **MILD** | | **Grade 2**  **MODERATE** | | | **Grade 3**  **SEVERE** | | | **Grade 4**  **LIFE THREATENING** |
| **HEMATOLOGY** | | | | | | | | | | |
| WBC, decreased | | 2,000 – 2,500/mm3 | | | 1,500 – 1,999/mm3 | | | 1,000 – 1,499/mm3 | | < 1,000/mm3 |
| Absolute neutrophil count (ANC) | | 750-1200/mm3 | | | 400-749/mm3 | | | 250-399/mm3 | | < 250/mm3 |
| Hemoglobin (Hb) | | 8.5 – 10.0 g/dL | | | 7.5 – 8.4 g/dL | | | 6.50 – 7.4 g/dL | | < 6.5 g/dL |
| Platelets, decreased | | 100,000 –  124,999/mm^3^ | | | 50,000 –  99,999/mm^3^ | | | 25,000 –  49,999/mm^3^ | | < 25,000/mm^3^ |
| AST (SGOT) | | 1.1-<2.0 x ULN | | | 2.0-<3.0 x ULN | | | 3.0-8.0 x ULN | | > 8.0 x ULN |
| **Non-routine tests available** | | | | | | | | | | |
| **PARAMETER** | **Grade 1**  **MILD** | | **Grade 2**  **MODERATE** | | | **Grade 3**  **SEVERE** | | | | **Grade 4**  **LIFE THREATENING** |
| ALT (SGPT) | 1.25 – 2.5 x ULN  56 – 113 U/L | | 2.6 – 5.0 x ULN  114 – 225 U/L | | | 5.1 – 10.0 x ULN  226 – 450 U/L | | | | > 10.0 x ULN  > 450 U/L |
| Bilirubin (Total) | 1.1-<1.5 x ULN | | 1.5-<2.0 x ULN | | | 2.0-3.0 x ULN | | | | > 3.0 x ULN |
| Creatinine 3 months-2 years | 0.6-0.8 x ULN | | 0.9-1.1 x ULN | | | 1.2-1.5 x ULN | | | | > 1.5 x ULN |
| Creatinine 2-12 years | 0.7-1.0 x ULN | | 1.1-1.6 x ULN | | | 1.7-2.0 x ULN | | | | > 2.0 x ULN |
| Hyperglycemia | 116 – 159 mg/dL | | 160 – 249 mg/dL | | | 250 – 500 mg/dL | | | | > 400 mg/dL |
| Hypoglycemia | 55-65 mg/dL | | 40-54 mg/dL | | | 30-39 mg/dL | | | | <30 mg/dL or abnormal glucose and mental status changes |
| Potassium, serum, high | 5.0 – 5.9 mEq/L | | 6.0 – 6.4 mEq/L | | | 6.5 – 7.0 mEq/L | | | | > 7.0 mEq/L |
| Potassium, serum, low | 3.0 – 3.5 mEq/L | | 2.5 – 2.9 mEq/L | | | 2.0 – 2.4 mEq/L | | | | < 2.0 mEq/L |
| Sodium, serum, high | - | | 145 – 149 mEq/L | | | 150 – 155 mEq/L | | | | > 155 mEq/L |
| Sodium, serum, low | - | | 130 – 135 mEq/L | | | 124 – 129 mEq/L | | | | < 124 mEq/L |
| **Urinalysis** | | | | | | | | | | |
| Hematuria (microscopic) | < 25 RBC/HPF | | > 25 RBC/HPF | | | - | | | Gross Hematuria | |
| Proteinuria, random  collection | 1 + | | 2 + | | | 3 + | | | 4 + | |

## Appendix K. List of drugs associated with known risk of torsades de pointes

Substantial evidence supports the conclusion that these drugs prolong the QT interval AND are clearly associated with a risk of TdP, even when taken as directed in official labeling.

Reference: <https://www.crediblemeds.org/>

## Appendix L. List of drugs that potentially inhibit the metabolism of piperaquine

Chloramphenicol

Clarithromycin

Diltiazem

Erythromycin

Fluconazole

Itraconazole

Ketoconazole

Verapamil
