# Supplementary material for: Dihydroartemisinin-piperaquine for intermittent preventive treatment of malaria during pregnancy and risk of malaria in early childhood: A randomized controlled trial
Source: PLoS Med. 2018 Jul 17;15(7):e1002606. doi: 10.1371/journal.pmed.1002606 (PMC6049882; doi:10.1371/journal.pmed.1002606)
Supplement: S1 Table — (DOCX) [file pmed.1002606.s003.docx]

**Supplemental Table. Measures of safety and adverse events.**

| **Outcome** | **Maternal IPTp Treatment arm** | | |
| --- | --- | --- | --- |
|  | **SP8w (n=100)** | **DP8w (n=44)** | **DP4w (n=47)** |
| **Incidence measures** | **Events ^a^** | **Events ^a^** | **Events ^a^** |
| Individual adverse events of any severity ^b^ |  |  |  |
| Cough | 1232 (6.99) | 519 (6.45) | 479 (5.87) |
| Diarrhea | 523 (2.97) | 202 (2.51) | 213 (2.61) |
| Vomiting | 146 (0.83) | 46 (0.57) | 62 (0.76) |
| Rash | 49 (0.28) | 15 (0.19) | 20 (0.25) |
| Conjunctivitis | 37 (0.21) | 14 (0.17) | 11 (0.13) |
| Anorexia | 27 (0.15) | 11 (0.14) | 13 (0.16) |
| Malaise | 18 (0.10) | 3 (0.04) | 7 (0.09) |
| Individual grade 3-4 adverse events |  |  |  |
| Anemia | 1 (0.01) | 1 (0.01) | 1 (0.01) |
| Thrombocytopenia | 1 (0.01) | 2 (0.02) | 0 (0) |
| Respiratory distress | 2 (0.01) | 1 (0.01) | 0 (0) |
| Elevated ALT | 1 (0.01) | 0 (0) | 0 (0) |
| Neutropenia | 2 (0.01) | 0 (0) | 0 (0) |
| Diarrhea | 1 (0.01) | 0 (0) | 1 (0.01) |
| Seizures | 1 (0.01) | 0 (0) | 0 (0) |
| Intestinal obstruction | 2 (0.01) | 0 (0) | 0 (0) |
| Vomiting | 1 (0.01) | 0 (0) | 0 (0) |
| Altered mental status | 0 (0) | 0 (0) | 1 (0.01) |
| Dehydration | 0 (0) | 0 (0) | 1 (0.01) |
| Burns | 1 (0.01) | 0 (0) | 0 (0) |
| All grade 3-4 adverse events | 13 (0.07) | 4 (0.05) | 4 (0.05) |
| Grade 3-4 adverse events possibly related to study drugs | 0 (0) | 0 (0) | 0 (0) |
| All serious adverse events | 8 (0.05) | 3 (0.04) | 4 (0.05) |

**^a^** Number of events (incidence per person year at risk)

**^b^** Includes only those categories with at least ten total events
