# Supplementary material for: Dihydroartemisinin-piperaquine for intermittent preventive treatment of malaria during pregnancy and risk of malaria in early childhood: A randomized controlled trial
Source: PLoS Med. 2018 Jul 17;15(7):e1002606. doi: 10.1371/journal.pmed.1002606 (PMC6049882; doi:10.1371/journal.pmed.1002606)
Supplement: S1 Statistical Analysis Plan — (DOCX) [file pmed.1002606.s004.docx]

Statistical Analysis Plan – Children

| TRIAL FULL TITLE | **Reducing the Burden of Malaria in HIV-uninfected Pregnant Women and Infants – “Birth Cohort 1”** |
| --- | --- |
| ClinicalTrials.gov Identifier: | NCT02163447 |
| SAP VERSION | 1.0 |
| SAP VERSION DATE | 24-May-2017 |
| TRIAL STATISTICIAN | Edwin D. Charlebois, MPH PhD |
| TRIAL PRINCIPAL INVESTIGATORS | Grant Dorsey, MD, PhD  Moses Kamya, MBChB, MMed, PhD  Diane Havlir, MD  Maggie Feeney, MD, MSc |
| SAP AUTHOR | Edwin D. Charlebois, MPH PhD  Prasanna Jagannathan, MD  Abel Kakuru, MBChB, MSc  Mary K Muhindo, MBChB, MSc |

# Table of Contents

Table of Contents 2

1 Abbreviations and Definitions 5

2 Introduction 6

2.1 Preface 6

2.2 Purpose of the analyses 9

2.3 General Study Design and Plan 9

3 Study Objectives, Analysis Populations, and Endpoints 10

3.1 Study Objectives 10

3.1.1 Objective 1 10

3.1.2 Objective 2 10

3.2 Analysis Populations 11

3.2.1 Full Analysis Population 11

3.2.2 Per Protocol Population 12

3.3 Endpoints 12

3.3.1 Primary outcomes for objective 1 12

3.3.2 Primary outcome for objective 2 12

3.3.3 Secondary Outcomes 13

4 Study Methods 13

4.1 Equivalence or Non-Inferiority Studies 13

4.2 Inclusion-Exclusion Criteria and General Study Population 13

4.2.1 Inclusion Criteria 13

4.2.2 Exclusion criteria 14

4.3 Randomization and Blinding 14

4.4 Study Variables 15

5 Sample Size 16

6 General Considerations 18

6.1 Timing of Analyses 18

6.2 Covariates and Subgroups 19

6.3 Missing Data 19

6.4 Interim Analyses and Data Monitoring 19

6.4.1 Purpose of Interim Analyses 19

6.4.2 Planned Schedule of Interim Analyses 19

6.4.3 Scope of Adaptations 20

6.4.4 Stopping Rules 20

6.4.5 Interim Analysis for Sample Size Adjustment 20

6.4.6 Practical Measures to Minimize Bias 20

6.4.7 Documentation of Interim Analyses 21

6.5 Multi-center Studies 21

7 Summary of Study Data 21

7.1 Trial Profile 21

7.2 Baseline Characteristics 23

7.3 Treatment Adherence 24

8 Efficacy Analyses 26

8.1 Efficacy outcomes 26

8.2 Primary Efficacy Analysis 33

8.2.1 Objective 1 33

8.2.2 Objective 2 34

8.3 Secondary Efficacy Analyses 35

8.3.1 Objective 1 35

8.3.2 Objective 2 36

9 Safety and Tolerability Analyses 43

9.1 Adverse Events 43

9.2 Serious Adverse Events 43

9.3 Analytical Methods 44

10 References 47

# Abbreviations and Definitions

| ACT | Artemisinin-based combination therapy |
| --- | --- |
| AE | Adverse Event |
| AL | Artemether-lumefantrine |
| ALT | Alanine transaminase (SGPT) |
| CAB | Community advisory board |
| CBC | CBC Complete blood cell |
| CRF | Case Report Form |
| DP | Dihydroartemisinin-piperaquine |
| DSMB | Data and Safety Monitoring Board |
| IDRC | Infectious Diseases Research Collaboration |
| IPT | Intermittent preventive therapy |
| IPTp | Intermittent preventive therapy in pregnancy |
| IRB | Institutional review board |
| ITN | Insecticide treated net |
| MOH | Ministry of Health |
| MU | Makerere University |
| NICHD | National Institute of Child Health and Human Development |
| NIH | National Institute of Health |
| SAE | Serious adverse event |
| SAP | Statistical Analysis Plan |
| SP | Sulfadoxine-pyrimethamine |
| TDH | Tororo District Hospital |
| UCSF | University California San Francisco |
| WHO | WHO World Health Organization |

#

# Introduction

## Preface

**Impact of malaria prevention in utero on the risk of malaria in infants.** Malaria during pregnancy remains a significant cause of morbidity and mortality in sub-Saharan Africa[^1^](#_ENREF_1), and is estimated to cause low birth weight in up to 20% of deliveries and more than 100,000 infant deaths annually[^2-4^](#_ENREF_2). To prevent malaria during pregnancy, the World Health Organization recommends routine implementation of malaria-preventive measures, including intermittent preventative therapy in pregnancy (IPTp) with sulfadoxine-pyrimethamine (SP) in all countries in Africa where *P. falciparum* remains endemic. Given reports that IPTp-SP is no longer effective due to widespread drug resistance [^5^](#_ENREF_5)^,^[^6^](#_ENREF_6) we and others are evaluating the efficacy and safety of newer, highly effective artemisinin-based combination therapies such as dihydroartemisinin-piperaquine (IPTp-DP) to prevent malaria during pregnancy[^7^](#_ENREF_7)^,^[^8^](#_ENREF_8). However, an important but unanswered question regarding new interventions to prevent malaria in pregnancy is the impact such interventions might have on the risk of malaria during infancy.

Increasing evidence suggests that maternal infection during pregnancy affects the developing immune system of fetuses independent of potential vertical transmission of pathogens.[^9^](#_ENREF_9) Several studies have suggested that placental malaria is associated with altered parasite-specific immune responses in neonates that could affect response to malaria after birth.[^10-13^](#_ENREF_10) In addition, several clinical studies have reported that infants born to mothers with placental malaria have a higher risk of death [^14^](#_ENREF_14) and malaria during infancy.[^15-18^](#_ENREF_15) However, all of these were observational studies in which it is very difficult to control for exposure, which is tightly linked between pregnant women and their infants. It remains unclear whether, as we hypothesize in this proposal, prevention of malaria during pregnancy improves the development of antimalarial immunity and reduces the risk of malaria after birth.

**Preventing malaria in infants and young children.** Partial immunity to malaria develops through repeated exposure, leading first to protection against severe forms of disease, followed by protection against symptomatic illness.[^19^](#_ENREF_19) Thus, the burden of malaria in Africa is heavily borne by young children. Newborns are protected during the first few months of life, likely due to the transplacental acquisition of maternal antibodies and relatively high fetal hemoglobin content.[^20^](#_ENREF_20) After about 2-3 months of age, protection from these factors wanes.[^21^](#_ENREF_21) However, the age at which malaria risk peaks in endemic areas of Africa varies from 1-2 years of age in areas of high transmission intensity to approximately 5 years of age in areas of low to moderate transmission intensity.[^22^](#_ENREF_22)

The only widely used tool for the prevention of malaria in African children is ITNs, however, there is concern for diminishing efficacy of ITNs due to the alarming emergence of vector resistance to pyrethroids (currently the only class of insecticides used in ITNs) in Uganda [^23^](#_ENREF_23)^,^[^24^](#_ENREF_24) and other parts of Africa.[^25^](#_ENREF_25)^,^[^26^](#_ENREF_26) Our collaboration has been following a cohort of 350 children since 2007 in Tororo, the site of our proposed trial. To date, children between 6-24 months of age have suffered over 5 episodes of malaria per year, and between 2008-11 the incidence of malaria increased by over 50%, despite the use of ITNs.[^27^](#_ENREF_27) Extending the use of IPT to African infants and children at high risk for malaria offers a potential new preventive tool that has recently received widespread attention. The intervention that has been most extensively studied is SP given at the time of routine vaccination in infants (termed IPTi). A recent pooled analysis of 6 RCTs reported that IPTi was safe and associated with a modest 30% protective efficacy against clinical malaria in the first year of life.[^28-33^](#_ENREF_28) In a study from Kenya, IPTi with SP + AS or amodiaquine (AQ) + artesunate was associated with a 22% and 25% protective efficacy against malaria, respectively.[^34^](#_ENREF_34) A different approach to IPT has been taken in parts of West Africa, where the main burden of malaria is in older children and transmission is highly seasonal. In this setting, studies of IPT (termed seasonal malaria chemoprevention or SMC) have delivered drugs at monthly intervals during the transmission season, targeting children under 5 years of age. In two such studies using SP+AQ, the protective efficacy ranged from 70-82%.[^35^](#_ENREF_35)^,^[^36^](#_ENREF_36) In a systematic review and meta-analysis of 12 studies, monthly SMC was safe , with an overall protective efficacy of 82% against clinical malaria and 57% against all-cause mortality.[^37^](#_ENREF_37) SMC has now been recommended as a new malaria control strategy following a meeting of the Malaria Policy Advisory Committee to the WHO in early 2012.[^38^](#_ENREF_38) However, this recommendation only pertains to areas with seasonal malaria transmission, and so not to the vast parts of Africa, such as Uganda, with year-round transmission. In addition, good efficacy for SMC with SP+AQ has been documented in regions of West Africa with considerably lower prevalence of resistance to both SP and AQ than is the case in many other areas, including Uganda.

Though IPT offers great promise for reducing the burden of malaria in African infants and children, interventions must be carefully chosen based on drug resistance patterns and the local epidemiology of disease. IPTi with SP has been associated with modest protective efficacy, however, the WHO recommends IPTi only in countries with moderate to high malaria transmission, where parasite resistance to SP is low.[^39^](#_ENREF_39) SMC using monthly dosing with primarily SP containing combination therapies has been highly efficacious in areas of West Africa where malaria transmission is seasonal, however, this strategy would not be appropriate in most areas of Central and East Africa were transmission is perennial and SP resistance widespread. DP is a new co-formulated ACT that has been suggested as an excellent candidate for IPT in infants and children.[^20^](#_ENREF_20) Several studies from Africa have demonstrated that DP is safe and highly efficacious for the treatment of uncomplicated malaria and has the added benefit over other ACTs in terms of prolonged post-treatment prophylaxis.[^40-43^](#_ENREF_40) DP is now one of the WHO recommended 1st line treatments for malaria and was approved for use in Uganda in 2005. In the only published randomized, placebo-controlled trial of DP for the prevention of malaria conducted in healthy adult Thai males, the protective efficacy of DP over a 9 month period was 98% when the drug was given monthly and 86% when the drug was given every 2 months. [^44^](#_ENREF_44) In an ongoing study carried out by our group, DP every 4 weeks given to infants between the ages of 6-24 months has been highly efficacious for the prevention of malaria in a high transmission setting of Uganda[^45^](#_ENREF_45).

There is conflicting evidence on the impact of chemoprevention in children on the development of antimalarial immunity and the risk of malaria after chemoprevention has stopped. Studies from Tanzania and the Gambia reported that children receiving highly effective chemoprophylaxis had a higher incidence of malaria compared to those receiving placebo in the year following the intervention.[^46^](#_ENREF_46)^,^[^47^](#_ENREF_47) In contrast, more recent studies have reported no association between IPTi with SP and the risk of malaria following the intervention.[^28^](#_ENREF_28)^,^[^34^](#_ENREF_34) Differences in these findings could be due to differences in transmission intensity, ages of study subjects, protective efficacy of study drugs, and dosing strategies (continuous prophylaxis vs. intermittent therapy). Recent data from our group suggest that in a high transmission setting, chemoprevention may actually improve the development of antimalarial immunity. Children highly adherent to DP every 4 weeks had a 97% reduction in the incidence of malaria during the time the intervention was given [^48^](#_ENREF_48) and a 55% reduction in the incidence of malaria in the 1 year after the intervention was stopped [^49^](#_ENREF_49). This may be due to improved priming of cellular immune responses[^49^](#_ENREF_49), as has been observed in several animal and experimental models where parasitemia is suppressed with drugs that are active only against erythrocytic stages.[^50-54^](#_ENREF_50)

## Purpose of the analyses

This proposal will be the first clinical trial we are aware of to evaluate the impact of different IPTp regimens on the risk of malaria in infancy and early childhood. This proposal will also be the first clinical trial to evaluate the efficacy and safety of DP given every 4 weeks vs every 12 weeks for the prevention of malaria in a birth cohort of children born to mothers who received IPTp during pregnancy. We will perform a double-blinded randomized controlled trial where children were randomized in utero to one of 5 different interventions to 1) Compare the risk of malaria from birth to 2 years of age among children whose mothers were randomized to different IPTp regimens, and 2) Compare the risk of malaria in children randomized to DP every 4 weeks vs. every 12 weeks from 2 months to 2 years of age, assessing the risk both during the intervention and for 1 year after stopping the intervention. The primary outcome will be the incidence of malaria.

## General Study Design and Plan

(ICH E3;9)

This is a double-blinded randomized controlled phase III trial of 300 HIV uninfected pregnant women and the children born to them. The study interventions will be divided into two phases. In the first phase, HIV uninfected women enrolled at 12-20 weeks gestation were randomized in equal proportions to one of three IPTp treatment arms: 1) 3 doses of SP, 2) 3 doses of DP, or 3) DP every 4 weeks. All three interventions arms will have either SP or DP placebo to ensure adequate blinding is achieved in the study. Follow-up for the pregnant women will end approximately 6 weeks after giving birth. In the second phase of the study, all children born to pregnant women enrolled in the study will be followed from birth until they reach 36 months of age. Children born to women randomized to receive 3 doses of SP during pregnancy will receive DP every 12 weeks between 2-24 months of age. Children born to women randomized to receive 3 doses of DP or DP every 4 weeks during pregnancy will receive either DP every 12 weeks or DP every 4 weeks between 2-24 months of age. To ensure adequate blinding, children who will receive DP every 12 weeks will be given DP placebo during the months they will not be taking DP. Children will then be followed an additional year between 24-36 months of age following the interventions. The 5 treatment arms are described in Table 1.

Phase I of this study is complete[^55^](#_ENREF_55). The details outlined in this statistical analyses plan will focus on Phase II of the study involving children.

**Table 1. Treatment arms with assignment of study drugs during pregnancy and infancy**

| **Phase of intervention** | **Treatment arm (target number)** | | | | | |
| --- | --- | --- | --- | --- | --- | --- |
|  | **A** | **B** | **C** | | **D** | **E** |
| During pregnancy | SP every 8 weeks (100) | DP every 8 weeks (100) | | | DP every 4 weeks (100) | |
| During infancy | DP every 12 weeks (100) | DP every 12 weeks (50) | | DP every 4 weeks (50) | DP every 12 weeks (50) | DP every 4 weeks (50) |

# Study Objectives, Analysis Populations, and Endpoints

## Study Objectives

(ICH E3; 8.)

### Objective 1

To compare the incidence of malaria among infants whose mothers were randomized to different IPTp regimens. For this objective, only infants randomized to receive DP every 12 weeks during infancy will be included. We will test the hypotheses that infants born to mothers randomized to receive IPTp with DP every 4 weeks or every 8 weeks will have a lower incidence of malaria during the first 24 months of life compared to infants born to mothers who were randomized to receive IPTp with SP every 8 weeks. Secondary outcomes will include the incidence of complicated malaria, hospitalizations, and adverse events, and the prevalence of Plasmodium infection and anemia.

### Objective 2

To compare the incidence of malaria among infants randomized to receive DP every 12 weeks vs. every 4 weeks between 2-24 months of age. We will test the hypothesis that A) infants randomized to receive DP every 4 weeks between 2-24 months of age will have a lower incidence of malaria during the first 24 months of life compared to infants randomized to receive DP every 12 weeks, and B) infants randomized to receive DP every 4 weeks between 2-24 months of age will have a lower incidence of malaria between 24-36 months of age after the intervention is stopped compared to infants randomized to receive DP every 12 weeks between 2-24 months of age. Secondary outcomes will include the incidence of complicated malaria, hospitalizations, and adverse events, and the prevalence of Plasmodium infection and anemia.

## Analysis Populations

(ICH E3; 9.7.1, 11.4.2.5. ICH E9; 5.2)

A modified intention-to-treat approach will be used for all analyses. For objective 1 (impact of IPTp on risk of malaria in infancy), this includes all children born (excluding stillbirths) and randomized to DP every 12 weeks with at least 1 day of evaluable person-time of follow-up. For objective 2, hypothesis 2a (impact of IPT with DP every 4 weeks vs every 12 weeks in preventing malaria in childhood), the analysis will include all children whose mothers received IPT during pregnancy (IPTp) with DP, who received at least one dose of study drugs, and who have evaluable data on specific outcomes regardless of whether the intervention was not given for any reason. For objective 2, hypothesis 2b (impact of childhood IPT on risk of malaria after cessation of intervention), the analysis will include all children studied in hypothesis 2a who have reached 2 years of age and have evaluable person time of follow-up after 2 years of age, regardless of whether the intervention was not given for any reason.

### Full Analysis Population

- *Objective 1: All children born (excluding stillbirths) and randomized to DP every 12 weeks with evaluable person-time of follow-up*
- *Objective 2 Hypothesis 2a: All children who received at least one dose of study drugs and whose mothers received IPTp-DP (either IPTp-DP every 4 weeks or IPTp-DP every 8 weeks)*
- *Objective 2 Hypothesis 2b: All children whose mothers received IPTp-DP who received at least one dose of study drugs and have evaluable person-time of follow-up after 2 years of age*

**Table 2. Analysis Populations**

| **Objective** | **Hypothesis** | **Analysis population** | **Control group** | **Comparison group** |
| --- | --- | --- | --- | --- |
| 1 | 1 | Infants 0-24 months of age randomized to DP every 12 weeks | Infants of women assigned SP every 8 weeks | Infants of women assigned DP every 8 weeks or DP every 4 weeks |
| 2 | 2a | Infants 0-24 months of age born to women assigned to receive IPTp with DP every 8 weeks or DP every 4 weeks | Infants assigned DP every 12 weeks | Infants assigned DP every 4 weeks |
| 2 | 2b | Infants 24-36 months of age born to women assigned to receive IPTp with DP every 8 weeks or DP every 4 weeks | Infants assigned DP every 12 weeks | Infants assigned DP every 4 weeks |

### Per Protocol Population

A per protocol analysis is not planned.

## Endpoints

(ICH E9; 2.2.2)

### Primary outcomes for objective 1

The primary outcome will be the incidence of malaria, defined as the number of incident episodes per time at risk. Incident cases will include all treatments for malaria not proceeded by another treatment less than 14 days prior. Time at risk will begin at birth and will end when study participants reach 24 months of age or early study termination (if prior to 24 months of age).

### Primary outcome for objective 2

The primary outcome will be the incidence of malaria, defined as the number of incident episodes per time at risk. Incident cases will include all treatments for malaria not proceeded by another treatment less than 14 days prior. For hypothesis 2a, time at risk will begin at the time of first study drug dispensation (2 months of age) and will end when study participants reaches 24 months of age, when the intervention will be stopped, or early study termination (if prior to 24 months of age). For hypothesis 2b, time at risk will begin at 24 months of age and will end when study participants reach 36 months of age or early study termination (if between 24-36 months of age).

### Secondary Outcomes (objectives 1 and 2)

- **Incidence of complicated malaria**: Any treatment for malaria meeting criteria for severe malaria or danger sings
- **Incidence of hospital admissions and/or deaths**: Admission to the pediatric ward for any cause**,** and deaths of any cause
- **Incidence of non**-**malarial febrile illness**: Any presentation with fever without parasites detected by blood smear.
- **Prevalence of parasitemia**: Proportion of routine samples (LAMP or blood smears) positive for asexual parasites.
- **Parasite densities if parasitemic:** Parasite densities given the presence of positive blood smear
- **Prevalence of anemia**: Proportion of routine hemoglobin measurements < 11 g/dL & < 8 g/dL
- **Immunologic measurements (antibody responses, cellular responses)** measured at birth and 6, 12, 24 months of age
- **Incidence of adverse event**: Adverse events stratified by type, severity score and relationship to study drugs.

# Study Methods

## Equivalence or Non-Inferiority Studies

(ICH E3; 9.2, 9.7.1, 11.4.2.7. ICH E9; 3.3.2)

N/A - This trial is designed as a superiority trial.

## Inclusion-Exclusion Criteria and General Study Population

(ICH E3;9.3. ICH E9;2.2.1)

Inclusion and exclusion criteria for enrollment in the study were assessed during pregnancy.

### Inclusion Criteria

1) Pregnancy confirmed by positive urine pregnancy test or intrauterine pregnancy by ultrasound

2) Estimated gestational age between 12-20 weeks

3) Confirmed to be HIV uninfected by rapid test

4) 16 years of age or older

5) Residency within 30km of the study clinic

6) Provision of informed consent by the pregnant woman for herself and her unborn child

7) Agreement to come to the study clinic for any febrile episode or other illness and avoid medications given outside the study protocol

8) Plan to deliver in the hospital

### Exclusion criteria

1) History of serious adverse event to SP or DP

2) Active medical problem requiring inpatient evaluation at the time of screening

3) Intention of moving more than 30km from the study clinic

4) Chronic medical condition requiring frequent medical attention

5) Prior SP preventive therapy or any other antimalarial therapy during this pregnancy

6) Early or active labor (documented by cervical change with uterine contractions)

## Randomization and Blinding

(ICH E3; 9.4.3, 9.4.6. ICH E9; 2.3.1, 2.3.2)

There will be 5 treatment arms which include both the intervention for the woman during pregnancy and her unborn child(ren) during infancy (Table 1). Non-singleton births from the same mother will be assigned to the same intervention. We will use a 2:1:1:1:1 randomization scheme targeting 100, 50, 50, 50, 50 pregnant women in treatment arms A-E respectively. This randomization scheme will result in a target of 100 women for each of the 3 different treatment arms during pregnancy. A randomization list will be computer generated by a member of the project who will not be directly involved in the conduct of the study. The randomization list will include consecutive treatment numbers with corresponding random treatment assignments. Randomized codes will correspond to the 5 treatment groups using permuted variable sized blocks of 6 and 12 (to account for treatment group A being represented twice as often as the other 4 treatment groups). Sealed copies of the original randomization list and documentation of the procedure used to generate the lists will be stored in the project administrative offices in San Francisco and Kampala. Prior to the onset of the study, a set of sequentially numbered, opaque, sealed envelopes will be prepared. Each envelope will be marked on the outside with the treatment allocation number. The inside of the envelope will contain a piece of paper with the treatment allocation number and treatment group assignment along with a piece of carbon paper.

## Study Variables

(ICH E3; 9.5.1. ICH E9; 2.2.2)

See Data Dictionary Excel file for complete list of study variables.

**Table 3: Schedule of routine assessments and procedures in children**

| **Weeks of age** | Blood collected by finger prick | | | Blood collected by phlebotomy for CBC, and immunology studies | Blood collected by phlebotomy for ALT | Routine assessment in the study clinic | Administration of study drugs |
| --- | --- | --- | --- | --- | --- | --- | --- |
|  | Blood smear | Dried blood spots | Plasma |  |  |  |  |
| **4** | X | X | X |  |  | X |  |
| **8** | X | X | X | X | X | X | X |
| **12** | X | X | X |  |  | X | X |
| **16** | X | X | X |  |  | X | X |
| **20** | X | X | X |  |  | X | X |
| **24** | X | X | X | X | X | X | X |
| **28** | X | X | X |  |  | X | X |
| **32** | X | X | X |  |  | X | X |
| **36** | X | X | X |  |  | X | X |
| **40** | X | X | X | X | X | X | X |
| **44** | X | X | X |  |  | X | X |
| **48** | X | X | X |  |  | X | X |
| **52** | X | X | X |  |  | X | X |
| **56** | X | X | X | X | X | X | X |
| **60** | X | X | X |  |  | X | X |
| **64** | X | X | X |  |  | X | X |
| **68** | X | X | X |  |  | X | X |
| **72** | X | X | X | X | X | X | X |
| **76** | X | X | X |  |  | X | X |
| **80** | X | X | X |  |  | X | X |
| **84** | X | X | X |  |  | X | X |
| **88** | X | X | X | X | X | X | X |
| **92** | X | X | X |  |  | X | X |
| **96** | X | X | X |  |  | X | X |
| **100** | X | X | X |  |  | X | X |
| **104** | X | X | X | X | X | X | X |
| **108** | X | X |  |  |  | X |  |
| **112** | X | X |  |  |  | X |  |
| **116** | X | X |  |  |  | X |  |
| **120** | X | X |  | X |  | X |  |
| **124** | X | X |  |  |  | X |  |
| **128** | X | X |  |  |  | X |  |
| **132** | X | X |  |  |  | X |  |
| **136** | X | X |  | X |  | X |  |
| **140** | X | X |  |  |  | X |  |
| **144** | X | X |  |  |  | X |  |
| **148** | X | X |  |  |  | X |  |
| **152** | X | X |  |  |  | X |  |
| **156** | X | X |  | X |  | X |  |

# Sample Size

(ICH E3; 9.7.2. ICH E9; 3.5)

The number of pregnant women enrolled and the number of study participants reaching the various endpoints determined the samples sizes for each of the primary outcomes of our study aims (Table 4). The 3 primary hypotheses for phase II of the study are as follows: 1) We will test the hypotheses that infants born to mothers randomized to receive IPTp with DP every 8 weeks or DP every 4 weeks will have a lower incidence of malaria during the first 24 months of life compared to infants born to mothers who were randomized to receive IPTp with SP every 8 weeks. 2a) We will test the hypotheses that infants randomized to receive DP every 4 weeks between 2-24 months of age will have a lower incidence of malaria during the first 24 months of life compared to infants randomized to receive DP every 12 weeks. 2b) We will test the hypothesis that infants randomized to receive DP every 4 weeks between 2-24 months of age will have a lower incidence of malaria between 24-36 months of age after the intervention is stopped compared to infants randomized to DP every 12 weeks between 2-24 months of age. The primary determinant of our target sample size in the complete study was based on testing hypotheses 1 given that the magnitude of differences anticipated for this hypothesis would be smaller than those anticipated for hypotheses 2 and 3.

We conservatively estimate that we will lose 5% of follow-up time per year in the infants. The minimum relative differences detectable for the primary outcomes of the 3 hypotheses given our estimated sample sizes are summarized in Table 5 below. For hypothesis 1, tested among children assigned to receive DP every 12 weeks, we will be powered to detect a 22-28% relative difference in the incidence of malaria between 0-24 months of age between children born to women who received SP every 8 weeks during pregnancy to those born to women who received DP every 4 or 8 weeks during pregnancy, assuming an incidence of malaria in the control group ranging from 3-5 episodes PPY. For hypothesis 2a, we will be powered to detect an 18-23% relative difference in the incidence of malaria between 0-24 months of age among children randomized to DP every 4 weeks compared to children randomized to DP every 12 weeks, assuming an incidence of malaria in the DP every 12 weeks arm ranging from 3-5 episodes PPY. For hypothesis 2b, we will be powered to detect a 19-24% relative difference in the incidence of malaria between 24-36 months of age among children assigned to receive DP every 4 weeks during infancy compared to those who received DP every 12 weeks during infancy assuming an incidence of malaria in the control group ranging from 3-5 episodes PPY.

**Table 4. Intervention arms during pregnancy and infancy**

| **Study populations** | **Intervention arms** | | | | |
| --- | --- | --- | --- | --- | --- |
| Assigned treatment arms in pregnant women | SP every 8 weeks | DP every 8 weeks | | DP every 4 weeks | |
| Estimated number of pregnant women enrolled | 100 | 100 | | 100 | |
| Estimated number with placental outcomes | 90 | 90 | | 90 | |
| Assigned treatment arms in infants | DP every 12 weeks | DP every 12 weeks | DP every 4 weeks | DP every 12 weeks | DP every 4 weeks |
| Estimated number of infants enrolled | 90 | 45 | 45 | 45 | 45 |
| Estimated number of infants reaching 24 months of age | 81 | 41 | 41 | 41 | 41 |

**Table 5. Minimum relative differences in outcomes detectable given estimated effective sample sizes**

| **Objective** | **Hypothesis** | **Analysis population** | **Control group and**  **estimated sample size** | **Comparison group and estimated sample size** | **Estimated outcome measure in control group** | **Minimum relative difference detectable*** |
| --- | --- | --- | --- | --- | --- | --- |
| 1 | 1 | Infants 0-24 months of age randomized to DP every 12 weeks | Infants of women assigned IPTp-SP every 8 weeks (n=90) | Infants of women assigned IPTp-DP every 4 or 8 weeks (n=45 each) | Incidence of malaria =  3-5 episodes PPY | 22-28% |
| 2 | 2a | Infants 0-24 months of age born to women assigned to receive IPTp-DP every 4 or 8 weeks | Infants assigned DP every 12 weeks (n=90) | Infants assigned DP every 4 weeks (n=90) | Incidence of malaria =  3-5 episode PPY | 18-23% |
| 2 | 2b | Infants 24-36 months of age born to women assigned to receive IPTp-DP every 4 or 8 weeks | Infants assigned DP every 12 weeks (n=82) | Infants assigned DP every 4 weeks (n=82) | Incidence of malaria =  3-5 episodes PPY | 19-24% |

* Relative difference = (estimated outcome in control arm – estimated outcome in the comparison arm) / estimated outcome on the control arm (two-sided alpha = 0.05, power = 80%).

# General Considerations

## Timing of Analyses

The final trial analysis for Phase I of the study was completed in 2015. The final analysis for Phase II, objective 1 of the study will be performed after the last child has turned 2 years. The final analysis for Phase II, objective 2 of the study will be performed after the last child has turned 3 years. Prior to the final analysis, for children between 0 to 24 months of age, we will perform an interim safety analysis when ½ of the anticipated total observation time has been accrued. The interim safety analyses will compare the incidence rate ratio of significant adverse events (grade 3/4 & SAEs).

## Covariates and Subgroups

(ICH E3; 9.7.1, 11.4.2.1. ICH E9; 5.7)

We will assess for significant interaction between the primary outcomes and the following potential effect modifiers: sex of the infant, age of the infant, maternal gravidity, and the proportion of time of the mother’s pregnancy under protection of IRS. If there is evidence for significant interaction, we will report results from stratified analyses.

## Missing Data

(ICH E3; 9.7.1, 11.4.2.2. ICH E9;5.3. EMA Guideline on Missing Data in Confirmatory Clinical Trials)

Children in the trial who are prematurely withdrawn from the study will only provide data up until the time of withdrawal.

## Interim Analyses and Data Monitoring

(ICH E3; 9.7.1, 11.4.2.3. ICH E9; 4.1, FDA Feb 2010 “Guidance for Industry Adaptive Design Clinical Trials for Drugs and Biologics”)

### Purpose of Interim Analyses

We will perform an interim safety analysis in addition to a final safety analysis for a total of two sequential evaluations of study safety. The interim safety analyses will compare the incidence rate ratio of significant adverse events (grade 3/4 & SAEs).

### Planned Schedule of Interim Analyses

The interim safety analysis for children will be performed when ½ of the anticipated total observation time has been accrued for children between 0 to 24 months of age. A standardized test statistic will be calculated for the adverse event incidence rate ratio. If this statistic exceeds the nominal critical value calculated using the error spending function (Table 6), then a statistically significant result will have been achieved at the time of the analysis. In that event, the sponsor will be notified and a report submitted for review by the Data Safety Monitoring Board (DSMB). For the interim safety analysis, the study team will present information on recruitment and the results of interim safety analyses to the DSMB, which will review the data and recommend a course of action.

| **Table 6. Schedule of interim safety analysis and boundaries to monitor study outcome** | | | | |
| --- | --- | --- | --- | --- |
| **Number of Evaluable Subjects Accrued or % of Total Accrual Time** | **Test Statistic** | | **Alpha** | **Cumulative Alpha** |
|  | **Lower Bound** | **Upper Bound** |  |  |
| N=145 or 50% of accrual time | -2.51 | 2.51 | 0.00601 | 0.01210 |
| N=290 or 100% of accrual time | -1.99 | 1.99 | 0.02313 | 0.05000 |

This analysis assumes α=0.05 (two-sided test), O’Brien-Fleming boundaries (DeMets error-spending function) and 300 trial participants. We will utilize Programs for Computing Group Sequential Boundaries Using the Lan-DeMets Method.

### Scope of Adaptations

At the time of the interim analysis, the DSMB may decide to continue, stop, or modify the trial based on the interim safety analysis. This may include the discontinuation of a study arm and re-randomization or cessation of subject participation in the stopped arm.

### Stopping Rules

The DSMB will determine whether to stop the study for early evidence of intervention safety problems after a thorough review of interim data. Interim reports will provide cumulative enrollment figures and cumulative adverse birth outcomes, serious adverse events (classified according to grade), sorted by study arm. Brief clinical descriptions of key events will also be provided. The PI will be responsible for immediately reporting to the funding agency any temporary or permanent suspension of the project and the reason for the suspension.

### Interim Analysis for Sample Size Adjustment

The sample size will not be adjusted based on the results of the interim analysis.

### Practical Measures to Minimize Bias

The study will establish and control who will have access to what information at each stage of the trial. Uncontrolled reporting of interim analyses to study investigators responsible for recruiting subjects will not occur.

The following measure will be taken to minimize bias:

- Only the study statistician and assistant statistician will perform the interim analysis.
- Only the statisticians and the DSMB will see any data or analyses at the interim analysis
- No information will be publically available following an interim analysis
- Information will be provided to the sponsor and investigators as per recommendation of the study DSMB.
- Only the statisticians will be unblinded at for the interim analysis

### Documentation of Interim Analyses

Snapshots of the data available at each interim analysis will be preserved, as will all documentation of analysis plans, programming code and reporting provided at the interim analysis.

## Multi-center Studies

(ICH E3;9.7.1, 11.4.2.4. ICH E9; 3.2)

This is a single center study.

# Summary of Study Data

All continuous variables will be summarized using the following descriptive statistics: n (non-missing sample size), mean, standard deviation, median, maximum and minimum. The frequency and percentages (based on the non-missing sample size) of observed levels will be reported for all categorical measures. All summary tables will be structured with a column for each treatment and will be annotated with the total population size relevant to that table/treatment, including any missing observations.

## Trial Profile

The overall study profile will be presented as a figure following CONSORT guidelines. The total numbers of live children born and followed will be presented. The numbers of children enrolled in each treatment arm and followed through each stage of the trial profile are presented in skeleton Tables 7a and 7b below.

**Table 7a. Trial profile (Objective 1)**

|  | **Mothers IPTp treatment arm** | | |
| --- | --- | --- | --- |
|  | **SP every 8 weeks** | **DP every 8 weeks** | **DP every 4 weeks** |
| Women and unborn children enrolled and randomized | ### | ### | ### |
| Women withdrawn before delivery* | ### | ### | ### |
| Women delivered | ### | ### | ### |
| Children delivered** | ### | ### | ### |
| Child death at delivery*** | ### | ### | ### |
| Children born with at least 1 day of follow-up | ### | ### | ### |
| Withdrawn before 24 months of age* | ### | ### | ### |
| Children followed through 24 months of age | ### | ### | ### |

* Specific reasons for premature study withdrawal will be reported

** Number of twin births will be specified

*** Specific etiologies of death will be reported

**Table 7b. Trial profile (Objective 2)**

|  | **IPT treatment arm** | |
| --- | --- | --- |
|  | **DP every 12 weeks** | **DP every 4 weeks** |
| Women and unborn children enrolled and randomized | ### | ### |
| Women withdrawn before delivery* | ### | ### |
| Women delivered | ### | ### |
| Children delivered** | ### | ### |
| Child death at delivery*** | ### | ### |
| Children born with at least 1 day of follow-up | ### | ### |
| Withdrawn before 1^st^ dose of study drugs* | ### | ### |
| Received at least 1 dose of study drugs | ### | ### |
| Withdrawn between 1^st^ dose of study drugs and 24 months of age* | ### | ### |
| Reached 24 months of age | ### | ### |
| Withdrawn between 24 and 36 months of age* | ### | ### |
| Reached 36 months of age | ### | ### |

* Specific reasons for premature study withdrawal will be reported

** Number of twin births will be specified

*** Specific etiologies of death will be reported

## Baseline Characteristics

Skeleton table of all baseline variables collected on the day of enrollment that will be presented are provided in Table 8a and 8b below.

**Table 8a. Characteristics of children at birth (Objective 1)**

| **Characteristic** | **Mothers IPTp Treatment arm** | | |
| --- | --- | --- | --- |
|  | **SP every 8 weeks**  **(n=XXX)** | **DP every 8 weeks**  **(n=XXX)** | **DP every 4 weeks**  **(n=XXX)** |
| Mother’s gravidity, n (%)  1  2  > 3 | n (%)  n (%)  n (%) | n (%)  n (%)  n (%) | n (%)  n (%)  n (%) |
| Gender (n, % female) | XX (XX-XX) | XX (XX-XX) | XX (XX-XX) |
| Gestational age in weeks at birth, mean (range) | XX (XX-XX) | XX (XX-XX) | XX (XX-XX) |
| Preterm births (<37 weeks of gestation) | n(%) | n(%) | n(%) |
| Birth weight in grams, mean (range) | XX (XX-XX) | XX (XX-XX) | XX (XX-XX) |
| Low birth weight (< 2500 gm), n(%) | n (%) | n (%) | n (%)) |
| Incidence of malaria during mother’s pregnancy^a^ |  |  |  |
| Parasite prevalence during mother’s pregnancy^b^ | n/N (%) | n/N (%) | n/N (%) |
| Evidence of placental malaria by microscopy, n(%) | n (%) | n (%) | n (%) |
| Evidence of placental malaria by LAMP^c^, n(%) | n (%) | n (%) | n (%)) |
| Evidence of placental malaria by histology, n(%) | n (%) | n (%) | n (%)) |

^a^Episodes of malaria per person year at risk

^a^#parasitemic months/total months of observation

^a^Loop amplified isothermal amplification

**Table 8b: Characteristics of children at birth (Objective 2)**

| **Characteristic** | **Treatment arm** | |
| --- | --- | --- |
|  | **DP every 12 weeks**  **(n=XXX)** | **DP every 4 weeks**  **(n=XXX)** |
| Maternal IPTp during pregnancy N(%) |  |  |
| DP every 8 weeks | n (%) | n (%) |
| DP every 4 weeks | n (%) | n (%) |
| Mother’s gravidity, n (%) |  |  |
| 1 | n (%) | n (%) |
| 2  3 | n (%)  n (%) | n (%)  n (%) |
| Gender (n, % female) | XX (XX-XX) | XX (XX-XX) |
| Gestational age in weeks at birth, mean (range) | XX (XX-XX) | XX (XX-XX) |
| Preterm births (<37 weeks of gestation) | n(%) | n(%) |
| Birth weight in grams, mean (range) | XX (XX-XX) | XX (XX-XX) |
| Low birth weight (< 2500 gm), n(%) | n (%) | n (%)) |
| Incidence of malaria during mother’s pregnancy | n (%) | n (%) |
| Parasite prevalence during mother’s pregnancy | n (%) | n (%) |
| Evidence of placental malaria by microscopy, n(%) | n (%) | n (%) |
| Evidence of placental malaria by LAMP^a^, n(%) | n (%) | n (%) |
| Evidence of placental malaria by histology, n(%) | n (%) | n (%) |

^a^Loop amplified isothermal amplification

## Treatment Adherence

During infancy, children will be given 1 of 2 treatment regimens from 8 weeks to 104 weeks of age: 1) DP given every 12 weeks, or 3) DP given every 4 weeks. Each treatment with DP will consist of half-strength tablets given once a day for 3 consecutive days according to weight based guidelines. Infants randomized to receive DP every 12 weeks will receive placebo mimicking the dosing of DP every 4 weeks when they are not receiving study drug. All doses of study drugs will be pre-packaged by a study pharmacist and administered by a study nurse blinded to the study participant’s treatment regimen. For DP (or DP placebo), the first of the 3 daily doses will be directly observed in the clinic and the 2^nd^ and 3^rd^ daily doses will be administered at home using pre-packaged study drugs in opaque envelopes with dosing instructions written on the outside. For doses of study drugs administered in the clinic, if a study participant vomits the study drug within 30 minutes of administration, the drug will be re-administered. For doses of study drugs administered at home, if a study participant vomits the study drug within 30 minutes of administration or study drug is lost, the study participant will be instructed to come to the study clinic as soon as possible where the study drug will be re-administered/replaced. Measures of treatment adherence are summarized in skeleton Table 9 below.

**Table 9. Measures of treatment adherence (objective 2)**

|  | **Treatment arm** | |
| --- | --- | --- |
|  | **DP every 12 weeks** | **DP every 4 weeks** |
| At the level of each individual child receiving at least one dose of study drugs | | |
| At least one dose of study drug held for adverse event | n/N (%) | n/N (%) |
| Missed at least 1 course of study drugs (all 3 doses) | n/N (%) | n/N (%) |
| Reported not taking at least 1 dose of study drug at home | n/N (%) | n/N (%) |
| At the level of each scheduled dose of study drug | | |
| Study drugs (all 3 doses) held for adverse event | n/N (%) | n/N (%) |
| Study drugs (all 3 doses) missed | n/N (%) | n/N (%) |
| Reported not taking day 2 dose of study drugs at home | n/N (%) | n/N (%) |
| Reported not taking day 3 dose of study drugs at home | n/N (%) | n/N (%) |

#

# Efficacy Analyses

## Efficacy outcomes

Definitions and criteria used to generate estimates of all primary and secondary efficacy outcomes are presented in Table 10a, 10b, and 10c below.

**Table 10a. Primary and secondary outcomes (Objective 1)**

| **Outcome** | **Category** | **Type of measurement** | **Timing of measurement** | **Numerator** | **Denominator** | **Missing data** | **Imputation** |
| --- | --- | --- | --- | --- | --- | --- | --- |
| Symptomatic malaria during infancy | Primary outcome | Incidence | Time at risk from birth to 104 weeks of age | Number of incident episodes of fever and positive blood smear by microscopy | Duration of observation from birth to 104 weeks or premature study withdrawal | None | None |
| Time to first episode of malaria | Secondary outcome | Hazard | Time at risk from birth to 104 weeks of age | Cumulative risk of having a first episode of malaria | Days from birth to event, 104 weeks of age, or premature study withdrawal | None | None |
| Complicated malaria during infancy | Secondary outcome | Incidence | Time at risk from birth to 104 weeks of age | Number of episodes of complicated malaria | Duration of observation from birth to 104 weeks or premature study withdrawal | None | None |
| Hospitalizations and/or deaths | Secondary outcome | Incidence | Time at risk from birth to 104 weeks of age | Number of hospitalizations and/or deaths | Duration of observation from birth to 104 weeks or premature study withdrawal | None | None |
| Non-malarial febrile illness | Secondary outcome | Incidence | Time at risk from birth to 104 weeks of age | Number of incident episodes of fever and negative blood smear by microscopy | Duration of observation from birth to 104 weeks or premature study withdrawal | None | None |
| Parasitemia during infancy detected by microscopy and LAMP | Secondary outcome | Proportion | Each 4-week block from birth to 104 weeks of age | Blood samples with parasites detected by blood smear or LAMP at the time of each routine visit + any episode of malaria diagnosed in the previous 28 days | Each 4 week block from 4 weeks to 104 weeks or age with at least one blood smear performed | No blood smear performed during 4-week block | None |
| Parasite density if parasitemic by microscopy | Secondary outcome | Continuous, conditional | Each 4-week block from birth to 104 weeks of age with a positive blood smear | Continuous measurements | N/A | None | None |
| Anemia | Secondary outcome | Proportion | Routine visit with phleobotomy (q16 weeks from 8 weeks to 104 weeks of age) | Hemoglobin level < 11 g/dL and < 8 g/dL | Every routine visit with phlebotomy performed from 8 weeks to 104 weeks of age or premature study withdrawal | Missed routine visits or sample not collected when scheduled for phlebotomy | None |
| Immunologic parameters | Secondary outcome | Continuous measurement | At birth, 6, and 12 months of age | Continuous measurements | N/A | Sample not collected when scheduled for phlebotomy | None |

**Table 10b. Primary and secondary outcomes (Objective 2, hypothesis 2a)**

| **Outcome** | **Category** | **Type of measurement** | **Timing of measurement** | **Numerator** | **Denominator** | **Missing data** | **Imputation** |
| --- | --- | --- | --- | --- | --- | --- | --- |
| Symptomatic malaria during intervention | Primary outcome | Incidence | Time at risk during IPT intervention | Number of incident episodes of fever and positive blood smear by microscopy | Duration of observation from day following 1^st^ dose of study drugs to 104 weeks or premature study withdrawal | None | None |
| Time to first episode of malaria | Secondary outcome | Hazard | Time at risk following study drug administration | Cumulative risk of having a first episode of malaria | Days from study drug administration to event, next study drug administration, 104 weeks of age, or premature study withdrawal | None | None |
| Complicated malaria during intervention | Secondary outcome | Incidence | Time at risk during IPT intervention | Number of episodes of complicated malaria | Duration of observation from day following 1^st^ dose of study drugs to 104 weeks or premature study withdrawal | None | None |
| Hospitalizations and/or deaths during intervention | Secondary outcome | Incidence | Time at risk during IPT intervention | Number of hospitalizations and/or deaths | Duration of observation from day following 1^st^ dose of study drugs to 104 weeks or premature study withdrawal | None | None |
| Gametocytemia during intervention | Secondary outcome | Proportion | Each 4-week block following first study drug administration (8 weeks) to 104 weeks of age | Blood samples with gametocytes detected by blood smear at the time of each routine visit + any episode of malaria diagnosed in the previous 28 days | Each 4 week block from 8 weeks to 104 weeks or age with at least one blood smear performed | No blood smear performed during 4-week block | None |
| Parasitemia during intervention detected by microscopy and LAMP | Secondary outcome | Proportion | Each 4-week block following first study drug administration (8 weeks) to 104 weeks of age | Blood samples with parasites detected by blood smear or LAMP at the time of each routine visit + any episode of malaria diagnosed in the previous 28 days | Each 4 week block from 8 weeks to 104 weeks or age with at least one blood smear performed | No blood smear performed during 4-week block | None |
| Parasite density if parasitemic by microscopy | Secondary outcome | Continuous, conditional | Each 4-week block from 8 weeks to 104 weeks of age with a positive blood smear | Continuous measurements | N/A | None | None |
| Anemia during intervention | Secondary outcome | Proportion | Routine visit with phleobotomy (q16 weeks from 8 weeks to 104 weeks of age) | Hemoglobin level < 11 g/dL and < 8 g/dL | Every routine visit with phlebotomy performed from 8 weeks to 104 weeks of age or premature study withdrawal | Missed routine visits or sample not collected when scheduled for phlebotomy | None |
| Immunologic parameters | Secondary outcome | Continuous measurement | At 24 months of age | Continuous measurements | N/A | Sample not collected when scheduled for phlebotomy | None |

**Table 10c. Primary and secondary outcomes (Objective 2, hypothesis 2b)**

| Symptomatic malaria following intervention | Primary outcome | Incidence | Time at risk after cessation of intervention | Number of incident episodes of fever and positive blood smear by microscopy | Duration of observation from 104 weeks to end of study or premature study withdrawal | None | None |
| --- | --- | --- | --- | --- | --- | --- | --- |
| Time to first episode of malaria following intervention | Secondary outcome | Hazard | Time at risk from 104 weeks of age to 156 weeks | Cumulative risk of having a first episode of malaria | Days from 104 weeks to event, study end, or premature study withdrawal | None | None |
| Complicated malaria following intervention | Secondary outcome | Incidence | Time at risk after cessation of intervention | Number of episodes of complicated malaria | Duration of observation from 104 weeks to end of study or premature study withdrawal | None | None |
| Hospitalizations and/or deaths following intervention | Secondary outcome | Incidence | Time at risk after cessation of intervention | Number of hospitalizations and/or deaths | Duration of observation from 104 weeks to end of study or premature study withdrawal | None | None |
| Parasitemia following intervention detected by microscopy and LAMP | Secondary outcome | Proportion | Each 4 week block from 104 weeks to 156 weeks of age | Blood samples with parasites detected by blood smear or LAMP at the time of each routine visit + any episode of malaria diagnosed in the previous 28 days | Each 4 week block from 104 weeks to 156 weeks or age with at least one blood smear performed | No blood smear performed during 4-week block | None |
| Parasite density following intervention if parasitemic by microscopy | Secondary outcome | Continuous, conditional | Each 4 week block from 104 weeks to 156 weeks of age with a positive blood smear | Continuous measurements | N/A | None | None |
| Anemia following intervention | Secondary outcome | Proportion | Routine visit with phleobotomy (q16 weeks from 104 weeks to 156 weeks of age) | Hemoglobin level < 11 g/dL and < 8 g/dL | Every routine visit with phlebotomy performed from 104 weeks to 156 weeks of age | Missed routine visits or sample not collected when scheduled for phlebotomy | None |

## Primary Efficacy Analysis

### Objective 1

We will test the hypothesis that infants born to mothers randomized to receive IPTp with DP every 8 weeks or every 4 weeks will have a lower incidence of malaria during the first 24 months of life compared to infants born to mothers who were randomized to receive IPTp with SP given every 8 weeks. We will also compare the two different IPTp strategies of DP. Only children randomized to DP every 12 weeks will be included in this analysis since we hypothesized that children randomized to DP every 4 weeks from 8 weeks to 24 months of life would not be at risk for malaria.

**Primary Outcome:** The primary outcome will be the incidence of symptomatic malaria (fever and positive blood smear requiring antimalarial treatment). Incident episodes of malaria will be defined as any treatment for malaria not preceded by another treatment for malaria < 14 days prior.

**Analysis Method:** A modified intention-to-treat approach to all will be used, including all study participants born with at least one day of follow-up.

Primary analysis. We will compare the incidence of symptomatic malaria from birth to 104 weeks of age using Poisson or negative binomial regression models. The Poisson models will include the logarithm of the follow-up time as an offset. We will translate the fitted coefficients and their confidence bounds into percentage effects with the formula 100*[exp(coefficient)-1]. This approach is closely related to exponential survival models for analyzing events per follow-up time, but is better able to adjust for violated assumptions. Testing for overdispersion in the Poisson regression can detect violations of these assumptions, and variances can be adjusted accordingly to produce valid p-values and confidence intervals. If significant deviations from required distributions in study data are detected, we will employ negative-binomial or zero-inflated negative-binomial models to account for the observed pattern of data. If necessary, multivariate analyses will be performed to adjust for potential confounders. Comparisons of incidence measures will be expressed at the incidence rate ratio (IRR) or the protective efficacy (PE = 1-IRR x 100%).

We will explore for any significant interaction between potential effect modifiers (i.e. infant sex, maternal gravidity, proportion of mother’s pregnancy under protection of IRS) between the treatment arms. If evidence of significant interaction is found, stratified results will be reported.

### Objective 2

We will test the hypothesis that a) infants randomized to receive DP every 4 weeks between 2-24 months of age will have a lower incidence of malaria during the first 24 months of life compared to infants randomized to receive DP every 12 weeks, and b) infants randomized to receive DP every 4 weeks between 2-24 months of age will have a lower incidence of malaria between 24-36 months of age after the intervention is stopped compared to infants randomized to DP every 12 weeks between 2-24 months of age. Only children whose mothers were randomized to one of the two IPTp-DP arms will be included in this analysis.

**Primary Outcomes:** The primary outcomes will be the incidence of symptomatic malaria (fever and positive blood smear requiring antimalarial treatment) during the intervention (hypothesis 2a) and following the intervention (hypothesis 2b). Incident episodes of malaria will be defined as any treatment for malaria not preceded by another treatment for malaria within the prior 14 days.

**Analysis Method:** A modified intention-to-treat approach to all will be used, including all study participants randomized to therapy with measureable follow-up during the time at risk, regardless of whether the intervention was not given for any reason.

**Primary analysis.** We will compare the incidence of symptomatic malaria during risk periods using Poisson or negative binomial regression models. The Poisson models will include the logarithm of the follow-up time as an offset. We will translate the fitted coefficients and their confidence bounds into percentage effects with the formula 100*[exp(coefficient)-1]. This approach is closely related to exponential survival models for analyzing events per follow-up time, but is better able to adjust for violated assumptions. Testing for overdispersion in the Poisson regression can detect violations of these assumptions, and variances can be adjusted accordingly to produce valid p-values and confidence intervals. If significant deviations from required distributions in study data are detected, we will employ negative-binomial or zero-inflated negative-binomial models to account for the observed pattern of data. If necessary, multivariate analyses will be performed to adjust for potential confounders. Comparisons of incidence measures will be expressed at the incidence rate ratio (IRR) or the protective efficacy (PE = 1-IRR x 100%).

We will explore for any significant interaction between potential effect modifiers (i.e. infant sex, maternal gravidity, proportion of mother’s pregnancy under protection of IRS) between the treatment arms. If evidence of significant interaction is found, we will report stratified results.

## Secondary Efficacy Analyses

### Objective 1

Secondary Outcomes: Secondary outcomes will include outcomes as listed in Table 10a.

Analysis Method: A modified intention-to-treat approach to all will be used, including all study participants born with at least one day of follow-up.

Secondary analyses. We will compare the cumulative risk of being diagnosed with a first episode of malaria from birth to 104 weeks of age using the Kaplan-Meier product limit formula. Associations between maternal IPTp treatment will be made using Cox Proportional hazards models.

We will compare the incidence of complicated malaria, hospitalizations/deaths, and non-malarial febrile illness from birth to 104 weeks of age using Poisson or negative binomial regression models, as above. If necessary, multivariate analyses will be performed to adjust for potential confounders. Comparisons of incidence measures will be expressed at the incidence rate ratio (IRR) or the protective efficacy (PE = 1-IRR x 100%).

For repeated dichotomous measures in the same study participant (parasitemia and anemia during infancy) we will use generalized estimating equations with a log-binomial family and robust standard errors, and report measures of association as the risk ratio (RR). For repeated continuous measures in the same study participant (e.g. parasite densities if parasitemic) parasite densities will be log-normalized, and .associations with treatment groups assessed using generalized estimating equations with a guassian family and robust standard errors

Immunologic parameters (malaria-specific antibody and cellular responses) will be compared between groups using the non-parametric Wilcoxon-Ranksum test.

### Objective 2

Secondary Outcomes: Secondary outcomes will include pediatric outcomes as listed in Table 10b-c.

Analysis Method: A modified intention-to-treat approach to all will be used, including all study participants randomized to therapy with measureable follow-up during the time at risk, regardless of whether the intervention was not given for any reason.

Secondary analyses. We will compare the cumulative risk of developing malaria from time of study drug administration using the within-subjects variance-corrected cox-proportional hazards model (objective 2a). We will compare the cumulative risk of developing malaria from 104 weeks of age to the end of the study (objective 2b) using the Kaplan-Meier product limit formula. Associations between pediatric IPT treatment will be made using Cox Proportional hazards models.

We will compare the incidence of complicated malaria and hospitalizations/deaths during risk periods using Poisson or negative binomial regression models, as above. If necessary, multivariate analyses will be performed to adjust for potential confounders and effect modifiers. Comparisons of incidence measures will be expressed at the incidence rate ratio (IRR) or the protective efficacy (PE = 1-IRR x 100%).

For repeated dichotomous measures in the same study participant (parasitemia, gametocytemia, and anemia during infancy) we will use generalized estimating equations with a log-binomial family and robust standard errors, and report measures of association as the risk ratio (RR). For repeated continuous measures in the same study participant (e.g. parasite densities if parasitemic) parasite densities will be log-normalized, and associations with treatment groups assessed using generalized estimating equations with a guassian family and robust standard errors

Immunologic parameters (malaria-specific antibody and cellular responses) will be compared between groups using the non-parametric Wilcoxon-Ranksum test.

Skeleton tables for the presentation of primary and secondary efficacy outcomes are presented in Tables 11a, 11b, and 12 below.

**Table 11a. Outcomes assessed during infancy (Objective 1)**

| **Outcome** | **Maternal IPTp treatment arm** | | | | | | |
| --- | --- | --- | --- | --- | --- | --- | --- |
|  | **SP every 8 weeks^a^** | **DP every 8 weeks** | | | **DP every 4 weeks** | | |
| **Incidence measures** | **Events^b^** | **Events^b^** | **IRR (95% CI)** | **p-value** | **Events^b^** | **IRR (95% CI)** | **p-value** |
| Symptomatic malaria | xx (x.xx) | xx (x.xx) |  |  | xx (x.xx) |  |  |
| Complicated malaria | xx (x.xx) | xx (x.xx) |  |  | xx (x.xx) |  |  |
| Hospitalizations/deaths | xx (x.xx) | xx (x.xx) |  |  | xx (x.xx) |  |  |
| Non-malarial febrile illness | xx (x.xx) | xx (x.xx) |  |  | xx (x.xx) |  |  |
| **Time to event measures** | **Cumulative risk** | **Cumulative risk** | **HR (95% CI)** | **p-value** | **Cumulative risk** | **HR (95% CI)** | **p-value** |
| Symptomatic malaria | xx (x.xx) | xx (x.xx) |  |  | xx (x.xx) |  |  |
| **Prevalence measures** | **Prevalence** | **Prevalence** | **RR (95% CI)** | **p-value** | **Prevalence** | **RR (95% CI)** | **p-value** |
| Detection of malaria parasites^c^  All routine visits  0 - ≤4 weeks age  >4 - ≤8 weeks age  >8 - ≤12 weeks age  >12 - ≤16 weeks age  >16 - ≤20 weeks age  >20 - ≤24 weeks age  >24 - ≤28 weeks age  >28 - ≤32 weeks age  >32 - ≤36 weeks age  >36 - ≤40 weeks age  >40 - ≤44 weeks age  >44 - ≤48 weeks age  >48 - ≤52 weeks age  >52 - ≤56 weeks age  >56 - ≤60 weeks age  >60 - ≤64 weeks age  >64 - ≤68 weeks age  >68 - ≤72 weeks age  >72 - ≤76 weeks age  >76 - ≤80 weeks age  >80 - ≤84 weeks age  >84 - ≤88 weeks age  >88 - ≤92 weeks age  >92 - ≤96 weeks age  >96 - ≤100 weeks age  >100 - ≤104 weeks age | n/N (%)  n/N (%)  n/N (%)  n/N (%)  n/N (%)  n/N (%)  n/N (%)  n/N (%)  n/N (%)  n/N (%)  n/N (%)  n/N (%)  n/N (%)  n/N (%)  n/N (%)  n/N (%)  n/N (%)  n/N (%)  n/N (%)  n/N (%)  n/N (%)  n/N (%)  n/N (%)  n/N (%)  n/N (%)  n/N (%)  n/N (%) | n/N (%)  n/N (%)  n/N (%)  n/N (%)  n/N (%)  n/N (%)  n/N (%)  n/N (%)  n/N (%)  n/N (%)  n/N (%)  n/N (%)  n/N (%)  n/N (%)  n/N (%)  n/N (%)  n/N (%)  n/N (%)  n/N (%)  n/N (%)  n/N (%)  n/N (%)  n/N (%)  n/N (%)  n/N (%)  n/N (%)  n/N (%) |  |  | n/N (%)  n/N (%)  n/N (%)  n/N (%)  n/N (%)  n/N (%)  n/N (%)  n/N (%)  n/N (%)  n/N (%)  n/N (%)  n/N (%)  n/N (%)  n/N (%)  n/N (%)  n/N (%)  n/N (%)  n/N (%)  n/N (%)  n/N (%)  n/N (%)  n/N (%)  n/N (%)  n/N (%)  n/N (%)  n/N (%)  n/N (%) |  |  |
| Anemia defined as hemoglobin level < 11 g/dL  All routine visits  8 weeks age  24 weeks age  40 weeks age  56 weeks age  72 weeks age  88 weeks age  104 weeks age | n/N (%)  n/N (%)  n/N (%)  n/N (%)  n/N (%)  n/N (%)  n/N (%)  n/N (%) | n/N (%)  n/N (%)  n/N (%)  n/N (%)  n/N (%)  n/N (%)  n/N (%)  n/N (%) |  |  | n/N (%)  n/N (%)  n/N (%)  n/N (%)  n/N (%)  n/N (%)  n/N (%)  n/N (%) |  |  |

**^a^** Reference group

**^b^** Number of events (incidence per person year at risk)

^c^ By blood smear and/or LAMP

**Table 11b. Outcomes assessed during intervention (Objective 2, hypothesis 2a)**

| **Outcome** | **IPT treatment arm** | | | |
| --- | --- | --- | --- | --- |
|  | **DP every 12 weeks^a^** | **DP every 4 weeks** | | |
| **Incidence measures** | **Events^b^** | **Events^b^** | **IRR (95% CI)** | **p-value** |
| Symptomatic malaria | xx (x.xx) | xx (x.xx) |  |  |
| Complicated malaria | xx (x.xx) | xx (x.xx) |  |  |
| Hospitalizations/deaths | xx (x.xx) | xx (x.xx) |  |  |
| Non-malarial febrile illness | xx (x.xx) | xx (x.xx) |  |  |
| **Time to event measures** | **Cumulative Risk** | **Cumulative Risk** | **HR (95% CI)** | **p-value** |
| Symptomatic malaria | xx (x.xx) | xx (x.xx) |  |  |
| **Prevalence measures** | **Prevalence** | **Prevalence** | **RR (95% CI)** | **p-value** |
| Detection of malaria parasites^c^  All routine visits  >8 - ≤12 weeks age  >12 - ≤16 weeks age  >16 - ≤20 weeks age  >20 - ≤24 weeks age  >24 - ≤28 weeks age  >28 - ≤32 weeks age  >32 - ≤36 weeks age  >36 - ≤40 weeks age  >40 - ≤44 weeks age  >44 - ≤48 weeks age  >48 - ≤52 weeks age  >52 - ≤56 weeks age  >56 - ≤60 weeks age  >60 - ≤64 weeks age  >64 - ≤68 weeks age  >68 - ≤72 weeks age  >72 - ≤76 weeks age  >76 - ≤80 weeks age  >80 - ≤84 weeks age  >84 - ≤88 weeks age  >88 - ≤92 weeks age  >92 - ≤96 weeks age  >96 - ≤100 weeks age  >100 - ≤104 weeks age | n/N (%)  n/N (%)  n/N (%)  n/N (%)  n/N (%)  n/N (%)  n/N (%)  n/N (%)  n/N (%)  n/N (%)  n/N (%)  n/N (%)  n/N (%)  n/N (%)  n/N (%)  n/N (%)  n/N (%)  n/N (%)  n/N (%)  n/N (%)  n/N (%)  n/N (%)  n/N (%)  n/N (%)  n/N (%) | n/N (%)  n/N (%)  n/N (%)  n/N (%)  n/N (%)  n/N (%)  n/N (%)  n/N (%)  n/N (%)  n/N (%)  n/N (%)  n/N (%)  n/N (%)  n/N (%)  n/N (%)  n/N (%)  n/N (%)  n/N (%)  n/N (%)  n/N (%)  n/N (%)  n/N (%)  n/N (%)  n/N (%)  n/N (%) |  |  |
| Anemia defined as hemoglobin level < 11 g/dL  All routine visits  8 weeks age  24 weeks age  40 weeks age  56 weeks age  72 weeks age  88 weeks age  104 weeks age | n/N (%)  n/N (%)  n/N (%)  n/N (%)  n/N (%)  n/N (%)  n/N (%)  n/N (%) | n/N (%)  n/N (%)  n/N (%)  n/N (%)  n/N (%)  n/N (%)  n/N (%)  n/N (%) |  |  |
| Gametocytemia  All routine visits | n/N (%) | n/N (%) |  |  |

**^a^** Reference group

**^b^** Number of events (incidence per person year at risk)

^c^ By blood smear and/or LAMP

**Table 12. Outcomes assessed after intervention (Objective 2, hypothesis 2b)**

| **Outcome** | **IPT treatment arm** | | | |
| --- | --- | --- | --- | --- |
|  | **DP every 12 weeks^a^** | **DP every 4 weeks** | | |
| **Incidence measures** | **Events^b^** | **Events^b^** | **IRR (95% CI)** | **p-value** |
| Symptomatic malaria | xx (x.xx) | xx (x.xx) |  |  |
| Complicated malaria | xx (x.xx) | xx (x.xx) |  |  |
| Hospitalizations/deaths | xx (x.xx) | xx (x.xx) |  |  |
| Non-malarial febrile illness | xx (x.xx) | xx (x.xx) |  |  |
| **Time to event measures** | **Cumulative Risk** | **Cumulative Risk** | **HR (95% CI)** | **p-value** |
| Symptomatic malaria | xx (x.xx) | xx (x.xx) |  |  |
| **Prevalence measures** | **Prevalence** | **Prevalence** | **RR (95% CI)** | **p-value** |
| Detection of malaria parasites^c^  All routine visits  >104 - ≤108 weeks age  >108 - ≤112 weeks age  >112 - ≤116 weeks age  >116 - ≤120 weeks age  >120 - ≤124 weeks age  >124 - ≤128 weeks age  >128 - ≤132 weeks age  >132 - ≤136 weeks age  >136 - ≤140 weeks age  >140 - ≤144 weeks age  >144 - ≤148 weeks age  >148 - ≤152 weeks age  >152 - ≤156 weeks age | n/N (%)  n/N (%)  n/N (%)  n/N (%)  n/N (%)  n/N (%)  n/N (%)  n/N (%)  n/N (%)  n/N (%)  n/N (%)  n/N (%)  n/N (%)  n/N (%) | n/N (%)  n/N (%)  n/N (%)  n/N (%)  n/N (%)  n/N (%)  n/N (%)  n/N (%)  n/N (%)  n/N (%)  n/N (%)  n/N (%)  n/N (%)  n/N (%) |  |  |
| Anemia defined as hemoglobin level < 11 g/dL  All routine visits  120 weeks age  136 weeks age  156 weeks age | n/N (%)  n/N (%)  n/N (%)  n/N (%)  n/N (%) | n/N (%)  n/N (%)  n/N (%)  n/N (%)  n/N (%) |  |  |

**^a^** Reference group

**^b^** Number of events (incidence per person year at risk)

^c^ By blood smear and/or LAMP

# Safety and Tolerability Analyses

Safety and tolerability will be evaluated during the period following the 1^st^ dose of study drug administration through the end of the intervention period (104 weeks of age) or premature study withdrawal.

## Adverse Events

An adverse event will be defined as "any untoward medical occurrence in a patient or clinical investigation subject administered a pharmaceutical product that does not necessarily have a causal relationship with this treatment" (ICH Guidelines E2A). An adverse event can further be broadly defined as any untoward deviation from baseline health, which includes:

- Worsening of conditions present at the onset of the study
- Deterioration due to the primary disease
- Intercurrent illness
- Events related or possibly related to concomitant medications

(International Centers for Tropical Disease Research Network Investigator Manual, Monitoring and Reporting Adverse Events, 2003).

At each scheduled and unscheduled visit to the clinic, study clinicians will assess patients according to a standardized case record form. A severity grading scale, based on toxicity grading scales developed by the NIH Divisions of AIDS (DAIDS) and the Division of Microbiology and Infectious Diseases (DMID) Pediatric Toxicity Tables, will be used to grade severity of all symptoms, physical exam findings, and laboratory results. All participants, regardless of treatment arm, will be assessed using the same standardized case record form. Adverse event monitoring will occur during the period when study drugs are given and up one month after the last dose of study drugs. Data will be captured on the incidence of all adverse events, regardless of severity. For each adverse event identified as severity grade 3-4 or a serious adverse event (SAE), an additional adverse event report form will be completed.

## Serious Adverse Events

A Serious Adverse Event (SAE) will be define as any adverse event that results in any of the following outcomes:

• Death

• Life-threatening adverse experience

• Inpatient hospitalization or prolongation of existing hospitalization

• Persistent or significant disability/incapacity

• Congenital malformation/birth defect

• Any other experience that suggests a significant hazard, contraindication, side effect or precaution that **may require medical or surgical intervention** to prevent one of the outcomes listed above

• Event that changes the risk/benefit ratio of the study

## Analytical Methods

We will test the hypothesis that children who receive IPT with DP every 4 weeks will have a lower incidence of adverse events and better tolerability compared to those who receive DP every 12 weeks. A modified intention-to-treat approach to all will be used, including all study participants who received at least one dose of study drugs, regardless of whether subsequently the intervention was not given for any reason. We will compare the proportions of study participants with vomiting following each dose of study drugs using generalized estimating equations with a log-binomial family and robust standard errors to account for repeated measures in the same study participant. We will compare the incidence of various adverse events using Poisson or negative binomial regression models. The Poisson models will include the logarithm of the follow-up time as an offset. We will translate the fitted coefficients and their confidence bounds into percentage effects with the formula 100*[exp(coefficient)-1]. This approach is closely related to exponential survival models for analyzing events per follow-up time, but is better able to adjust for violated assumptions. Testing for overdispersion in the Poisson regression can detect violations of these assumptions, and variances can be adjusted accordingly to produce valid p-values and confidence interval. If significant deviations from required distributions in study data are detected, we will employ negative-binomial or zero-inflated negative-binomial models to account for the observed pattern of data. Comparisons of incidence measures will be expressed at the incidence rate ratio (IRR) or the protective efficacy (PE = 1-IRR x 100%). A skeleton table for the presentation of safety and tolerability outcomes are presented in Tables 13 below.

**Table 13. Measures of safety and tolerability**

| **Outcome** |  |  |  |  |
| --- | --- | --- | --- | --- |
|  | **DP every 12 weeks^a^** | **DP every 4 weeks** | | |
| **Prevalence measures** | **Prevalence** | **Prevalence** | **RR (95% CI)** | **p-value** |
| Vomiting following administration of study drugs  Observed after administration of 1^st^ dose in clinic  Reported after administration of 2^nd^ dose at home  Reported after administration of 3^rd^ dose at home | n/N (%)  n/N (%)  n/N (%) | n/N (%)  n/N (%)  n/N (%) |  |  |
| **Incidence measures** | **Events^b^** | **Events^b^** | **IRR (95% CI)** | **p-value** |
| Individual adverse events of any severity^c^  XXXX  XXXX  XXXX  XXXX  XXXX | xx (x.xx)  xx (x.xx)  xx (x.xx)  xx (x.xx)  xx (x.xx) | xx (x.xx)  xx (x.xx)  xx (x.xx)  xx (x.xx)  xx (x.xx) |  |  |
| Individual grade 3-4 adverse events^c^  XXXX  XXXX  XXXX  XXXX  XXXX | xx (x.xx)  xx (x.xx)  xx (x.xx)  xx (x.xx)  xx (x.xx) | xx (x.xx)  xx (x.xx)  xx (x.xx)  xx (x.xx)  xx (x.xx) |  |  |
| All grade 3-4 adverse events | xx (x.xx) | xx (x.xx) |  |  |
| Grade 3-4 adverse events possibly related to stud drugs | xx (x.xx) | xx (x.xx) |  |  |
| All serious adverse events | xx (x.xx) | xx (x.xx) |  |  |

**^a^** Reference group

**^b^** Number of events (incidence per person year at risk)

**^c^** Includes only those categories with at least five total events

# References

1. WHO. World Malaria Report, 2015. Geneva, Switzerland: World Health Organization; 2015.

2. Dellicour S, Tatem AJ, Guerra CA, Snow RW, ter Kuile FO. Quantifying the number of pregnancies at risk of malaria in 2007: a demographic study. PLoS Med 2010;7:e1000221.

3. Desai M, ter Kuile FO, Nosten F, et al. Epidemiology and burden of malaria in pregnancy. Lancet Infect Dis 2007;7:93-104.

4. Walker PG, ter Kuile FO, Garske T, Menendez C, Ghani AC. Estimated risk of placental infection and low birthweight attributable to Plasmodium falciparum malaria in Africa in 2010: a modelling study. Lancet Glob Health 2014;2:e460-7.

5. Harrington WE, Mutabingwa TK, Kabyemela E, Fried M, Duffy PE. Intermittent treatment to prevent pregnancy malaria does not confer benefit in an area of widespread drug resistance. Clinical infectious diseases : an official publication of the Infectious Diseases Society of America 2011;53:224-30.

6. Menendez C, Bardaji A, Sigauque B, et al. A randomized placebo-controlled trial of intermittent preventive treatment in pregnant women in the context of insecticide treated nets delivered through the antenatal clinic. PLoS ONE 2008;3:e1934.

7. Kakuru A, Jagannathan P, Muhindo MK, et al. Dihydroartemisinin–Piperaquine for the Prevention of Malaria in Pregnancy. New England Journal of Medicine 2016;374:928-39.

8. Desai M, Gutman J, L'Lanziva A, et al. Intermittent screening and treatment or intermittent preventive treatment with dihydroartemisinin-piperaquine versus intermittent preventive treatment with sulfadoxine-pyrimethamine for the control of malaria during pregnancy in western Kenya: an open-label, three-group, randomised controlled superiority trial. Lancet 2015;386:2507-19.

9. Dauby N, Goetghebuer T, Kollmann TR, Levy J, Marchant A. Uninfected but not unaffected: chronic maternal infections during pregnancy, fetal immunity, and susceptibility to postnatal infections. The Lancet infectious diseases 2012;12:330-40.

10. Adegnika AA, Kohler C, Agnandji ST, et al. Pregnancy-associated malaria affects toll-like receptor ligand-induced cytokine responses in cord blood. The Journal of infectious diseases 2008;198:928-36.

11. Ismaili J, van der Sande M, Holland MJ, et al. Plasmodium falciparum infection of the placenta affects newborn immune responses. Clin Exp Immunol 2003;133:414-21.

12. Malhotra I, Mungai P, Muchiri E, et al. Distinct Th1- and Th2-Type prenatal cytokine responses to Plasmodium falciparum erythrocyte invasion ligands. Infect Immun 2005;73:3462-70.

13. Metenou S, Suguitan AL, Jr., Long C, Leke RG, Taylor DW. Fetal immune responses to Plasmodium falciparum antigens in a malaria-endemic region of Cameroon. J Immunol 2007;178:2770-7.

14. Bardaji A, Sigauque B, Sanz S, et al. Impact of malaria at the end of pregnancy on infant mortality and morbidity. The Journal of infectious diseases 2011;203:691-9.

15. Le Hesran JY, Cot M, Personne P, et al. Maternal placental infection with Plasmodium falciparum and malaria morbidity during the first 2 years of life. Am J Epidemiol 1997;146:826-31.

16. Malhotra I, Dent A, Mungai P, et al. Can prenatal malaria exposure produce an immune tolerant phenotype? A prospective birth cohort study in Kenya. PLoS medicine 2009;6:e1000116.

17. Mutabingwa TK, Bolla MC, Li JL, et al. Maternal malaria and gravidity interact to modify infant susceptibility to malaria. PLoS medicine 2005;2:e407.

18. Schwarz NG, Adegnika AA, Breitling LP, et al. Placental malaria increases malaria risk in the first 30 months of life. Clinical infectious diseases : an official publication of the Infectious Diseases Society of America 2008;47:1017-25.

19. Rogier C. Natural history of Plasmodium falciparum malaria and determining factors of the acquisition of antimalaria immunity in two endemic areas, Dielmo and Ndiop (Senegal). Bulletin et memoires de l'Academie royale de medecine de Belgique 2000;155:218-26.

20. White NJ. Intermittent presumptive treatment for malaria. PLoS Med 2005;2:e3.

21. Snow RW, Craig M, Deichmann U, Marsh K. Estimating mortality, morbidity and disability due to malaria among Africa's non-pregnant population. Bull World Health Organ 1999;77:624-40.

22. Rogier C, Tall A, Diagne N, Fontenille D, Spiegel A, Trape JF. Plasmodium falciparum clinical malaria: lessons from longitudinal studies in Senegal. Parassitologia 1999;41:255-9.

23. Ramphul U, Boase T, Bass C, Okedi LM, Donnelly MJ, Muller P. Insecticide resistance and its association with target-site mutations in natural populations of Anopheles gambiae from eastern Uganda. Transactions of the Royal Society of Tropical Medicine and Hygiene 2009;103:1121-6.

24. Verhaeghen K, Bortel WV, Roelants P, Okello PE, Talisuna A, Coosemans M. Spatio-temporal patterns in kdr frequency in permethrin and DDT resistant Anopheles gambiae s.s. from Uganda. The American journal of tropical medicine and hygiene 2010;82:566-73.

25. Lengeler C. Insecticide-treated bed nets and curtains for preventing malaria. Cochrane database of systematic reviews (Online) 2004:CD000363.

26. Trape JF, Tall A, Diagne N, et al. Malaria morbidity and pyrethroid resistance after the introduction of insecticide-treated bednets and artemisinin-based combination therapies: a longitudinal study. The Lancet infectious diseases 2011;11:925-32.

27. Jagannathan P, Muhindo MK, Kakuru A, et al. Increasing incidence of malaria in children despite insecticide-treated bed nets and prompt anti-malarial therapy in Tororo, Uganda. Malar J 2012;11:435.

28. Aponte JJ, Schellenberg D, Egan A, et al. Efficacy and safety of intermittent preventive treatment with sulfadoxine-pyrimethamine for malaria in African infants: a pooled analysis of six randomised, placebo-controlled trials. Lancet 2009;374:1533-42.

29. Chandramohan D, Owusu-Agyei S, Carneiro I, et al. Cluster randomised trial of intermittent preventive treatment for malaria in infants in area of high, seasonal transmission in Ghana. Bmj 2005;331:727-33.

30. Kobbe R, Kreuzberg C, Adjei S, et al. A randomized controlled trial of extended intermittent preventive antimalarial treatment in infants. Clin Infect Dis 2007;45:16-25.

31. Macete E, Aide P, Aponte JJ, et al. Intermittent preventive treatment for malaria control administered at the time of routine vaccinations in mozambican infants: a randomized, placebo-controlled trial. J Infect Dis 2006;194:276-85.

32. Mockenhaupt FP, Reither K, Zanger P, et al. Intermittent preventive treatment in infants as a means of malaria control: a randomized, double-blind, placebo-controlled trial in northern Ghana. Antimicrob Agents Chemother 2007;51:3273-81.

33. Schellenberg D, Menendez C, Kahigwa E, et al. Intermittent treatment for malaria and anaemia control at time of routine vaccinations in Tanzanian infants: a randomised, placebo-controlled trial. Lancet 2001;357:1471-7.

34. Odhiambo FO, Hamel MJ, Williamson J, et al. Intermittent preventive treatment in infants for the prevention of malaria in rural Western kenya: a randomized, double-blind placebo-controlled trial. PLoS ONE 2010;5:e10016.

35. Dicko A, Diallo AI, Tembine I, et al. Intermittent preventive treatment of malaria provides substantial protection against malaria in children already protected by an insecticide-treated bednet in Mali: a randomised, double-blind, placebo-controlled trial. PLoS Med 2011;8:e1000407.

36. Konate AT, Yaro JB, Ouedraogo AZ, et al. Intermittent preventive treatment of malaria provides substantial protection against malaria in children already protected by an insecticide-treated bednet in Burkina Faso: a randomised, double-blind, placebo-controlled trial. PLoS Med 2011;8:e1000408.

37. Wilson AL. A systematic review and meta-analysis of the efficacy and safety of intermittent preventive treatment of malaria in children (IPTc). PLoS ONE 2011;6:e16976.

38. Inaugural meeting of the malaria policy advisory committee to the WHO: conclusions and recommendations. Malaria journal 2012;11:137.

39. Organization WH. World Malaria Report 20112011.

40. A head-to-head comparison of four artemisinin-based combinations for treating uncomplicated malaria in African children: a randomized trial. PLoS medicine 2011;8:e1001119.

41. Arinaitwe E, Sandison TG, Wanzira H, et al. Artemether-lumefantrine versus dihydroartemisinin-piperaquine for falciparum malaria: a longitudinal, randomized trial in young Ugandan children. Clin Infect Dis 2009;49:1629-37.

42. Kamya MR, Yeka A, Bukirwa H, et al. Artemether-lumefantrine versus dihydroartemisinin-piperaquine for treatment of malaria: a randomized trial. PLoS Clin Trials 2007;2:e20.

43. Yeka A, Dorsey G, Kamya MR, et al. Artemether-lumefantrine versus dihydroartemisinin-piperaquine for treating uncomplicated malaria: a randomized trial to guide policy in Uganda. PloS one 2008;3:e2390.

44. Lwin KM, Phyo AP, Tarning J, et al. Randomized, double-blind, placebo-controlled trial of monthly versus bimonthly dihydroartemisinin-piperaquine chemoprevention in adults at high risk of malaria. Antimicrobial agents and chemotherapy 2012;56:1571-7.

45. Bigira V, Kapisi J, Clark TD, et al. Protective efficacy and safety of three antimalarial regimens for the prevention of malaria in young ugandan children: a randomized controlled trial. PLoS Med 2014;11:e1001689.

46. Greenwood BM, David PH, Otoo-Forbes LN, et al. Mortality and morbidity from malaria after stopping malaria chemoprophylaxis. Trans R Soc Trop Med Hyg 1995;89:629-33.

47. Menendez C, Kahigwa E, Hirt R, et al. Randomised placebo-controlled trial of iron supplementation and malaria chemoprophylaxis for prevention of severe anaemia and malaria in Tanzanian infants. Lancet 1997;350:844-50.

48. Sundell K, Jagannathan P, Huang L, et al. Variable piperaquine exposure significantly impacts protective efficacy of monthly dihydroartemisinin-piperaquine for the prevention of malaria in Ugandan children. Malar J 2015;14:368.

49. Jagannathan P, Bowen K, Nankya F, et al. Effective Antimalarial Chemoprevention in Childhood Enhances the Quality of CD4+ T Cells and Limits Their Production of Immunoregulatory Interleukin 10. J Infect Dis 2016;214:329-38.

50. Belnoue E, Costa FT, Frankenberg T, et al. Protective T cell immunity against malaria liver stage after vaccination with live sporozoites under chloroquine treatment. J Immunol 2004;172:2487-95.

51. Belnoue E, Voza T, Costa FT, et al. Vaccination with live Plasmodium yoelii blood stage parasites under chloroquine cover induces cross-stage immunity against malaria liver stage. J Immunol 2008;181:8552-8.

52. Friesen J, Silvie O, Putrianti ED, Hafalla JC, Matuschewski K, Borrmann S. Natural immunization against malaria: causal prophylaxis with antibiotics. Sci Transl Med 2010;2:40ra9.

53. Roestenberg M, McCall M, Hopman J, et al. Protection against a malaria challenge by sporozoite inoculation. The New England journal of medicine 2009;361:468-77.

54. Roestenberg M, Teirlinck AC, McCall MB, et al. Long-term protection against malaria after experimental sporozoite inoculation: an open-label follow-up study. Lancet 2011;377:1770-6.

55. Kakuru A, Jagannathan P, Muhindo MK, et al. Dihydroartemisinin-Piperaquine for the Prevention of Malaria in Pregnancy. N Engl J Med 2016;374:928-39.
